# Helminth/Protozoan Coinfections in Chronic Fascioliasis Cases in Human Hyperendemic Areas: High Risk of Multiparasitism Linked to Transmission Aspects and Immunological, Environmental and Social Factors

**DOI:** 10.3390/tropicalmed10080224

**Published:** 2025-08-11

**Authors:** M. Adela Valero, M. Manuela Morales-Suarez-Varela, Davis J. Marquez-Guzman, Rene Angles, Jose R. Espinoza, Pedro Ortiz, Filippo Curtale, M. Dolores Bargues, Santiago Mas-Coma

**Affiliations:** 1 Departamento de Parasitología, Facultad de Farmacia, Universidad de Valencia, Av. Vicent Andrés Estellés s/n, Burjassot, 46100 Valencia, Spain; damarguz@alumni.uv.es (D.J.M.-G.); m.d.bargues@uv.es (M.D.B.); s.mas.coma@uv.es (S.M.-C.); 2 CIBER de Enfermedades Infecciosas, Instituto de Salud Carlos III, C/ Monforte de Lemos 3–5, Pabellón 11, Planta 0, 28029 Madrid, Spain; 3 Departamento de Medicina Preventiva y Salud Pública, Centro Superior de Investigación en Salud Pública (CSISP), Facultad de Farmacia, Universidad de València, 46010 Valencia, Spain; maria.m.morales@uv.es; 4 CIBER Epidemiology and Public Health, Instituto de Salud Carlos III, C/ Monforte de Lemos 3–5, Pabellón 11, Planta 0, 28029 Madrid, Spain; 5 Cátedra de Parasitología, Facultad de Medicina, Universidad Mayor de San Andrés (UMSA), Av. Saavedra, Miraflores, La Paz 1010, Bolivia; anglesrene@hotmail.es; 6 Department of Engineering, Universidad Peruana Cayetano Heredia, Av. Honorio Delgado 430, San Martin de Porres, Lima 15102, Peru; jose.espinoza@upch.pe; 7 Facultad de Ciencias Veterinarias, Universidad Nacional de Cajamarca, Av. Atahualpa 1050, Cajamarca 06003, Peru; portiz@unc.edu.pe; 8 U.O.C. Rapporti Internazionali, con le Regioni e Gestione del Ciclo di Progetto, Istituto Nazionale per la Promozione della Salute delle Popolazioni Migranti e il Contrasto delle Malattie della Povertà, 00153 Roma, Italy; filippocurtale@hotmail.com

**Keywords:** fascioliasis, human hyperendemic areas, coinfections, other helminths, protists/protozoans, multiparasitisms, parasite associations, pathogenic implications, transmission routes and infection sources, immunological, environmental and social factors

## Abstract

Research is required to determine whether the coinfections by *Fasciola* spp. and other parasite species result from poor rural hygiene or reflect underlying epidemiological patterns and causes. Therefore, the role of fascioliasis is analyzed concerning coinfection complexity, risk of multiparasitism, parasite associations, pathogenic implications and their multifactorial causes. Helminth and protozoan coinfections are studied in 2575 previously untreated individuals from four rural hyperendemic areas (Northern Bolivian Altiplano, Peruvian Altiplano and Cajamarca valley, and the Egyptian Nile Delta). This cross-sectional study was conducted from January 2011 to December 2023. Coinfections were coprologically assessed by the merthiolate–iodine–formalin and formol–ether concentration techniques. Infection intensity was measured as eggs/gram of feces (epg) with the Kato–Katz technique. Parasite and coinfection prevalences were stratified by age, sex and geographical location. High mixed infections, fascioliasis prevalences and very low non-coinfected *Fasciola*-infected subjects were associated with the following regions: Bolivian Altiplano, 96.5%, 16.8% and 3.5%; Peruvian Altiplano, 100%, 24.6% and 0%; Cajamarca valley, 98.7%, 21.4% and 1.8%; Nile Delta, 84.1%, 13.0% and 15.9%. Transmission routes and human infection sources underlie fascioliasis associations with protozoan and other helminth infections. Prevalence pattern of protozoan–helminth coinfections differed between *Fasciola*-infected individuals and individuals not infected with *Fasciola*, presenting higher prevalences in individuals with fascioliasis. Multiparasitism diagnosed in *Fasciola*-infected subjects included coinfections by up to nine parasite species, eight protozoan species, and five helminth species. The most prevalent pathogenic protozoan was *Giardia intestinalis*. The most prevalent helminth species differ according to environmental conditions. Several parasites indicate fecal environmental contamination. When the fascioliasis burden increases, the total number of parasite species also increases. The fascioliasis risk increases when the total helminth species number/host increases. *Fasciola*-infected subjects may present a modification in the clinical phenotypes of coinfecting parasitic diseases. Fascioliasis coinfection factors include transmission ways and immunological, environmental and social aspects. Coinfections must be considered when assessing the health impact of fascioliasis, including the analysis of the fascioliasis effects on malnutrition and physical/intellectual child development. Fascioliasis-control schemes should, therefore, integrate control measures mainly against other helminthiases.

## 1. Introduction

Fascioliasis is a pathogenic zoonotic disease of worldwide distribution caused by the liver flukes *Fasciola hepatica* and *F. gigantica* [1]. Both species originated by speciation derived from the hippopotamus-restricted species *F. nyanzae* [2] and show a relative low host specificity, as illustrated by their capacity to infect the livers of a wide range of mammal reservoirs [3,4,5,6], mainly livestock. The impact of this disease on animal husbandry is well known [7]. Liver flukes have freshwater lymnaeid snails as vectors, mainly species of the globally distributed *Galba/Fossaria* group transmitting *F. hepatica*, as well as species of the *Radix* group, restricted to the Old World in the case of *F. gigantica* [1,2]. This snail vector specificity underlies the global distribution of *F. hepatica* and explains why *F. gigantica* never colonized the Americas [8].

Human fascioliasis was found to be a pronouncedly emerging disease in Latin America, northern Africa and near-eastern Asia during the 1990s and 2000s [9,10], and is at present emerging in southern and southeastern Asia [11,12,13,14,15,16]. Currently, fascioliasis continues to infect humans and poses an emerging risk in high-income countries [17]. It is therefore recognized as a serious public health problem in many parts of the world [18], posing worrying implications given that it is the only human foodborne trematodiasis of global distribution [1]. Several areas in Central and South America, Europe, Africa and Asia are endemic for the human infection, ranging from hypoendemic to hyperendemic situations [19].

In the 1990s and 2000s, given the progressive reporting of human endemic areas in different continents, with a growing number of human cases, the World Health Organization (WHO) decided to include fascioliasis in the group of foodborne trematodiases, among neglected tropical diseases (NTD) [20,21]. According to the WHO NTD Roadmap [21], preventive chemotherapy is applied in endemic areas, where infections and re-infections in-between yearly campaigns pose problems due to the absence of post-treatment immunity in fascioliasis [22,23], i.e., absence of sterile immunity [24]. Therefore, multidisciplinary “One Health” control initiatives have been shown to be the best complements of the chemotherapy campaigns, in aiming to reduce the risk of infection and re-infection after and in-between annual mass treatments [24].

This disease in humans evolves according to two main different phases: (i) the initial 3–4-month long acute phase, corresponding to the parasite migration from the intestine up to the abdominal cavity and liver parenchyma; and (ii) the subsequent, up to 13.5-year-long chronic phase, when the parasite has settled in the lumen of the biliary canals and gallbladder [10]. In humans infected in fascioliasis hyperendemic rural areas of low-income countries, the liver fluke may continue to infect subjects for long periods of time, reaching the advanced chronic stage of the disease. Both the chronic and advanced chronic stages are evidenced mainly by the presence of eggs in feces. The advanced chronic stages encompass an obstructive phase which may develop after months to years of infection, including mild–moderate to severe pathogenicity, especially in cases of heavy parasitic burdens [22,25]. Clinical studies have shown fascioliasis to be pronouncedly complex, giving rise to progressive general deterioration of the patients, with sequelae even in treated patients [26], sometimes leaving subjects handicapped and frail, and including fatal cases [25,27,28,29]. The worrying impacts of neurological, meningeal, neuropsychic and ophthalmological disorders in fascioliasis-infected subjects, hitherto neglected despite their pathological effects and the very wide geographical distribution of affected patients [29], should also be considered. Alterations of the physiological systems underlying the origin of such disorders due to the liver fluke infection have recently been elucidated in both the acute and the chronic phases [30,31].

In human hyperendemic areas, children and adult subjects are easily diagnosed by the detection of fluke eggs in stools, as most of the infected subjects are in the chronic phase. Moreover, protozoan/helminthic coinfections in liver fluke-infected subjects are frequent. Summing up, chronicity and coinfection are the norm in human fascioliasis hyperendemic areas. Although little is known about fascioliasis coinfections in humans, the phenomenon has been suggested to be linked to a combined morbidity potential of concern and to high pathogenicity and severe clinical pictures in patients in endemic areas. The importance of multiparasitism and the impact of coinfections [32,33,34], as well as the problems they pose regarding the appropriate way of accounting for the evaluation of the morbidities and true costs of parasitic diseases [35], have already been highlighted.

Within the same host individual, parasite interactions can be: (i) synergistic, that is, the presence of one parasite can facilitate subsequent infections by other parasites; or (ii) antagonistic, that is, the presence of one parasite can inhibit posterior infections by other parasites. In parasitic coinfections of protozoans and helminths, both agonistic and antagonistic interactions have been reported [36,37,38]. Experimental studies in fascioliasis animal models indicate that there are synergistic and antagonistic interactions with other pathogens. Thus, *Fasciola*/paramphistomes coinfection in large ruminants suggests that infection with one of these parasites increases the chance of infection by the other [39,40], while *F. hepatica/Echinococcus granulosus* coinfection in cattle has a detrimental effect on echinococcal cysts, decreasing the average intensity of the number of cysts per animal, the fertility of the cysts [41,42] and even modifying the hydatid cyst location [43].

Like many other parasites, *F. hepatica* and *F. gigantica* produce immunoregulatory changes in their hosts. Although fascioliasis affects the host’s capacity to react against the development of other concomitant pathogen infections in animal models, this phenomenon has so far not been corroborated in human studies. Extrapolation of the results obtained in animals to human infection gives a new dimension to chronic fascioliasis, namely, the possibility of individuals acquiring concomitant parasitic infections, which has been observed to be common in human fascioliasis endemic areas.

In fascioliasis patients, many simultaneous infections by other infectious agents have been reported. Among helminths, fascioliasis/hydatidosis coinfections in patients appear to be the most numerous by far [44,45,46,47,48,49,50,51,52,53,54]. Other helminth species reported to be found coinfecting fascioliasis patients include the trematodes *Clonorchis sinensis* [55] and *Opisthorchis viverrini* [56], and the cestode *Taenia saginata* [57]. Among nematodiases, the following coinfections have been reported: trichinellosis [58], strongyloidiasis [59] and toxocariasis; the last is of pathogenic relevance [60], including even neuro-affection [61].

Coinfection of *Fasciola* with other helminth species has also been reported in studies performed in endemic areas other than those focused on the present article. In children, coinfecting helminths such as *Ascaris lumbricoides* and *Ancylostoma duodenale* in northern Pakistan [13] and *Hymenolepis nana*, *A. lumbricoides* and *Trichuris trichiura* in central Mexico [62] have been reported. It is worth mentioning that the latter study demonstrated that coinfection by moderate burdens of *A. lumbricoides* was related to children needing a second treatment course and thus suggested an interaction with the efficacy of the drug.

Several protozoan species, and the stramenopile *Blastocystis* sp. of the SAR group (here included within Protozoa for simplification throughout), have also been reported to coinfect patients infected by *Fasciola* flukes, as, for instance: *Entamoeba coli* and *Endolimax nana* [57,63]; *Entamoeba histolytica* complex (including *E. histolytica*, *E. dispar*, *E. moshkovskii* and *E. bangladeshi*) [60,62]; *Trypanosoma cruzi* [59]; *Giardia intestinalis* [59,62]; and *Blastocystis* sp. [57,62].

The interactions between *Toxoplasma gondii* and fascioliasis have been immunologically analyzed within the same host individual [64]. In coinfections of fascioliasis, reports have included bacterial infections such as brucellosis [65,66] and tuberculosis by *Mycobacterium* [67], and also viroses such as hepatitis [68].

It is evident that these coinfections with fascioliasis make further studies necessary to assess whether they are simply due to hazards in rural areas lacking hygienic conditions, or, on the contrary, they follow given patterns as the consequences of underlying causes. To assess the potential role of fascioliasis in such coinfections, we transversally study data on the epidemiology of human parasites (helminths and protozoans) and *Fasciola* infections in a total of 2575 non-previously treated individuals from four human hyperendemic areas in which most of the liver fluke-infected inhabitants prove to be mainly in the advanced stage of chronicity: the northern Bolivian Altiplano, the Peruvian Altiplano, the Cajamarca valley of Peru and the lowland areas of the Nile Delta in Egypt. This comparative study concerning fascioliasis, as well as the associated coinfection complexity and possible combinations, risk of multiparasitism, parasite associations and pathogenic implications and their multifactorial causes, is made for the first time. Understanding the proportion of patients with fascioliasis with protozoan/helminth coinfection is crucial in the responsible use of antiparasitic drugs and design of prevention measures.

By performing a retrospective analysis by the WHO Reference Centre for Fascioliasis and its Snail Vectors about data on fascioliasis over a 13-year period, we aimed to explore the causal and epidemiological characteristics by sex, age and region, to better describe community patterns of fascioliasis and their association with coinfections, which in turn might help refine evidence-based strategies to reduce morbidity and mortality.

## 2. Materials and Methods

### 2.1. Ethics Statement

All investigations were made after permission was obtained from local authorities or community chiefs in the rural villages, as well as from the director and teachers at each school. All surveys were performed with the consent of the subjects or, in the case of young children, their parents or guardians, and epidemiological analyses were made according to agreements previously reached with the Ministries of Health of each one of the countries of Bolivia, Peru and Egypt.

Collection, management and analysis of biological samples were carried out according to the Ethical Protocol approved by the Comité Etico de Investigación en Humanos de la Universidad de Valencia, Valencia, Spain (Approval No. H1496156195013, 1 June 2017), and the Ethical Protocol by the Comisión de Etica en Investigación Experimental de la Universidad de Valencia (Approval No. A1263915389140, 22 July 2010). In addition, in Bolivia, the study was approved by the Comisión de Etica de la Investigación of the Comité Nacional de Bioética, La Paz (Certificate dated 10 September 2007), Comité de Etica y Bioética de la Facultad de Medicina de la Universidad Mayor de San Andres, UMSA, La Paz—COMETICA (Resolución COMETICA No. 03/2019, dated 23 July 2019), and Comité de Revisión Etica (PAHOERC) of the Pan American Health Organization, PAHO, Washington DC (Dictamen Ref. No. 2018-02-0007, dated 10 September 2019).

### 2.2. Study Design

Previous analyses relating to human fascioliasis in hyperendemic areas were performed under the leadership of the WHO Reference Centre for Fascioliasis and its Snail Vectors in four regions in Bolivia, Peru and Egypt. The present cross-sectional study involved data analysis and incorporated a total of 2575 individuals from four human hyperendemic areas diagnosed by the aforementioned WHO Collaborating Center. The studies were part of an epidemiological initiative focused on new hyperendemic areas by means of classical coprological studies. The number of participants in this work was reduced compared to participant numbers in the previously published studies due to the lack of information on key covariates, such as sex and age, or a detailed composition of coinfection.

To maintain the same terminology that we used in our previous articles, the term “survey” is here used throughout to refer to a field study performed to detect the children or subjects infected by *Fasciola* among the total number of children or subjects that could be diagnosed in the cross-sectional study of a school or locality at a specific point in time.

Similarly, we here use throughout the term “prevalence” to refer to the percentage of liver fluke-infected subjects among the total of subjects analyzed in the school (when only schoolchildren) or rural village (when the survey focused on total population). In these surveys, the numbers of subjects analyzed were always ensured to be of sufficient size as to be representative of the respective school or village (see below for summaries of studies performed in each endemic area).

### 2.3. Study Population

This study includes a total of 2575 subjects whose biological samples for diagnosis were obtained in surveys of rural areas where fascioliasis is transmitted.

Diagnostic samples only concern fecal samples, which means that the coinfection assessments only refer to parasitoses using stools for the shedding of the transmission stages, including parasites whose microhabitat is part of the digestive system and related glands as liver and pancreas, but also those affecting the lungs and pulmonary ducts connected to the upper digestive tract.

Samplings were performed in human settlements and schools, the inhabitants or children of which had not been previously treated, either at the individual level after diagnosis of infection, or at community level following preventive chemotherapy; i.e., none of the surveys was performed on populations composed of subjects included in previous preventive chemotherapy campaigns, such as those with albendazole or triclabendazole, which have been frequently applied in recent decades. To ensure that the sample would be sufficient, a stool sample from each child of each rural school was obtained on the day of the survey and, when analyzing total population in the small rural villages, all inhabited houses were visited and stool samples collected the day after.

The diagnosis of infection by *Fasciola* flukes was made by the finding of the fasciolid eggs in the stool samples, which means that the respective subjects were in the chronic phase of the disease. Schools and localities were targeted by considering their evident liver fluke infection risk due to the nearness of disease transmission foci (freshwater collections inhabited by lymnaeid snail vectors) and the close presence of livestock (domestic ruminants known to be fascioliasis reservoirs). This does not mean, however, that other schools and localities were also studied, in aiming to assess the traditionally patchy distribution of this waterborne disease throughout subareas inside the endemic area. This explains the wide range of prevalence values corresponding to the schools and localities in each endemic area.

Subjects included in this coinfection study were only those who provided a sufficient amount of stool to allow for the application of all the coprological diagnostic techniques to the same sample and provided all the needed individual information (sometimes difficult in very small children), which explains why the number of subjects in this study involved fewer subjects than the total number of persons originally surveyed.

Subjects involved in the surveys were given a small container and requested to defecate and directly deposit the stool sample obtained inside it that same morning, to avoid receiving a stool sample not belonging to the subject in question but to another family member when the containers were distributed the day before (a well-known problem in several rural communities of Bolivia and Peru, mainly when dealing with girls and women in cases of certain ethnicities and beliefs).

### 2.4. Sampling Strategy

In humans, (i) the only way to differentiate between the acute phase (3–4 months) and the chronic phase of the disease (up to 13.5 years in humans) is by coprological examination. A coprological sample allows for (ii) the detection of the infection by *Fasciola* and by all other protozoa and helminths which use the anal route for their propagation. (iii) Sampling of fecal samples is easily feasible in rural areas, and (iv) does not require a laboratory provided with the expensive equipment needed for the serological testing in close proximity, a situation which usually does not exist in such remote areas.

Serological tests are not appropriate for the differentiation of the acute and chronic phases, because these tests become positive already from the second month, and they need at least six months to negativize after deworming and are therefore useless for treatment monitoring. Accurate field surveys for fascioliasis prevalence analyses should rely on coprology, for both cross-sectional and longitudinal studies. This is why in our coinfection analyses we always refer to subjects presenting with fascioliasis chronicity, i.e., shedding eggs in stools. Moreover, serological tests are not quantitative and are therefore useless for individual burden estimation, as verified in human endemic areas, and consequently they cannot be used for establishing treatment course and dose to avoid potential colics.

In addition, it should be considered that in remote rural areas with specific religious beliefs, as for instance with the Aymaras of the Bolivian Altiplano, the inhabitants do not allow blood sampling and as such, serological tests cannot be used. This is why coprological analyses are the only way of obtaining samples for diagnosis of liver fluke infection allowing for comparisons between endemic areas from different countries.

Spurious infection in human fascioliasis is easily detected by an experienced microscopist. Fasciolid eggs do not have a resistant external shell and become modified during their transit through the stomach and whole intestine, finally leading to a visible egg-shell degeneration. No case of spurious infection was found in the studied areas, which is easily understandable because liver is not usual in the local diet, and such a possible detection is time-wise very sporadic, because for the detection of such eggs in transit the fecal sample should be obtained shortly after liver ingestion. All these studies were performed by specialists for whom spurious infection is a basic aspect of their knowledge about fascioliasis.

Imaging techniques such as US, TAC or MNR, or any other, are far from being considered pathognomonic in fascioliasis, i.e., the images may easily be confused with other causes, e.g., amebiasis, etc. They may help but are only suggestive and should be confirmed by egg finding or a specific serotest.

### 2.5. Study Areas

**Figure 1:**
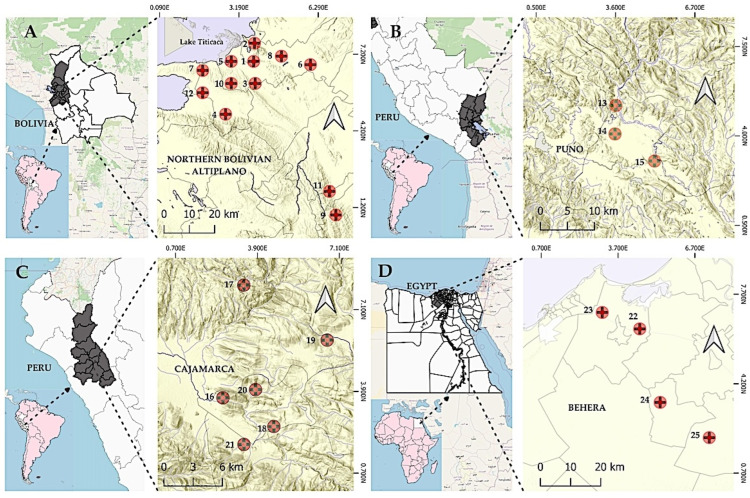
Map showing the distribution of the study locations. (**A**) Northern Bolivian Altiplano: (1) Aygachi, (2) Belén Yayes, (3) Calería, (4) Causaya, (5) Cohana, (6) Corapata, (7) Huacullani, (8) Iquiaca, (9) Kajchiri, (10) Quiripujo, (11) Tuni and (12) Yanarico. (**B**) Puno, Peruvian Altiplano: (13) Jila, (14) Accopata and (15) Naupapampa. (**C**) Cajamarca Valley, Peru: (16) Huayrapongo Grande, (17) La Colpa, (18) Llimbe, (19) localidad de Santa Rosa de Chaquil, (20) Shaullo Grande and (21) Yanamango. (**D**) Behera, Nile Delta, Egypt: (22) El Aaly, (23) Bolin, (24) El Kaza and (25) Tiba.

The aforementioned four fascioliasis endemic areas are catalogued as human hyperendemic areas, according to the epidemiological classification of WHO [69]. In the three Andean areas (Bolivian Altiplano, Peruvian Altiplano and Peruvian Cajamarca valley) only *F. hepatica* is present, whereas in the area surveyed in Egypt (Nile Delta) both *F. hepatica* and *F. gigantica* coexist (Figure 1).

The sample size of 2575 individuals is the result of restricting this analysis to subjects from whom all the key information could be obtained, concerning both (i) coprological techniques applied allowing for the detection of all potential protozoans and helminths using transmission forms shed with stools, and (ii) all essential individual information about key variables such as sex, age and place of origin.

The subjects were inhabitants in rural human hyperendemic areas with pronouncedly different epidemiological characteristics and different fascioliasis transmission patterns:

- One *Fasciola* species or the two *Fasciola* species.
- Only one lymnaeid snail vector species or more than one involved in the transmission.
- Seasonal or year-long permanent transmission.
- With or without involvement of pigs, buffaloes and goats as animal reservoirs, in addition to sheep and cows.
- Ranging from very high altitude to lowlands at sea level.
- Ranging from frequently to rarely occurring human infection source by natural water drinking.
- With different influences of behavioral, traditional, social and religious aspects linked to the disease transmission and human infection sources.

The differences in the ethnographic characteristics of the inhabitants of these four endemic areas do not allow for similar data collection and control strategies. As already highlighted by WHO, field studies and control interventions on human fascioliasis should adapt to the local circumstances and intervention feasibilities. It is for this reason that (i) in Egypt we used active detection of infected subjects and subsequent individualized treatments, which was feasible thanks to the existence of groups of officers of the Health Ministry with long field expertise on schistosomiasis (another waterborne trematodiasis transmitted in the same local foci), whereas (ii) in neither Bolivia nor Peru do such health officer groups exist, and therefore the control is based on yearly mass drug treatments. Additionally, (iii) for instance, in Vietnam we used passive detection of infected subjects in hospitals after appropriate diffusion by radio broadcasting. These are unavoidable conditions to which the sampling strategies should adapt to successfully attain the study’s goals.

The characteristics of the study populations in the four areas surveyed, including the knowledge relevant to the transmission and epidemiology of the disease, are described in Table 1 and detailed in Supplementary Materials File S1: Northern Bolivian Altiplano (Bolivia) [3,4,5,6,19,24,70,71,72,73,74,75,76,77,78,79,80,81,82], Peruvian Altiplano (Peru) [9,19,83,84,85,86,87,88], Cajamarca valley (Peru) [78,84,89,90,91,92,93,94,95,96,97,98,99,100,101,102,103,104,105,106,107] and Behera Governorate in the Nile Delta (Egypt) [84,99,108,109,110,111,112,113,114,115,116].

### 2.6. Laboratory Procedures

One stool sample was collected per subject and personal data (name, sex and age) were recorded at the time of container delivery. Fecal samples were transported to the laboratory within 1 to 3 h after collection. Once in the laboratory, consistency (formed, soft, loose, or watery) of each stool sample was evaluated and noted. The following techniques were used for the diagnostic analysis of each stool:

- Two aliquots of each stool sample were preserved, one in merthiolate–iodine–formalin (MIF) fixative (1:3) and one in 10% formalin solution (1:3) [117].
- Samples fixed in MIF were processed using the direct smear [118] and MIF concentration techniques [119].
- Samples fixed in 10% formalin were processed by a formol–ether concentration technique [120] and, whenever possible, the sediment was stained using a modified Ziehl–Neelsen technique to facilitate the detection of the very small *Cryptosporidium* oocysts by coloration [121,122].
- Additionally, depending on the stool amount available, up to three Kato–Katz slides [123,124] were made from each stool sample to compensate for the well-known low sensitivity of this technique [125]. Kato–Katz slides were not only used to assess prevalences, but also for the analysis of egg counts.

Infection intensity was measured as eggs/gram of feces (epg) as an indicator of liver fluke burden in infected subjects. Each stool sample was examined by at least two expert microscopists with the aforementioned techniques.

### 2.7. Outcomes and Statistical Methods

The primary outcome was to evaluate the pattern of infection by *Fasciola* spp. and coinfection by other parasite species among the individuals studied. The secondary objective was to evaluate the pattern of the fasciolid infection intensity (based on epg) and coinfection among the individuals studied.

Laboratory data from all human samples were standardized to ensure consistency. Analyses were conducted by the same group of scientists. Descriptive statistics included absolute and relative frequencies (proportions) with 95% confidence interval (95% CI) for categorical variables, and medians with interquartile range (IQR) for continuous variables. Parasite species were analyzed based on geographically stratified data. Fasciolid infection data were stratified according to geographical location, age and sex. In addition, the number of coinfecting parasite species was evaluated. Comparisons of parasite species proportions in monoinfection and coinfection were made by stratifying data into *Fasciola*-infected and *Fasciola*-non-infected groups. Statistical tests such as chi-square or Fisher’s exact test were used for inter-group comparisons.

Descriptive statistics for fasciolid infection intensity included the mean and standard deviation (SD) of egg counts (epg). Fascioliasis loads were compared across age groups and coinfection scenarios using Student’s *t*-test, one-way ANOVA, and the Bonferroni post hoc test.

A logistic regression model was used to examine variables related to fascioliasis coinfection. The odds ratio (OR) was estimated using a multivariate logistic regression [in the context of risk factors, the resulting Exp(B) are estimates of OR]. The reference was a non-fascioliasis group. The fascioliasis risk regarding population characteristics (geographical location, sex and age) and the number of the different protozoan and helminth species in each host individual were studied by multivariate logistic regression analysis.

Four models (Models 1–4) were used in the multivariate logistic regression analysis, including presence/absence of fascioliasis as dependent variable (primary outcome). These models aim to avoid potential biases:

- Model 1 included the number of the different protozoan and helminth (helminth number) species in each host as independent variables.
- Model 2 included the population characteristics (geographical location, sex and age) and number of protozoan and helminth (helminth number) species as independent variables.
- Model 3 included the population (age) and number of cases of soil-transmitted helminths (STH: infections caused by the three major species of nematodes, including the roundworm *Ascaris lumbricoides*, the whipworm *Trichuris trichiura* and the hookworms *Ancylostoma duodenale* and *Necator americanus*) as independent variables.
- Model 4 included the population (age), number of cases of *Entamoeba coli*, number of cases of *Entamoeba hartmanni*, number of cases of *Endolimax nana*, number of cases of *Iodamoeba buetschlii*, number of cases of *Giardia intestinalis* and number of cases of STH as independent variables.

Confidence intervals (95% CI) were calculated using EpiData software (version 3.1). Statistical analyses were performed with IBM SPSS statistics (version 28.0.1.1.). Results were considered statistically significant when *p* < 0.05. Figures were produced using GraphPad Prism (version 9.5.1). Heat maps were produced using R software (version 4.4.1.; R Foundation for Statistical Computing, Vienna, Austria, https://cran.r-project.org/ accessed on 3 August 2025).

## 3. Results

### 3.1. Socio-Demographic Characteristics and Associated Factors

Table 1 shows the baseline characteristics of the study population according to geographic location. The sex characteristics and the stratified age are described. When comparing the differences between the two areas in Peru (altiplano and valley), no sex differences (58.4% males in the altiplanic area, 51.4% males in the valley area) and mean age are observed. In terms of age groups, the highest proportion of the population studied was found to be between 7–9 and 10–12 years of age. In these two areas, differences were only detected in the group over 18 years of age (*p* = 0.048).

This Table 1 presents the characteristics of the other two populations, in which Bolivia (56.1% males) presents the highest proportions of the study population between 7–9 and 10–12 years old, and Egypt (33.4% % males) presents the highest proportions of the study population for the groups >18 years and <7 years. The distribution by age group and mean age is also shown. The four zones are compared, displaying the statistically significant differences in age and sex of the population studied.

### 3.2. Intestinal Parasite Species Among People Living in Fascioliasis Hyperendemic Areas

The first aspect to be highlighted is the pronounced similitude of the qualitative (type and total number of protozoan/helminth species detected) compositions of the parasite species infecting the inhabitants of the four fascioliasis hyperendemic areas (Table 2), despite their different epidemiological scenarios and transmission patterns.

In addition to *Fasciola* species, the following parasite species are found in these endemic areas:

- One stramenopile (SAR group): *Blastocystis* sp. (here included within Protozoa);
- Eleven protozoans: see species in Table 2;
- Nine helminths: see species in Table 2.

Within each endemic area, the qualitative compositions are distributed as follows (total parasite species and numbers of stramenopiles, protozoans and helminths): Bolivian Altiplano: 19, 1, 11, 7; Puno Altiplano: 18, 1, 9, 8; Cajamarca valley: 16, 1, 9, 6; and the Nile Delta: 17, 1, 9, 7 (Table 2).

Among protozoans, *D. fragilis* and *Cryptosporidium* sp. were only found in the Bolivian Altiplano. The nine remaining protozoan species appear to be the same in the four endemic areas, similarly to *Blastocystis* sp.

Among helminths, the homogeneity of the species composition is also notable in the four areas, including *Fasciola* spp., *H. nana*, *T. trichiura*, *A. lumbricoides* and *E. vermicularis*. Differences worth mentioning include the presence of *S. mansoni* in the Nile Delta and *S. stercoralis* in the Peruvian Altiplano. Furthermore, the absence of Ancylostomatidae spp. in Cajamarca and *Taenia* spp. in Egypt should also be highlighted.

### 3.3. Analysis of Parasite Species Prevalences and Coinfections

Regarding the quantitative composition, Table 2 shows statistically significant differences in the prevalence of both protozoa and helminths detected among the four geographical areas analyzed. In the four endemic areas, most of the subjects surveyed harbored parasite infections. Subjects infected with at least one parasite species (*p* < 0.01) are noted in Table 2. Table 2 highlights how almost all parasitized individuals harbored coinfections. (*p* < 0.01).

Although the parasite species compositions are very similar in the four areas, small quantitative differences can be observed. The prevalence of *Blastocystis* sp. turned out to be very high in the four areas.

Among protozoans, *E. coli* proved to be the most prevalent species in the Bolivian Altiplano, Cajamarca valley and the Nile Delta. In the Peruvian Altiplano, *E. coli* and *E. hartmanni* were the most prevalent protozoan species, with similar infection rates. In the Bolivian Altiplano, *B. coli* showed the highest prevalence. It should be highlighted that the protozoan species *G. intestinalis*, of well-known pathogenicity, appears among the most prevalent species in the four areas.

Among helminths, fascioliasis was the most prevalent in the four areas. Statistically significant differences are detected in fascioliasis prevalence when comparing the four areas. In these areas, (i) the overall prevalence of fascioliasis and (ii) the percentage of *Fasciola*-infected individuals showing no coinfection with other parasite species, were as follows: Bolivian Altiplano: 16.8 and 3.5%; Peruvian Altiplano: 24.6 and 0%; Cajamarca valley: 21.4 and 1.8%; Nile Delta: 13.0 and 15.9%.

The very low percentage of *Fasciola*-infected subjects presenting no coinfection with other parasites (0–15.9%, only 22 of a total of 450 *Fasciola*-infected subjects = 4.9%) is impressive (Table 2).

**Figure 2:**
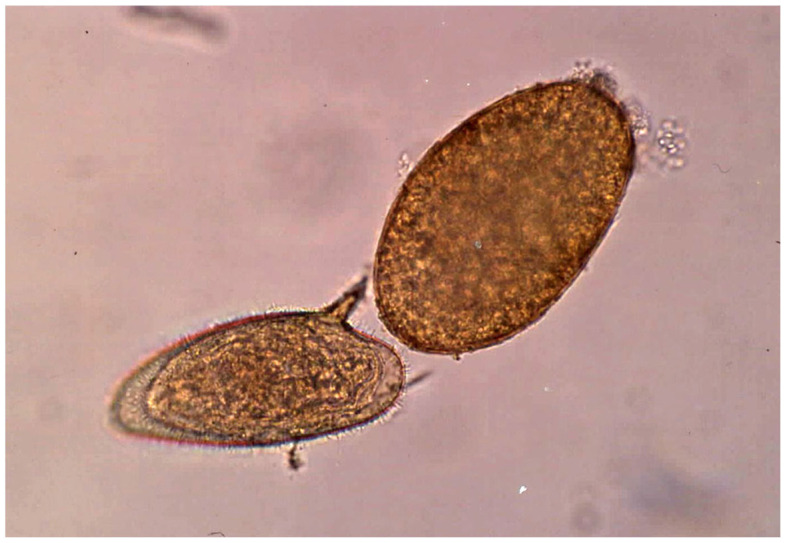
Eggs of *Schistoma mansoni* (left) and *Fasciola gigantica* (right) found in the diagnostic smear of the fecal sample of a child from the Behera Governorate, Nile Delta, Egypt. Original by S. Mas-Coma.

The most abundant helminths coinfecting *Fasciola*-infected subjects were the following: *A. lumbricoides* (10.8%) in the Bolivian Altiplano, *T. trichiura* (18.3%) in the Peruvian Altiplano, *H. nana* (15.7%) in the Cajamarca valley and *S. mansoni* (12.1%) in the Nile Delta (Figure 2).

### 3.4. Fascioliasis Analysis According to Sex and Age

Fascioliasis prevalence levels according to sex and age in the total population of the four areas are shown in Table 3.

When analyzing sex differences, it turned out that the prevalence of *Fasciola* is higher in females than in males (*p* < 0.001) in all age groups in the Egyptian population. However, in Andean countries, higher prevalences in females are not always present in each age group.

Infection by *Fasciola* flukes is present in all age groups, both in the different growth periods (childhood, adolescence) and in adults. Nevertheless, a higher percentage of cases was detected in children, i.e., in the 10–12-year age range in the Bolivian Altiplano, 7–9-year-olds in the Peruvian Altiplano, 13–15-year-olds in the Cajamarca valley, and 7–9-year-olds in the Nile Delta. However, these results must be interpreted by considering that the age ranges are not the same in the populations analyzed in the four areas, mainly regarding the adult subjects in the Nile Delta.

### 3.5. Fascioliasis and the Number of Coinfecting Parasites

Interesting results are obtained in the analysis of the number of coinfecting parasite species when comparing coinfections in *Fasciola*-infected subjects and coinfections in subjects not infected by *Fasciola*, in the total of subjects surveyed in the four endemic areas (Table 4). Multiparasitism diagnosed in the same subject included mixed infections comprising up to (i) nine coinfecting parasite species, (ii) eight simultaneous protozoan species and (iii) five coinfecting helminth species, including *Fasciola* infection.

**Figure 3:**
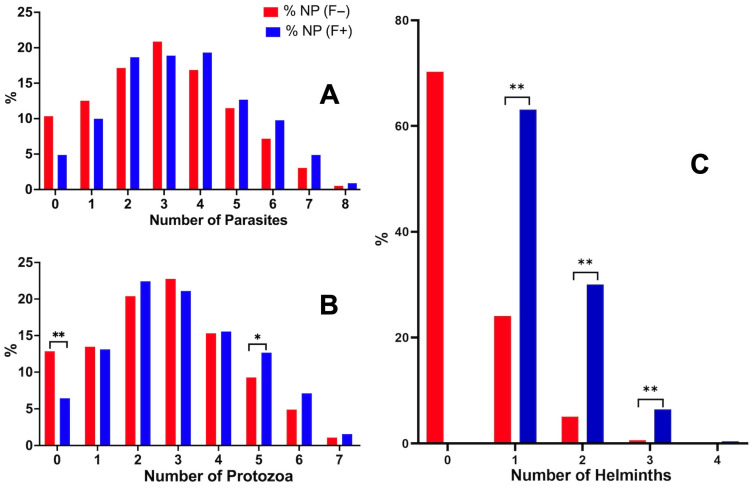
Prevalence of human fascioliasis (%), grouped by number of coinfecting parasite species (**A**), protozoan species (**B**) and helminth species (**C**) in Bolivia, Peru (Cajamarca and Puno) and Egypt. * *p*-value < 0.05. ** *p*-value < 0.001. Red %NP (F−): individuals without fascioliasis; blue %NP (F+): individuals with fascioliasis.

The comparison between the prevalence of coinfections in *Fasciola*-infected subjects and in subjects not infected by *Fasciola*, by stratifying according to the number of coinfecting parasite species, did not show statistical differences (Figure 3A). However, when distinguishing coinfections by protozoans on the one hand and coinfections by helminths on the other hand, the significance of the results proved to differ pronouncedly (Table 4).

When restricting the analysis to the number of coinfecting protozoans, the most frequent mode was of three species harbored per person. The comparison between the proportions of coinfections in *Fasciola*-infected subjects and in subjects not infected by *Fasciola* did not show statistical differences, except for the coinfection with five protozoan species (Figure 3B). In this case, a higher number of coinfection cases was detected in individuals with fascioliasis (12.67%) than in individuals without fascioliasis (9.28%) (see Table 4).

When restricting the analysis to the number of coinfecting helminths, the most frequent mode was of one coinfecting helminth species (Table 4). The comparison between the proportions of coinfections by other helminth species in *Fasciola*-infected subjects and in subjects not infected by *Fasciola* showed high statistical differences in coinfections with one, two and three helminth species, and also even four helminth species, the last case lacking significance due to the reduced number of subjects presenting such an extreme mode of multiparasitism (Figure 3C).

### 3.6. Analysis of Fasciola Infection Intensity, Age and Coinfection

Significant negative correlations were detected between the age (in years) of the host and the number of parasite (*p* < 0.001), protozoan (*p* < 0.001) and helminth (*p* = 0.003) species present, i.e., when the age of the subject individual increases, the number of parasite species detected in situations of coinfection (including protozoan and helminth species) decreases.

**Figure 4:**
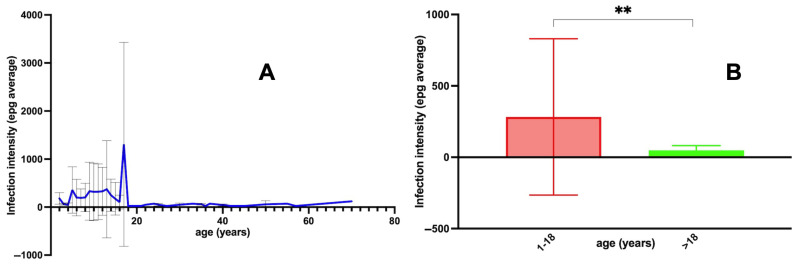
Age-associated intensity profiles of fascioliasis in Bolivia, Peru (Cajamarca and Puno) and Egypt: intensity of human fascioliasis expressed by average and standard deviation (SD) of the number of eggs per gram of feces (epg) according to: (**A**) each year of age; (**B**) 1–18 and >18 years age groups. ** *p*-value < 0.001. The bars denote SD.

In the analysis of the *Fasciola* infection intensity, a difference appears when comparing the age group of childhood and adolescents with older subjects: higher mean epg values were detected in the 1–18-year-old group than in the group made up of individuals >18 years of age (*p* = 0.003) (Figure 4).

**Figure 5:**
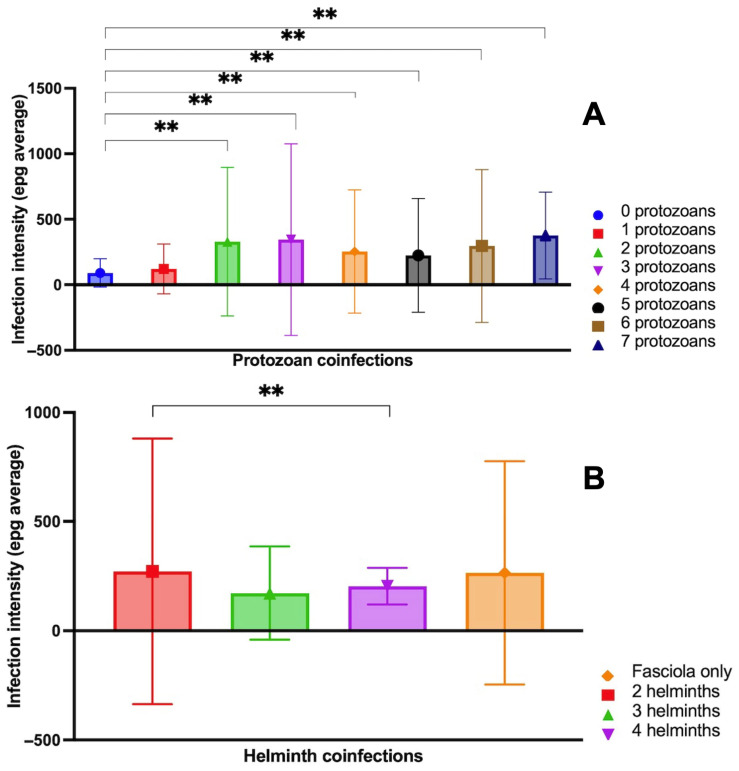
Intensity of human fascioliasis expressed by average and standard deviation (SD) of the number of eggs per gram of feces (epg), in Bolivia, Peru (Cajamarca and Puno) and Egypt: (**A**) grouped by number of protozoa species coinfection (including *Blastocystis* sp.); (**B**) grouped by number of helminth species coinfection. ** *p*-value < 0.001. The bars denote SD.

A further analysis concerned *Fasciola* infection intensity in relation to the presence or absence of coinfection. The comparison of epg levels stratified according to the numbers of coinfecting protozoan and helminth species is shown in Figure 5:

- The pairwise comparison between *Fasciola* epg with and without protozoan species coinfection showed significant differences, indicating high levels of *Fasciola* epg in situations of coinfections with two, three, four and five protozoan species vs. situations with no protozoan coinfection (Figure 5). Summing up, *Fasciola* epg values increase when the number of protozoan species present in the coinfection increases.
- The pairwise comparison between *Fasciola* epg with and without helminth species coinfection did not show significant differences, except for the comparison of the *Fasciola*-only group and the three coinfecting helminth species (including *Fasciola*) group, indicating lower values of *Fasciola* epg in situations of coinfections by three helminth species vs. situations with no helminth coinfection (Figure 5B).

### 3.7. Parasite Species Associations

Associations (analyzed with 2 × 2 tables, Fisher’s exact test) between infection by *Fasciola* flukes and infection by other parasite species simultaneously present in the same subject are shown in Table 5. Associations appear between *Fasciola* spp. and *E. coli*, *E. hartmanni, E. nana, I. buetschlii* and *G. intestinalis*, respectively. Interestingly, the above-mentioned parasite species present a prevalence above 25% in the fascioliasis-infected subjects.

### 3.8. Comparison of the Positive Rate of Coinfecting Parasites in Fasciola-Infected Individuals and Individuals Not Infected with Fasciola

Although the proportion of *Fasciola*-infected individuals presents a higher positive rate of coinfecting parasites than that found in individuals not infected with *Fasciola*, statistically significant differences were not detected in all parasite species when considering all cases.

**Figure 6:**
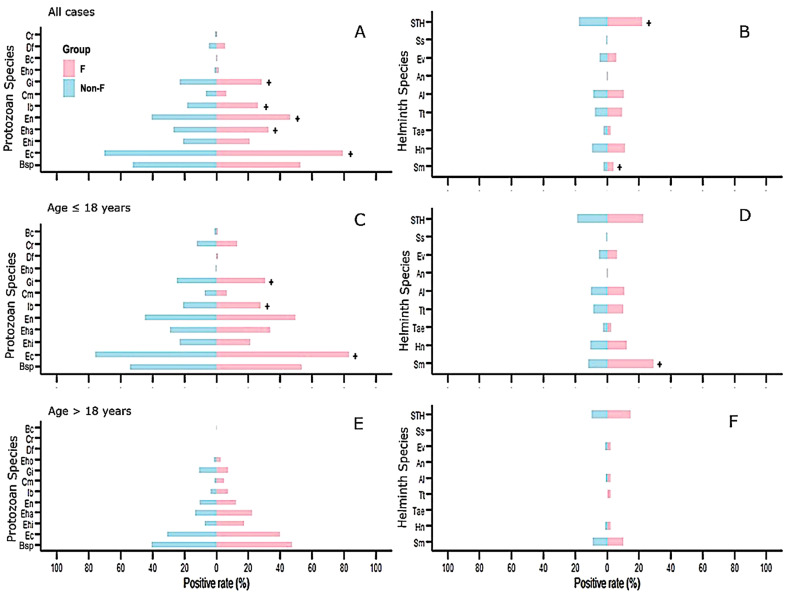
Comparison of the positive rates of 12 protozoan (including Blastocystis sp.) and 8 helminth species in *Fasciola*-infected individuals and individuals non-infected with *Fasciola*, in the four areas analyzed (Bolivian Altiplano, Peruvian Altiplano, and Cajamarca valley with only *F. hepatica*; Nile Delta with both *F. hepatica* and *F. gigantica*). The positive rate for each protozoan among 450 individuals with fascioliasis (tested for all protozoan, *Cryptosporidium* and *Dientamoeba fragilis* only detected in the Bolivian population) and among 2125 individuals non-infected with *Fasciola* (tested for all protozoans; *Cryptosporidium* and *D. fragilis* only detected in the Bolivian population) was compared in all cases (**A**) and for different age groups, comprising the population aged 1–18 years (**C**) and the population aged >18 years (**E**). The positive rates of *Cryptosporidium* and *D. fragilis* among 201 individuals with fascioliasis and among 994 non-fascioliasis individuals was compared in all cases and for different age groups (population aged 1–18 years, *n* = 201; and population aged >18 years, *n* = 0). The positive rate of each helminth among 450 individuals with fascioliasis (tested for all helminth species, *Schistosoma mansoni* only present in Egypt) and among 2125 non-fascioliasis individuals (tested for all helminth species, *S. mansoni* only detected in Egypt) was compared in all cases (**B**) and for different age groups, comprising the population aged 1–18 years (**D**) and the population aged >18 years (**F**). The positive rate of *Schistosoma mansoni* among 88 individuals with fascioliasis and among 491 non-fascioliasis individuals was compared in all cases and for different age groups (population aged 1–18 years, *n* = 371, and population aged >18 years, *n* = 308). The length of the red bar indicates the positive rate for individuals with fascioliasis and the length of the blue bar indicates the positive rate for individuals without *Fasciola* infection. The positive rate of each protozoan and helminth was compared for different age groups. The positive rate in the fascioliasis group was calculated by taking the positive number of each parasite as the numerator and the number of fascioliasis cases as the denominator. The positive rate in the non-fascioliasis group was calculated by taking the positive number of each parasite as the numerator and the number of non-fascioliasis cases as denominator. The significant differences in the positive rate (chi-square test or Fisher’s exact test) are indicated (†). Bsp = *Blastocystis* sp. Ec *= Entamoeba coli.* Ehi = *Entamoeba histolytica* complex. Eha = *Entamoeba hartmanni*. En = *Endolimax nana*. Ib = *Iodamoeba buetschlii*. Cm = *Chilomastix mesnili*. Gi = *Giardia intestinalis*. Eho = *Enteromonas hominis*. Df = *Dientamoeba fragilis* *. Cr = *Cryptosporidium* sp. *. Bc = *Balantidium coli*. STH = soil-transmitted helminths. Sm = *Schistosoma mansoni* **. Hn = *Hymenolepis nana*. Tae = *Taenia* sp. Tt = *Trichuris trichiura*. Al = *Ascaris lumbricoides*. An = *Ancylostoma duodenale* and/or *Necator americanus*. Ss = *Strongyloides stercolaris*. Ev = *Enterobius vermicularis*. * Only present in Bolivian Altiplano. ** Only present in Nile Delta, Egypt. † *p*-value < 0·05.

In relation to protozoan species (Figure 6A, Table 5), *E. coli* had the highest rate of positivity in *Fasciola*-infected individuals vs. individuals not infected with *Fasciola*, followed by *E. nana, E. hartmanni, G. intestinalis* and *I. buetchlii*. For helminths (Figure 6B, Table 5), STH presented the highest positivity rate in *Fasciola*-infected individuals vs. individuals not infected with *Fasciola*, followed by *S. mansoni*. The ranking of the most prevalent parasites was similar in protozoan species when the younger group (<18-years) was considered independently (Figure 6C,D and Table 5): *E. coli* had the highest rate of positivity in *Fasciola*-infected individuals vs. individuals not infected with *Fasciola*, followed by *G. intestinalis* and *I. buetchlii*.

Among helminths, *S. mansoni* had the highest rate of positivity in *Fasciola*-infected individuals in the case of Egypt vs. individuals not infected with *Fasciola* and STH.

The ranking of the most prevalent parasite species was similar, although no significant differences were detected between *Fasciola*-infected individuals and individuals not infected with *Fasciola* when the older group (>18-years) was considered independently (Figure 6E,F): *Blastocystis* sp. presents the highest positivity rate in *Fasciola*-infected individuals vs. individuals not infected with *Fasciola*, followed by *E. coli*, *E. hartmanni*, *E. nana* and *G. intestinalis*. Furthermore, the younger group presents higher positive rates in each one of the parasite species studied when compared to the adult group.

**Figure 7:**
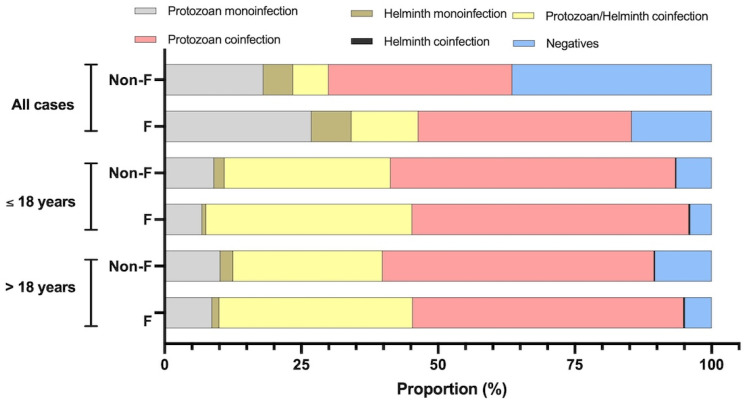
Prevalence of parasites in monoinfection and coinfection in patients with fascioliasis (F) and without *Fasciola* infection (non-F) in the four areas analyzed together (Bolivian Altiplano, Peruvian Altiplano, and Peruvian Cajamarca valley with only *F. hepatica*; Nile Delta, Egypt, with both *F. hepatica* and *F. gigantica*) (2011–2023). Positive proportion of protozoan (including *Blastocystis* sp.) monoinfection, helminth monoinfection, protozoan–helminth coinfections, protozoan–protozoan coinfections, helminth–helminth coinfections, and negative cases in all cases and in different age groups (population aged 1–18 years and population aged >18 years).

The cases of monoinfection and coinfection with protozoans/helminths in *Fasciola*-infected individuals and individuals not infected with *Fasciola* are represented in Figure 7 and Table 6. A similar pattern was detected in the *Fasciola*-infected and *Fasciola*-non-infected groups, i.e., higher rates were observed in the older group compared to the younger group only in monoinfections caused by protist/protozoan parasites, monoinfections caused by helminth parasites, and negative cases. In all cases of coinfection, the higher rate was present in the younger group compared to the older group. Concretely, considering fascioliasis cases, 8.66% had monoinfection by protozoan parasites, 1.33% had monoinfection by helminth parasites, 35.33% had coinfection by protozoan–helminth parasites, 49.55% were coinfected by protozoan–protozoan, 0.22% had a helminth–helminth coinfection and 4.88% were negative. Considering individuals not infected with *Fasciola*, 10.20% had a protozoan monoinfection, 2.4% had a helminth monoinfection, 27.3% had a protozoan–helminth coinfection, 49.7% had a protozoan–protozoan coinfection, 0.1% had a helminth–helminth coinfection, and 10.4% were negative.

Significantly higher positive rates in coinfection with protozoan–helminth parasites (*p* = 0.0009) were in the fascioliasis group vs. the non-fascioliasis group. Significantly higher positive rates of negative cases were in non-fascioliasis vs. the fascioliasis group (*p* = 0.0002).

**Figure 8:**
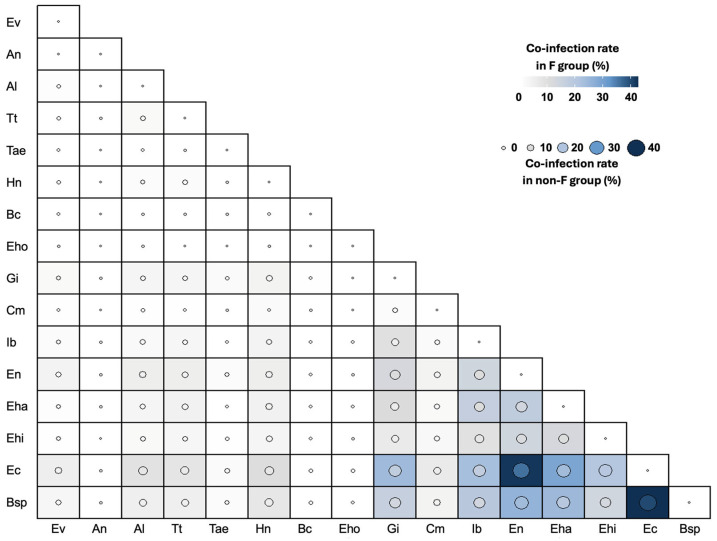
Prevalence of parasites in coinfection in *Fasciola*-infected individuals (F) and individuals not infected with *Fasciola* (non-F) in the four areas analyzed together (Bolivian Altiplano, Peruvian Altiplano, and Cajamarca valley with only *F. hepatica*; Nile Delta presenting both *F. hepatica* and *F. gigantica*. Heatmap of the coinfection rate of intestinal parasites. Grid color represents the coinfection rate of intestinal parasites among F. Dot color represents the coinfection rate of intestinal parasites among non-F. The bigger size and darker color of the circles indicate higher coinfection rates between the pair of parasite species. Bsp = *Blastocystis* sp. Ec *= Entamoeba coli.* Ehi *= Entamoeba histolytica* complex. Eha = *Entamoeba hartmanni*. En = *Endolimax nana*. Ib = *Iodamoeba buetschlii*. Cm = *Chilomastix mesnili*. Gi = *Giardia intestinalis*. Eh = *Enteromonas hominis*. Bc = *Balantidium coli*. Hn = *Hymenolepis nana*. Tae = *Taenia* sp. Tt = *Trichuris trichiura*. Al = *Ascaris lumbricoides*. An = *Ancylostoma duodenale* and/or *Necator americanus*. Ev = *Enterobius vermicularis*. Only species present in the four areas are analyzed.

Considering the *Fasciola*-infected individuals, the most common protozoan–protozoan coinfections were *Blastocystis* sp.-*E. coli* (42.66%), *E. coli*-*E. nana* (42.00%) and *E. coli-E. hartmanni* (28.88%) (Figure 8). Considering the individuals not infected with *Fasciola*, the most common protozoan–protozoan coinfections were *Blastocystis* sp.-*E. coli* (39.95%), *E. coli-E. nana* (35.85%) and *Blastocystis* sp.-*E. nana* (23.62%). The rates were significantly higher among individuals with fascioliasis than among those without fascioliasis in *E. coli-E. nana* (*p* = 0.015) and *E. coli-E. hartmanni* (*p* = 0.015). Considering the four regions independently, top-ranking coinfections were *Blastocystis* sp.-*E. coli* in Puno, Cajamarca and the Nile Delta and *E. coli-E. nana* in the Bolivian Altiplano. Among cases with fascioliasis, the most common protozoan–helminth coinfections (Figure 8) were *E. coli-H. nana* (10.22%), *E. coli-A. lumbricoides* (8.66%) and *E. coli-T. trichiura* (7.77%). Among cases without fascioliasis, coinfections included *E. coli-A. lumbricoides* (7.62%), *E. coli-H. nana* (7.48%), and *E. coli-T. trichiura* (6.40%). No significant differences were detected between these coinfection rates between individuals with and without fascioliasis.

Considering the four regions analyzed (see figure in Supplementary Materials File S2), the top-ranking coinfections in individuals with fascioliasis were similar to the non-fascioliasis group in the Bolivian Altiplano (*E. coli-A. lumbricoides*), Cajamarca (*E. coli-H. nana*) and the Nile Delta (*E. coli-S. mansoni*). In Puno, the highest coinfection was *E. coli-T. trichiura* in the *Fasciola*-infected group and *E. hartmanni-H. nana* in the *Fasciola*-non-infected group. Furthermore, *Blastocystis* sp.-*S. mansoni* appeared with a rate similar to *E. coli-S. mansoni* in the non-fascioliasis group in the Nile Delta. The rates were significantly higher among individuals with fascioliasis than among those without fascioliasis in *E. coli-S. mansoni* (*p* = 0.0004).

The most common helminth–helminth coinfections (Figure 8), considering the cases with fascioliasis, were *A. lumbricoides-T. trichiura* (2.00%), *T. trichiura-H. nana* (1.33%), and *A. lumbricoides-H. nana* (1.11%), whereas in individuals without fascioliasis coinfections included *A. lumbricoides-T. trichiura* (1.88%), *T. trichiura-H. nana* (1.51%), and *A. lumbricoides-H. nana* (0.89%). No significant differences were detected among these coinfection rates between individuals with fascioliasis and individuals without fascioliasis.

**Figure 9:**
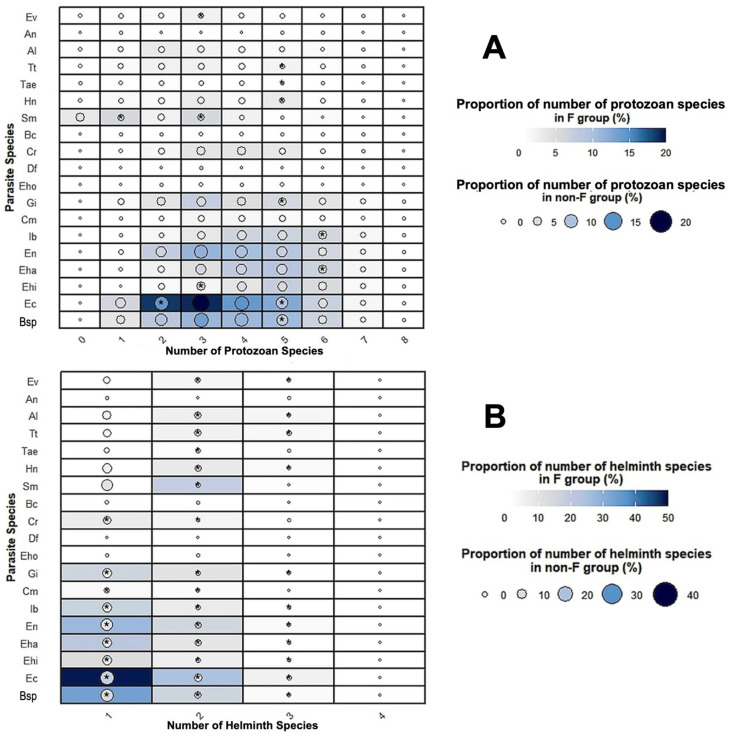
Prevalence of parasites in coinfection in *Fasciola*-infected individuals (F) and individuals not infected with *Fasciola* (non-F) in the four areas analyzed together (Bolivian Altiplano, Peruvian Altiplano, and Cajamarca valley with only *F. hepatica*; Nile Delta presenting both *F. hepatica* and *F. gigantica*). Heatmap of the coinfection rate of intestinal parasites: (**A**) vs. protozoan * number (including *Blastocystis* sp.); (**B**) vs. helminth number. Grid color represents the coinfection rate of intestinal parasites among F. Dot color represents the coinfection rate of intestinal parasites among non-F. The bigger size and darker color of the circles indicate higher coinfection rates between the pair of parasite species. Bsp *= Blastocystis* sp. Ec *= Entamoeba coli.* Ehi *= Entamoeba histolytica* complex. Eha = *Entamoeba hartmanni*. En = *Endolimax nana*. Ib = *Iodamoeba buetschlii*. Cm = *Chilomastix mesnili*. Gi = *Giardia intestinalis*. Eho = *Enteromonas hominis*. Df = *Dientamoeba fragilis*. Cr = *Cryptosporidium* sp. Bc = *Balantidium coli*. Sm = *Schistosoma mansoni*. Hn = *Hymenolepis nana*. Tae = *Taenia* sp. Tt = *Trichuris trichiura*. Al = *Ascaris lumbricoides*. An = *Ancylostoma duodenale* and/or *Necator americanus*. Ev = *Enterobius vermicularis*. * Including *Blastocystis* sp.

Considering all parasite species, the most common protozoan species was *E. coli* regardless of the number of protozoan species present in coinfection (1, 2, 3, 4, 5, 6 and 7 different protozoan species) in both groups (with and without fascioliasis) (Figure 9). The rates were significantly higher among individuals with fascioliasis than among those without fascioliasis in *E. coli, Blastocystis* sp. and *G. intestinalis* in the presence of five protozoan species and *E. hartmanni* and *I. buetchlii* in the presence of six protozoan species. The rate was significantly higher among individuals without fascioliasis than among those with fascioliasis in *E. histolytica* complex in the presence of three protozoan species. The most common helminth species vary according to the number of protozoan species present in the coinfections (1, 2, 3, 4, 5, 6 and 7 different protozoan species) in both groups (with and without fascioliasis) (Figure 9). The rates were significantly higher among individuals with fascioliasis than among those without fascioliasis in *S. mansoni* in the presence of one protozoan species, *S. mansoni* and *E. vermicularis* in the presence of three protozoan species, in *S. mansoni* in the presence of four protozoan species and in *H. nana, Taenia* sp. and *T. trichiura* in the presence of five protozoan species (Figure 9).

Considering all parasite species, the most common protozoan species was *E. coli* in the cases of one, two, three and four different helminth species present in the coinfection, in both groups (with and without fascioliasis) (Figure 9). The rates were significantly higher among individuals with fascioliasis than among those without fascioliasis in all species of the protozoan group, except for low-prevalence species (Figure 9). The most common helminth species vary according to the cases of one, two, three and four different helminth species present in coinfections, in both groups (with and without fascioliasis). The rates were significantly higher among individuals with fascioliasis than among those without fascioliasis in all species of the helminth group, except for low-prevalence species (Figure 9).

### 3.9. Multivariate Logistic Regression Analysis

The results of the multivariate logistic regression analysis are shown in Table 7 and in the figure in Supplementary Materials File S3:

- Model 1 included the numbers of the different protozoan and helminth species in each host individual as independent variables (Table 7). In this model, the risk of fascioliasis increased when the total number of helminth species per host increased [OR: 7.51 (95%CI 6.25, 9.03), *p* < 0.001]. Nevertheless, the fascioliasis risk did not increase when the total number of protozoan species was considered [OR: 1.03 (0.96, 1.11), *p* = 0.37].
- Model 2 included the population characteristics (geographical location, sex and age) and number of protozoan and helminth species as independent variables (Table 7). In this model, the risk of fascioliasis associated with the helminth number increased [OR: 7.78 (6.50–9.46), *p* < 0.001].
- Model 3 included the population age and number of cases of STH as independent variables (Table 7). In this model, the risk of fascioliasis is associated with STH cases [OR: 1.31 (1.01, 1.67), *p* = 0.03].
- Model 4 included the population age, in addition to the number of cases of *E. coli*, of *E. hartmanni*, of *E. nana*, of *I. buetschlii*, of *G. intestinalis* (in which association with *Fasciola* was detected, Table 5), and of STH as independent variables (Table 7). In this model, the risk of fascioliasis is associated with the number of cases of *E. coli* [OR: 1.37 (1.04, 1.80), *p* < 0.02], of *I. buetschlii* [OR: 1.32 (1.02, 1.72), *p* < 0.03], of *G. intestinalis* [OR: 1.26 (1.00, 1.59), *p* < 0.04] and of STH [OR: 1.28 (1.00, 1.65), *p* < 0.04], indicating that these parasitic species also partly explain this process.

Summing up, a high risk of coinfection with helminths may be expected in human subjects inhabiting fascioliasis hyperendemic areas. The results of the logistic regression analysis support that coinfection is influenced by host age, the number of helminths present and the types of helminth and protozoan species participating in coinfection.

## 4. Discussion

### 4.1. Species Compositions, Environmental and Social Factors and Infection Sources

Fascioliasis transmission is mainly based on ingestion of metacercariae attached to aquatic vegetables [84] but also through drinking water contaminated with floating metacercariae [84,126]. Aspects linked to human infection risk have already been quantified in the Bolivian Altiplano [85], including water, food, housing, behavioral traditions, social aspects and livestock management.

The maximum multiparasitisms within each endemic area were as follows:

- In the Bolivian Altiplano [72,73]: one case with *F. hepatica +* two protozoans (*E. histolytica* complex and *G. intestinalis)* + two helminths *(H. nana* and *T. trichiura*); another case with *F. hepatica +* three protozoans (*E. histolytica* complex, *G. intestinalis* and *Cryptosporidium* sp.) + one helminth (*E. vermicularis*).
- In the Peruvian Altiplano [83]: 61% of the *F. hepatica*-infected cases co-infected with pathogenic species such as two protozoans (*E. histolytica* complex and *G. intestinalis*) and five–six helminths (*H. nana*, *Taenia* spp., *T. trichiura, A. lumbricoides* and *A. duodenale/N. americanus*).
- In the Peruvian Cajamarca valley [89]: one case with *F. hepatica +* six protozoans (*E. coli, E. histolytica* complex, *E. hartmanni*, *E. nana*, *Ch. mesnili* and *Blastocystis* sp.) + three helminths *(T. trichiura, A. lumbricoides* and *E. vermicularis*).
- In the Egyptian Nile Delta [111]: one case with *Fasciola* spp. + six protozoans (*E. coli, E. histolytica* complex, *E. nana*, *G. intestinalis*, *Ch. mesnili* and *Blastocystis* sp.) + two helminths (*S. mansoni*, and *H. nana*).

### 4.2. The Sex Factor

In each endemic area, the population risk profile for fascioliasis differs by sex, probably depending on the social roles and activities of women in all age groups or sex differences in childhood in Andean populations. However, not only cultural differences in behavior associated with sex, but also sex-associated hormones may modulate immune responses and parasitic infection, as is presently known at least in protozoan infections [127].

In Egypt, fascioliasis prevalence in women proved to be higher than in men in all age groups (Table 3), which agrees with previous assessments [111,128]. The role of women in Egypt is associated with cultural, hygienic and behavioral factors [115,128]. In Egypt, women are the ones who mostly carry out activities that favor fascioliasis transmission, e.g., contact with contaminated freshwater, with the presence of vector snails and floating metacercariae, when washing clothes and kitchen utensils; in large canals, performing agricultural tasks related to irrigation such as working in rice fields; as well as with the preparation of meals and the handling of plants contaminated with metacercariae [111].

However, in the three endemic areas analyzed in Bolivia and Peru, a higher prevalence of fascioliasis in females is only detected in girls under seven years of age (Table 3). Interestingly, this is contrary to what is globally found in children under 4 years of age [129], which indicates a behavior/activity change when entering school age in rural areas (specifically, increased exposure to risky vegetables in the kitchen according to Andean ethnic traditions in rural communities, as well as to environments contaminated with *Fasciola* when walking from home to the school and back [85]).

No significant differences according to sex were found in previous surveys in the Northern Bolivian Altiplano [74], the Peruvian Altiplano [83] or the Cajamarca valley [89]. Interestingly, however, intensities (epg) showed an opposite picture: in the Bolivian Altiplano, significantly higher *F. hepatica* egg counts were found in girls [74], in the Peruvian Altiplano the highest overall egg counts were detected in girls [83], and in the Cajamarca valley females included a greater number of subjects shedding more epg [89].

In the Nile Delta, fascioliasis intensities higher than 400 epg (between 408 and 2304 epg) recently found in individuals have proved to be the highest hitherto recorded in Egypt and also throughout the Old World, and the highest epg were also found in females (60.4%) [113].

### 4.3. Endemic Area Characteristics and Infection Sources

Fascioliasis prevalence differs according to geographical location. The statistically significant differences in prevalence in the four areas analyzed are probably due to factors such as hygiene, climate and altitude, as well as cultural or religious features.

No basic qualitative differences were found in the species spectra of protozoans and helminths in the four hyperendemic areas (Table 2). The species *Blastocystis* sp. and *E. coli*, *E. histolytica* complex, *E. hartmanni*, *E. nana*, *I. buetschlii*, *Ch. mesnili*, *G. intestinalis*, *E. hominis*, *D. fragilis*, *Cryptosporidium* sp. and *B. coli* are known to follow a monoxenous life cycle with fecal–oral transmission, including contaminated water/food as sources of infection. These protozoan species can be considered as indicators of the high levels of fecal environmental contamination, both of animal and human origin. The presence of *Cryptosporidium* sp. only in the Bolivian Altiplano and the higher prevalence of *B. coli*, also in the Bolivian Altiplano, suggest close relationships with infected cattle and pigs, respectively [72,73].

Concerning helminths, the homogeneity of the worm species spectrum is also notable. The nematode species *T. trichiura* and *A. lumbricoides* also follow a monoxenous cycle. The infection is caused by the ingestion of the helminth eggs present in contaminated water or food or from soil-contaminated hands. Consequently, these helminths are also indicators of the high levels of fecal contamination in the environment.

The cestode species *H. nana*, in spite of its typical two-host life cycle, transmitted by an insect harboring the infective larval stage of cysticercoid (usually a stored-flour-contaminating beetle) [130], should also be included as such a fecal indicator, because of its capacity to evolve monoxenously after ingestion of eggs dispersed throughout the external environment. Indeed, *H. nana* eggs are able to release their hexacanth embryo in the human intestine, subsequently penetrate the villi to evolve until the cysticercoid stage and finally return to the intestinal lumen to develop the adult stage.

*Taenia* species follow a life cycle, with oral infection by ingestion of raw infected pork or beef [130]. Taeniasis in Andean countries and its absence in Egypt appears to be related to cultural, dietary and religious habits (absence of pork in the Muslim diet).

The prevalences of trichuriasis and ascariasis in Andean countries do not differ between the two Altiplanos and the Cajamarca valley despite the lower altitudes of the latter, although prevalences of geo- and pseudogeohelminths are known to normally decrease with increasing altitudes [131]. Interestingly, moreover, trichuriasis and ascariasis also show lower rates in the Nile Delta human fascioliasis endemic area.

The hookworms *A. duodenale* and *N. americanus* and the rhabditid *S. stercoralis* follow a one-host life cycle with transcutaneous infection by an infective larval stage, although oral infection of humans by *S. stercoralis* is also known to occur [132]. Human infection by hookworms and *S. stercoralis* are primarily acquired by walking barefoot on contaminated soil and are, therefore, indicators of the high human fecal contamination of the surrounding land. Hookworm infection has been detected in the Bolivian and Peruvian Altiplanos and in the Nile Delta, but not in the Cajamarca valley. The presence of *S. stercoralis* was only found in the Peruvian Altiplano (Table 2). Soil-transmitted helminth (STH) infections are among the most common infections worldwide and affect the poorest and most deprived communities [130]. Risk factors for transmission of STH infections are poor sanitary conditions, inadequate water supply, overcrowding, low socioeconomic status, living in proximity to animals and rural areas as opposed to urban areas [133].

The two-host life cycle *of S. mansoni* should be highlighted, being an exception due to its transcutaneous infection by infective furcocercariae shed by freshwater planorbid snails. Thus, the transmission is not linked to oral infection, but to agricultural, domestic, occupational and recreational activities which expose the naked skin of humans to infested water [130], although ingestion of cercariae when drinking natural water cannot be disregarded. The freshwater milieu and the usual coexistence of fascioliasis lymnaeid vectors and schistosomiasis planorbid vectors in the same freshwater bodies appear as the common epidemiological factors underlying such human coinfections in the Nile Delta.

Summing up, the four rural fascioliasis endemic areas in question show similar qualitative compositions of parasites, suggesting similar hygienic–sanitary and socio-economic conditions, i.e., poverty, poor hygiene, a lack of clean water and adequate sanitation, as well as difficulties in the access to preventive measures and efficacious treatment [134]. However, the prevalence rate of the most frequent parasite species in each area analyzed is different, depending on whether they are protozoans or helminths. *Blastocystis* sp. is present with a prevalence above 40% in the four areas. The non-pathogenic *E. coli* is the most common species in the four areas. However, the most abundant helminth species after *Fasciola* spp. proves to be different in the four areas, evidencing the influence of environmental conditions: *A. lumbricoides* in the Bolivian Altiplano, *T. trichiura* in the Peruvian Altiplano, *H. nana* in the Cajamarca valley and *S. mansoni* in the Nile Delta.

### 4.4. Parasite Associations and Infection Sources

The parasite transmission routes and human infection sources underlie the associations of fascioliasis with infections by protozoans and helminths. Our analysis has detected associations between fascioliasis and *E. coli*, *E. hartmanni*, *E. nana*, *I. buetschlii* and *G. intestinalis*, as well as with *S. mansoni*, *H. nana*, *Taenia* spp. and STH (when *A. lumbricoides* and *T. trichiura* are considered together) (Table 6).

A few previous studies have already reported some of the fascioliasis associations highlighted in our coinfection analysis, as, for instance, between *Fasciola* and *G. intestinalis* in the Bolivian Altiplano [72,73] and in the Peruvian Altiplano [83], and between fascioliasis and *E. coli*, *Ch. mesnili* and *E. hartmanni* in the Cajamarca valley [89]. In the Atlixco area, Puebla State, Mexico (catalogued as a mesoendemic human fascioliasis area, with hyperendemic local situations), fascioliasis proved to be associated with the presence of *E. histolytica* complex, *G. intestinalis* and *Blastocystis* sp. [62].

The coinfection of *Fasciola*/*G. intestinalis*, highlighted in the three Andean endemic areas, also agrees with the high infection rates of *G. intestinalis* found in the area of the Nile Delta [111]. These results (Table 2) support the use of this protozoan species as a biomarker indicating a high risk of fasciolid infection by freshwater drinking.

Interestingly, in the Peruvian Altiplano, the typical aquatic vegetation is absent in the lymnaeid-snail-inhabited small drainage channels linked to the local irrigation system where children play, whereas aquatic plants are present in the main irrigation canals which conduct water permanently. The small lateral canals are only flooded when needed, which is insufficient to enable the aquatic plants to grow. Quechua inhabitants of this irrigation zone mentioned that aquatic plants are not included in their diet, contrary to Bolivian Aymaras of the Northern Bolivian Altiplano. The few vegetables produced and consumed by the inhabitants of the Altiplano are tubers, legumes or cereals, the edible parts of which are not contaminated by irrigation water [84,85]. The fact that the diets of these Andean communities do not include aquatic vegetables has already been emphasized [135], with the rare exceptions concerning traditional dishes (llaytha or cochayuyo) and beverages made of local plants (e.g., jugo de alfalfa) [84,85]. Because of the absence of piped drinking water in their dwellings, local inhabitants of these rural communities obtain water from irrigation canals and drainage channels for drinking, cooking, personal hygiene, cleaning and washing. This suggests floating metacercariae in natural water as infection source [84,126].

Among associations of *Fasciola* spp. with infection by other helminth species, *A. lumbricoides* and *H. nana* should be highlighted (Table 2 and Table 6). The association between *Fasciola* and *A. lumbricoides* was already detected in the Cajamarca valley [90]. In the Atlixco area, Puebla State, Mexico, the association between *Fasciola* and *H. nana* added to that with *A. lumbricoides* [62]. Curiously, no association of *Fasciola* infection with infection by *T. trichiura* infection was found.

The association between *Fasciola* and *S. mansoni* in the Nile Delta was already highlighted [111]. This particular association has been justified because of the similar transmission routes involving common freshwater bodies and freshwater snails of similar ecology, despite the different infection pathways (oral/transcutaneous) of the two diseases.

The absence of associations between *Fasciola* infection and infection by other parasite species that also share the same infection route, as is the case of *T. trichiura* (when considered individually instead of within STH) or other monoxenous protozoan species, as well as the association of fascioliasis with *Taenia* spp., pose questions about the potential complexity of the underlying processes, including differences according to the endemic areas, e.g., the absence of porcine meat ingestion in the Nile Delta according to religious traditions, whereas extensive pig populations can be found in the rural endemic areas of Bolivia and Peru [5]. In the case of STH, although all these nematodes enter the body through the mouth, their transmission by soil and dust and the involvement of dirty hands pronouncedly differs from fascioliasis transmission.

### 4.5. Fascioliasis Immune Response

Increasing evidence suggests that exposure to fascioliasis compromises the host individual’s ability to resist other infectious diseases [69,136].

The various mammal-infecting stages of the liver fluke contribute to the heterogeneity in immune responses. Juvenile migrating flukes, adult flukes in the bile duct and eggs each elicit stage-specific immune responses [137,138], which change over time. Along the initial phases of infection (tissue or bile duct habitat), the liver fluke induces a potent systemic Th2/Treg polarized immune response simultaneously with a suppression of Th1/Th17 cytokines [136,137,138,139,140,141,142,143,144,145,146,147,148,149,150]. During the initial and the chronic phase of the infection, *F. hepatica* is associated with the expansion or recruitment of Foxp3 (the forkhead box transcription factor, a type of Treg cells) and the induction of adaptive Ag-specific Treg cells [22,143].

In human fascioliasis, in the acute phase, a predominant Th2 response takes over, with production of IL-4, IL-5, as well as mastocytosis, hypereosinophilia and IgE production and the absence of IFN-gamma and IL-2. Increases in IL-5 and IL-17 during human acute fascioliasis have been demonstrated when compared to the levels of these cytokines in controls and humans with chronic infections [151]. Furthermore, the chronic phase is characterized by a modulation of the immune response, so that eosinophilia largely resolves and cytokine levels are suppressed [151].

Many parasites stimulate a polarized immune response that involves the development of T helper cells with characteristic Th1/Th17 or Th2/Treg cytokine profiles, the direction of polarization often depending on the type of infection [152]. In coinfections, the immune response is variable, and the direction of polarization depends on the combination of infections and their different interactions with the host’s immune system [34].

Several studies have recognized potential functions for the interaction between helminths and the host’s microbiome in the pathophysiology of helminthic diseases and in the suppression of the host’s inflammation mediated by parasites [153].

However, the outcome of infections in multiparasitism depends on the means and potency by which each pathogen controls the immune response. The intracellular protozoan *Toxoplasma gondii* suppresses the recruitment and alternative activation of macrophages, which are normally associated with helminth infection, and, consequently, the Th2 responses produced in a pre-established infection by *F. hepatica*. The production of *T. gondii*-specific Th1 cytokines is not suppressed by *F. hepatica* infection, but the *T. gondii* infection inhibits the ability of splenocytes from *Fasciola*-infected mice to produce Th2 cytokines [64].

### 4.6. Fascioliasis Risk Increases as the Number of Helminth Species Increases

Immunomodulation in helminth infections shows two common modes of action [154]: (i) certain groups of proteins play key roles in bringing about changes in the host immune response; and (ii) T regulatory (Treg) cells and the induction of IL-10 play central roles in helminth-induced host immune modulation, by means of classical Foxp3+ CD4 cells or IL-10-secreting populations of CD4+ or CD8+ cells.

Helminth infections, including fascioliasis, are associated with a battery of immunomodulatory mechanisms that affect all facets of the host immune response to ensure their persistence within the host, enabling the host to benefit from suppression of collateral damage during infections. However, helminth infections can also have detrimental effects which increase the susceptibility to coinfection [154,155]. These two common modes of immunomodulation in helminths could explain the reason why the risk of fascioliasis increases when the total number of helminth species per host increases, as helminths can suppress immune responses to unrelated pathogens [155]. Treg cells would induce a suppression non-specific to discrete helminths, spreading suppression to immune responses to other helminths [143,156,157,158,159,160].

Our studies reveal higher *Fasciola* epg in the 1–18-year-old group than in the group made up of individuals above 18 years of age. Prior investigations in the Bolivian and Peruvian endemic areas already underscored high *F. hepatica* epg values in children and the associations between these elevated epg levels and the symptoms and pathology observed in infected children [72,73,83]. The decrease in *Fasciola* epg when the number of helminths increases, as detected in our study, agrees with the decreasing burden obtained by other authors in several experimental definitive host models; these studies examined *Fasciola* infections followed by a *Schistosoma* spp. infection and encountered lower worm burdens (up to 92%) for the subsequent infection [161], particularly when followed by a patent *Fasciola* infection [161,162].

The correlation of protection/disease progression in fascioliasis coinfection, along with its propensity to induce strongly skewed immune responses and bystander immune regulation, is still not completely elucidated. Concretely, *F. hepatica* suppresses host innate immune responses, ensuring that host protective responses are quickly incapacitated [22,163,164]. Immune hyporesponsiveness, exhibited as a reduced ability of host lymphocytes to proliferate, has been demonstrated in the acute and advanced chronic phases, whether primo-infected or reinfected during the advanced chronic phase [22,164].

### 4.7. Association Between Fasciola spp. and E. coli, E. hartmanni, E. nana, I. buetschlii and G. intestinalis

In fascioliasis, the suppression of Th1/Th17 responses to concurrent viral, bacterial, or protozoal infection starts on the first day of infection [64,137,146,164,165,166].

The results of the logistic regression analysis in our study show that coinfection is influenced by the type of protozoan species participating in coinfection. The results obtained in our Model 4 support that *E. coli, E. nana, I. buetschlii* and *G. intestinalis* also partly explain this process.

The positive association detected between *Fasciola* spp. and *E. coli, E. hartmanni, E. nana, I. buetschlii* and *G. intestinalis* can be explained by the immunomodulation and immunosuppression of fasciolid infection. Furthermore, in the four hyperendemic areas analyzed, *Blastocystis* sp. is very frequent, although no association with fascioliasis has been detected. *Blastocystis* sp. appears to modulate the host enteric immune response by inducing the production of specific Th1/Th17 cytokines [167]. On the other hand, *G. intestinalis* is the most prevalent pathogenic protozoan. In the small intestine, especially the duodenum, trophozoites adhere to the microvilli of the epithelium, disrupting mucosal homeostasis. The early immune response is primarily Th1, including gamma interferon (IFN-gamma), TNF-alpha and IL-17, and is followed by a Th2 response [168]. In both cases, *Blastocystis* sp. and *G. intestinalis* coinfection, the polarization to Th2/Treg and immunosuppression of fasciolid infection makes host responses difficult.

The beneficial or detrimental outcome of host–microbiome coexistence depends largely on the balance between regulators and responder intestinal CD4+ T cells [169]. Data on the correlation of immunocompromised individuals with intestinal parasitic infection has been supplied by several authors. For example, a consistent association between HIV infection and intestinal parasitosis has been reported in tropical regions [170] and especially in patients with CD4+ T-cell counts below 200 cells/mm^3^ [171]. Previous studies demonstrated that the frequency rates of *E. coli* and *Blastocystis* sp. were high in patients with a low lymphocyte percentage, i.e., CD3+ T cell and CD3+ CD4+ T cell levels in blood samples [172]. *Blastocystis* sp., *G. intestinalis* and *E. coli*, among other species, were detected in immunocompromised pediatric patients with diarrhea [173,174], and IgA and CD4(+) T cells are fundamental to the process of *Giardia* clearance [175]. In this sense, *Blastocystis* sp., *E. coli* and *G. intestinalis*, with their high prevalences detected in the four populations analyzed, may be considered as biomarkers of the immunosuppression.

Several studies on the immunomodulatory fascioliasis-related effects of combinations of pathogens have focused on animal models [136,139,140,141,176,177]. For example, studies in cattle with *Mycobacterium bovis* and *F. hepatica* showed coinfection to be associated with either no effect or a decreased response to skin and IFN-gamma tests, and control lesion detection and mycobacteria cultured or recovered [136,139,140,141,146,177,178,179]. Human fascioliasis associated with the human immunodeficiency virus and active tuberculosis has been reported [180]. Furthermore, human fascioliasis and brucellosis in the same patient [65], as well as coinfection of human fascioliasis with *Klebsiella pneumoniae* (bacteremia) have been described [59]. Coinfection with *F. hepatica* may increase the risk of *Escherichia coli* O157 shedding in British cattle [181], and an association of *F. gigantica* coinfection with bovine tuberculosis has also been found [182]. Interestingly, the high risk of bacterobilia associated with advanced chronic fasciolosis in an experimental rat model is explained by the immunosuppressive stage induced by long-term fascioliasis [166].

### 4.8. Association Between Fasciola spp., STH and S. mansoni

There is a substantial literature on the epidemiological patterns of STH infections and their control. However, the significance and the mechanisms acting in coinfections with other diseases are still not well understood. As with fascioliasis, published reports show that geohelminths can profoundly modulate immune responses of hosts, generally eliciting a Th2-biased response [155,183,184]. This immune response is thought to be critical for immunomodulation in a human host, as it can suppress the type 1 helper T-cell response required to control other infections, thus increasing the likelihood of coinfection.

Helminth antigens and secreted molecules exert modulatory effects on immune cells. Gastrointestinal nematodes produce excretory–secretory products that modulate the antigen-presenting function of dendritic cells and the function of bone marrow-derived macrophages [185,186]. A study among children from an urban region of Brazil, on the effects of chronic infections with *A. lumbricoides* and *T. trichiura* (measured twice over a 5-year period) on cytokine and antibody responses, showed a Th2 immune response, with the induction of immune hyporesponsiveness associated with greater frequencies of spontaneous IL-10 production. This hyporesponsiveness occurred during current and chronic infections and during coinfections [187].

In helminth infections, Treg cells are also involved in the weakened defenses against other pathogens. In human subjects, in vitro T-cell proliferative responses to Bacille Calmette–Guerin (BCG) vaccination and malaria are attenuated in geohelminth-infected patients, but recover if Treg cells are removed from the test cultures [188]. On the other hand, BCG vaccination of helminth-infected subjects elicits poor inflammatory cytokine responses to purified protein derivative antigen, in contrast to significant TGF-beta production. Anthelmintic treatment reverses this scenario, suggesting that the interference with vaccine responses might be due to the presence of immunosuppressive cytokines [189]. A study reported that tuberculosis-infected migrants who were coinfected with helminths had higher Treg cell frequencies than those with tuberculosis alone, but that anthelmintic treatment decreased Treg cell numbers while increasing Th1 effector populations [190].

The association between schistosomes and STH has also been previously described. Empirical studies showed a high prevalence of mixed infections, as well as higher infection intensities in coinfected patients. However, these positive interactions appear to depend on the number of different helminth species present and on the intensity of infection in each individual [191,192,193,194,195,196]. On the other hand, STH infections could increase the risk of infection with concomitant malaria.

Several possible explanatory mechanisms have been evoked, mainly also based on an altered immunological response to the infection in subjects who are already infected with STH. Immunological models suggest that helminth infections are associated with chronic immune activation affecting the acquisition of immunity to malaria. However, further research is needed to elucidate the complex relationship between STH and other infections in co-endemic settings [197]. *Ascaris lumbricoides* induces a predominant Th2/Treg response which may downregulate critical Th1 responses that are crucial for tuberculosis protection in human populations [198].

Coinfection studies in schistosomiasis showed that, generally, a previous infection with *Schistosoma* species, particularly an overt infection, often influences subsequent infection with a protozoan, bacteria or other helminths [162]. Some studies other than ours also described natural *Fasciola/S. mansoni* coinfections in human populations [199,200,201,202]. Although these studies focused on different aspects of the effects of this coinfection, their findings generally indicated increased immunological and pathological responses in the coinfected host.

One study found that coinfected hosts suffered stronger periportal fibrosis as well as higher associated levels of procollagen III peptide markers than non-coinfected hosts, with the highest levels occurring in 5–14-year-old children [200]. Similarly, other studies found that coinfected patients had higher egg counts, which were accompanied by higher serum gastrin levels [199] and higher levels of free radicals associated with the inflammatory response [201]. Coinfected hosts with fascioliasis had higher IgM and lower IgG levels relative to such levels in patients only infected by *Fasciola*, and these levels were not correlated with *Fasciola* egg counts [202]. Reports indicate that *Schistosoma/Fasciola* coinfections may be common in cattle in certain parts of the world [203].

### 4.9. Fasciola Infection Intensity, Age and Coinfection

Infection patterns according to age can provide information about who is most at risk. For most helminth species, infection intensity rises dramatically with age. Nevertheless, the age of maximum intensity varies for each helminth species. Our results showed the most intense fasciolid infections in the 1–18-year-old age group, agreeing with prior analyses carried out in STHs that showed that the most intense helminth infections occur in children of school age [204,205].

Furthermore, previous studies carried out on other helminth species have shown a positive association between intensity and concurrent infection by helminth species, suggesting that individuals harboring multiple helminth species also suffer the most intense infections [191,192,206,207,208,209,210,211,212]. Our results on fasciolid infection showed different patterns depending on whether the coinfection by protozoan or helminth species was considered:

- In protozoan coinfection, a general influence was detected, showing that fascioliasis coinfection with an elevated number of protozoan species presents higher epg levels than in cases of fascioliasis monoinfection.
- In helminth coinfection, a general influence was detected in fascioliasis coinfection with an elevated number of helminth species that presents lower epg levels than in cases of fascioliasis monoinfection.

Therefore, polyparasitism may have a greater impact on morbidity than single species infections, since morbidity is typically related to infection intensity for most parasite species. Infections by multiple species may also increase susceptibility to other infections [213,214,215]. However, the health impacts of polyparasitism have not been studied sufficiently, despite their public health significance.

### 4.10. Pathology and Coinfections

The identification of human fascioliasis endemic areas in many countries has reshaped the understanding of human fascioliasis [10,29,69], showing a severe symptomatology and pathology [27,28,29,30,69]. Studies have shown this disease to be pronouncedly complex, giving rise to progressive general deterioration of the patients, with sequelae sometimes leaving subjects handicapped and frail, and sometimes even leading to fatal cases [22,25,29].

Fascioliasis pathogenicity extends beyond the acute phase to include the biliary and advanced chronic stages, especially in endemic areas [166,216,217]. Like other parasitic diseases, certain pathological aspects of fascioliasis are associated with inflammation. For instance, reinfection with *F. hepatica* during the chronic phase triggers a diverse immune response involving Th1/Th2/Th17/Treg against the parasite. The systemic immune response varies depending on the timing of infection/reinfection, with the expression levels of Th1/Th2/Th17/Treg profiles correlating with the clinical manifestations of anemia [22]. The regulatory role of Treg also involves regulating colitis [218], dampening pulmonary inflammation in *Pneumocystis* infections [219], controlling hepatic pathology in *Schistosoma* infections [220], and regulating immunopathological lesions in viral infections [221]. Furthermore, low-intensity polyparasitism can be as health-detrimental as high-intensity infection with a single helminth species. The increasing odds of morbidity increase with the severity of the polyparasitic infection profile, and the association between low-intensity multiple infection and morbidity remains significant [222,223].

Our results show associations between *Fasciola* spp. and *E. coli, E. hartmanni, E. nana, I. buetschlii, G. intestinalis*, STH and *S. mansoni*. Considering the significant immune modulations by fascioliasis and its concurrent geographical prevalence with other protozoan and helminthic infections, all in all, the scenario indicates that fascioliasis could modify the clinical phenotypes of coexisting parasitic diseases. This hypothesis gains relevance in instances of coinfection with *G. intestinalis*, STH and *S. mansoni*, given their recognized impacts on both the physiological and neurocognitive development of affected individuals [130,133,224].

Consequently, coinfections are an important factor to be considered when assessing the health impact of fascioliasis in human communities and should therefore be taken into account when calculating the disability-adjusted life year (DALY) metric for this disease. Our results agree with conclusions concerning the need to consider polyparasitism and respective co-morbidities for the DALYs calculation, as highlighted in other parasitic diseases [35]. The implementation and integration of large-scale fascioliasis-control schemes, launched within the WHO Roadmap, should therefore be complemented by other helminthiasis control schemes on the same scale.

Given that pathogens frequently involved in fascioliasis coinfection, such as *G. intestinalis*, STH and *Schistosoma* spp., all negatively impact the physical and intellectual development of the host, the analysis of the fascioliasis-related effect on malnutrition and child development becomes essential.

### 4.11. Limitations

The limitations of this study should be considered. Indeed, causal relationships cannot be established based on the positive detection of parasites, making it challenging to determine whether coinfection was a cause or consequence of *Fasciola* infection. The differences in ethnographic characteristics of the inhabitants of the four endemic areas in question do not allow for similar data collection and control strategies. As already highlighted by WHO, field studies and control interventions on human fascioliasis should adapt to the local circumstances and intervention feasibilities. When dealing with the great heterogeneity of remote, rural endemic areas such as these, there is no other way but to take into account the reality regarding the pragmatic feasibility of interventions. These are unavoidable conditions to which the sampling strategies should adapt to successfully reach the study goals. Relying solely on microscopy without molecular confirmation should be an acknowledged method in such field studies, particularly regarding species of the *Entamoeba histolytica* complex.

## 5. Conclusions

Our analysis reveals that multiple factors, including transmission ways and immunological, environmental and social aspects, influence coinfections in human fascioliasis. Coinfection frequency by protozoan or other helminth species varies throughout hyperendemic areas, with *E. coli* and *Blastocystis* sp. being the most frequent, whereas helminth coinfections depend on environmental conditions.

OR models demonstrate that: (i) fascioliasis risk increases when the total number of helminth species per host individual increases; (ii) both protozoan and helminth species involved in the coinfection influence the process; (iii) the pathogenic effect of fascioliasis is not only direct, but also includes the immune-modulatory role of the liver fluke, giving rise to a predisposition to harbor numerous helminth species; (iv) care should be taken when assessing potential interactions of *Fasciola* infection with the coexisting intestinal microbiota in inhabitants of human fascioliasis hyperendemic areas, because of the high rarity of *Fasciola*-infected subjects presenting no coinfecting helminth and/or protozoan.

Our study highlights the complex biological interactions in chronic helminth coinfections. Further research is needed to understand the combined effects and underlying mechanisms of these coinfections. Key objectives should include analyzing the impact of coinfections in human fascioliasis on infection duration, transmission risks, clinical symptoms, pathogenicity, treatment efficacy and control strategies. Factors such as the time interval between the first infection by a parasite and the second infection by another parasite, the order of infection (which was the first infecting parasite and which one the second), the coinfecting parasite species, the dose of the infectious agents, host age at the time of infection and host age at the time of the diagnosis are relevant factors to be considered.

The patterns identified in this study, while descriptive, are of broad interest and may aid in better identifying fascioliasis coinfections for optimal antiparasitic treatment and control. These findings might also contribute to improving guidelines for drug prescription in preventive chemotherapy for high-risk populations.

## Figures and Tables

**Table 1:** Characteristics of the samples studied and the endemic areas analyzed focusing on sex and age of the subjects analyzed.

Country	Bolivia (*n* = 1195)	Peru (*n* = 701)		Egypt (*n* = 679)	Total (*n* = 2575)
Geographical area	Northern Bolivian Altiplano—between Lake Titicaca and La Paz	Peruvian Altiplano—Asillo zone, Puno *n* = 339	Cajamarca valley *n* = 362	Behera Governorate, Nile Delta	
Transmission pattern	Altiplanic	Altiplanic	Valley	Eastern Mediterranean	
Altitude m a.s.l.	3820–4100 m	3910 m	2627–3061 m	Lowlands at 4–20 m	
Study population	%	%	%	%	%
Male sex	56.1%	58.4%	51.4%	33.4%	49.7%
Age (years)					
<7	9.5%	10.9%	14.4%	18.7%	12.8%
7–9	34.8%	50.1%	43.7%	7.2%	30.9%
10–12	37.1%	30.7%	30.2%	11.9%	28.6%
13–15	15.3%	8.3%	8.9%	9.8%	12.0%
16–18	3.1%	0%	1.1%	6.8%	3.4%
>18	0.2%	0%	1.7%	45.4%	12.3%
Age mean ± IQR (Range)	10.0 ± 4 (2–19)	9.0 ± 4 (5–15)	9.0 ± 4 (2–72)	16 ± 4 (1–80)	10.00 ± 5 (1–80)

*n* = total subjects analyzed. % = percentage corresponding exclusively to the subject sample studied and not to the total population from which the sample was drawn. a.s.l. = above sea level. IQR = interquartile range.

**Table 2:** Infection prevalence (%) of each parasite species in the areas surveyed.

Geographical Area	Bolivia (*n* = 1195)		Peru (*n* = 701)				Egypt (*n* = 679)		Total (*n* = 2575)	
			Puno (*n* = 339)		Cajamarca (*n* = 362)					
Species	%		%		%		%		%	
Protozoans *										
*Blastocystis* sp.	43.2		84.0		73.4		41.5		52.4	
*Entamoeba coli*	86.7		91.1		75.3		34.0		71.9	
*Entamoeba histolytica* ^a^	19.9		39.6		33.0		7.1		21.0	
*Entamoeba hartmanni*	13.3		91.1		41.2		15.5		28.1	
*Endolimax nana*	54.1		47.0		52.5		10.6		41.5	
*Iodamoeba buetschlii*	12.6		54.7		37.6		5.9		19.9	
*Chilomastix mesnili*	8.9		7.1		6.0		2.5		6.6	
*Giardia intestinalis*	23.9		30.2		27.2		19.4		24.1	
*Enteromonas hominis*	1.2		0.6		0.5		1.3		1.0	
*Dientamoeba fragilis*	0.3		0		0		0.0		0.1	
*Cryptosporidium* sp.	12.6		0		0		0		5.8	
*Balantidium coli*	1.5		0.9		0.3		0.3		0.9	
Total number of protozoa * species		12		10		10		10		12
**Helminths**										
*Fasciola* spp.	16.8 **	201	24.3	78	21.4	83	13.0	88	17.5	450
*Schistosoma mansoni*	0.0		0		0		12.1		3.2	
*Hymenolepis nana*	8.6		16.9		15.7		4.9		9.7	
*Taenia* ssp.	4.7		0.3		0.8		0.0		2.3	
*Trichuris trichiura*	8.8		18.3		7.1		1.9		8.0	
*Ascaris lumbricoides*	10.8		8.0		14.3		4.0		9.1	
Ancylostomatidae spp. ^b^	0.2		0.6		0		0.1		0.2	
*Strongyloides stercolaris* ^c^	0.0		0.3		0		0.0		0.1	
*Enterobius vermicularis* ^c^	8.5		1.5		1.8		2.8		6.0	
STH	17.15		25.36		18.23		17.37		18.44	
Total number of helminth species		7		8		6		7		8
Total number of parasite species		19		18		16		17		20
% population infected with at least one species	97.9		100		97.3		72.7			
% individuals with fascioliasis and coinfections	96.5		100		98.7		84.1			
% individuals with fascioliasis and without coinfections	3.5		0.0		1.3		15.9			

*n* = total subjects analyzed for each parasite. % percentage infected. * including *Blastocystis* sp. (SAR group). ** data from all endemic areas. ^a^ *Entamoeba histolytica* complex. ^b^ *A. duodenale* and/or *N. americanus*. ^c^ Prevalence data for *S. stercoralis* and *E. vermicularis* may be considered underestimations, as the agar plate method (for *S. stercoralis*) and anal swabs (for *E. vermicularis*), the most adequate techniques for the detection of these helminths, could unfortunately not be used due to their methodological difficulties in the context of field work.

**Table 3:** Prevalence of fasciolid infections (%) grouped by age and sex, and related differences among the 2575 study participants in Bolivia, Peru (Cajamarca and Puno) and Egypt.

			Total		Females		Males		*p*-Value *
		Age	PC	Prevalence (95% CI)	PC	Prevalence (95% CI)	PC	Prevalence (95% CI)	
Bolivia (*n* = 1195)		<7	23	9.5% (6.3, 13.7)	12	10.6% (5.8, 18.2)	11	8.5% (4.5, 15.1)	
		7–9	38	13.2% (9.6, 17.5)	22	15.5% (10.2, 22.7)	16	10.9% (6.6, 17.4)	
		10–12	97	21.9% (18.3, 25.9)	44	22.2% (16.8, 28.8)	53	21.7% (16.8, 27.5)	
		13–15	37	20.2% (14.9, 26.5)	7	10.9% (4.9, 21.8)	30	25.2% (17.9, 34.1)	
		16–18	6	16.2 % (6.8, 30.7)	2	25.0% (4.4, 64.4)	4	13.8% (4.5, 32.6)	
		>18	-	-	-	-	-	-	
	Total		201	16.8% (14.8, 19.0)	87	16.6% (13.5, 20.1)	114	17.0% (14.3, 20.1)	
	*p*-value *			<0.001					<0.001
Peru (*n* = 701)	Puno (*n* = 339)	<7	26	25.2% (17.4, 34.9)	11	28.2% (15.8, 43.7)	15	23.4% (14.3, 34.9)	
		7–9	28	27.2% (19.1, 37.0)	13	25.5% (15.0, 38.7)	15	28.9% (17.8, 42.2)	
		10–12	25	24.0% (16.4, 33.6)	11	28.2% (15.8, 43.7)	14	21.5% (12.8, 32.7)	
		13–15	4	14.3% (4.7, 33.6)	1	8.3% (0.5, 34.7)	3	18.7% (5.0, 43.0)	
		16–18	-	-	-	-	-	-	
		>18	-	-	-	-	-	-	
	Total		83	24.5% (20.1, 29.6)	36	25.5% (18.8, 33.2)	47	23.9% (18.3, 30.2)	
	*p*-value *			<0.001					<0.001
	Cajamaca (*n* = 362)	<7	21	20.8% (13.6, 30.2)	10	22.2% (11.9, 36.0)	11	20.7% (11.4, 33.2)	
		7–9	21	19.1% (12.5, 27.9)	12	20.3% (11.5, 32.0)	9	17.3% (8.8, 29.4)	
		10–12	26	23.8% (16.4, 33.1)	13	26.5% (15.6, 40.1)	13	21.7% (12.6, 33.4)	
		13–15	8	25.0% (12.1, 43.7)	2	14.3% (2.5, 39.7)	6	33.3% (14.8, 56.9)	
		16–18	1	25.0% (1.3, 78.0)	-	-	1	50.0% (2.5, 97.5)	
		>18	1	16.7% (0.9, 66.5)	1	20.0% (1.0, 66.6)	-		
	Total		78	21.5% (17.5, 26.2)	38	21.8% (16.2, 28.4)	40	21.3% (15.9, 27.6)	
	*p*-value *			<0.05					<0.001
Egypt (*n* = 679)		<7	13	8.5% (4.8, 13.8)	10	10.4% (5.4, 17.8)	3	5.4% (1.4, 13.9)	
		7–9	8	30.7% (15.4, 50.2)	6	35.3% (15.7, 59.5)	2	22.2% (3.9, 56.2)	
		10–12	12	14.8% (8.3, 23.8)	8	17.8% (8.6, 31.0)	4	11.1% (3.6, 24.7)	
		13–15	11	16.7% (9.1, 27.1)	8	17.4% (8.4, 30.4)	3	15.0% (4.0, 35.6)	
		16–18	4	8.7% (2.8, 19.6)	4	11.8% (3.8, 26.0)	-	-	
		>18	40	13.0% (9.5, 17.4)	31	9.8% (6.3, 14.8)	9	9.6% (4.7, 17.8)	
	Total		88	13.0% (10.6, 15.7)	67	14.8% (11.7, 18.5)	21	6.7% (6.2, 14.6)	
	*p*-value *			<0.001					<0.001

PC, positive cases. CI, confidence interval. Age, sex and geographically stratified data from transversal studies of *Fasciola* in the northern Bolivian Altiplano (Aymara), the Peruvian Altiplano (Quechua), the Cajamarca valley of Peru (Quechua) and the lowland areas of the Nile Delta in Egypt. * *p*-value < 0.005 (based on chi-square test).

**Table 4:** Comparison of coinfections in *Fasciola*-infected subjects and in subjects not infected by *Fasciola*, in the total of subjects studied, combining the endemic areas of the Northern Bolivian Altiplano, Peruvian Altiplano, Cajamarca valley and the Nile Delta. Analysis stratified according to the number of coinfecting parasite species.

	*Fasciola*-Infected Subjects			Subjects Not Infected by *Fasciola*			*p*-Value ^b^
	N	Prevalence (%)	(95% CI)	*n*	Prevalence (%)	(95% CI)	
Parasite species (protozoans * and helminths)							
0	22	4.89	(3.25, 7.29)	220	10.36	(9.13, 11.72)	0.0002
1	45	10.00	(7.56, 13.12)	266	12.53	(11.18, 13.99)	0.1517
2	84	18.67	(15.34, 22.53)	364	17.15	(15.59, 18.79)	0.4517
3	85	18.89	(15.54, 22.76	443	20.87	(19.17, 22.63)	0.3686
4	87	19.33	(15.95, 23.24	358	16.86	(15.32, 18.50)	0.2168
5	57	12.67	(9.91, 16.06)	244	11.49	(10.20, 12.91)	0.4684
6	44	9.78	(7.37, 12.87)	152	7.16	(6.14, 8.33)	0.0629
7	22	4.89	(3.25, 7.28)	65	3.06	(2.41, 3.88)	0.061
8	4	0.89	(0.35, 2.26)	11	0.52	(0.29, 0.93)	0.3151
9	0	0.00	(0.00, 0.85)	2	0.09	(0.02, 0.34)	-
Total	450			2125			
Protozoan * species							
0	29	6.44	(4.53, 9.11)	273	12.86	(11.49, 13.34)	<0.0001
1	59	13.11	(10.30, 16.54)	286	13.47	(12.07, 14.98)	0.8791
2	101	22.44	(18.83, 26.52)	433	20.40	(18.72, 22.14)	0.3373
3	95	21.11	(17.59, 25.12)	483	22.75	(20.99, 24.56)	0.494
4	70	15.56	(12.50, 19.19)	325	15.31	(13.83, 16.89)	0.8857
5	57	12.67	(9.90, 16.06)	197	9.28	(8.11, 10.58)	0.0364
6	32	7.11	(5.08, 9.87)	104	4.90	(4.06, 5.90)	0.0632
7	7	1.56	(0.76, 3.18)	23	1.08	(0.72, 1.62)	0.4652
8	0	0.00	(0.00, 0.85)	1	0.05	(0.00, 0.27)	-
Total	450			2125			
**Helminth species ^a^**							
0	0	0.00	(0.00, 0.845)	1492	70.28	(68.23, 72.12)	<0.0001
1	284	63.11	(58.56, 67.44)	511	24.07	(22.28, 25.91)	<0.0001
2	135	30.00	(29.95, 34.40)	108	5.09	(4.23, 6.10)	<0.0001
3	29	6.44	(4.52, 9.10)	14	0.66	(0.39, 1.10)	<0.0001
4	2	0.44	(0.08, 1.61)	0	0.00	(0.00, 0.18)	-
**Total**	450			2125			

^a^ The presence of *Fasciola* spp. is not included in the number of helminths. ^b^ *p*-value < 0.05 (based on Pearson test). CI confidence interval. * Including *Blastocystis* sp.

**Table 5:** Associations between *Fasciola* infection and infection by the protozoan and helminth species found simultaneously coexisting in the same subject in the endemic areas of the Northern Bolivian Altiplano, Peruvian Altiplano, Cajamarca valley and the Nile Delta (2011–2023), among the entire population, the population 18 years old or younger, and the population above 18 years old.

Parasite Species	*Fasciola*-Infected Subjects						Subjects Not Infected by *Fasciola*						All Cases	≤18 Years	>18 Years
	All Cases		≤18 Years		>18 Years		All Cases		≤18 Years		>18 Years		*p*- Value ^g^	*p*- Value ^h^	*p*- Value ^i^
	*n*	%	*n*	%	*n*	%	*n*	%	*n*	%	*n*	%			
Protozoa *															
*Blastocystis* sp.	238	52.89	219	53.55	19	47.50	1110	52.24	999	54.00	112	40.43	0.835	0.870	0.397
*Entamoeba coli*	356	79.11	340	83.13	16	40.00	1492	70.21	1408	76.11	86	31.05	0.0001	0.002	0.279
*Entamoeba histolytica* ^a^	94	20.89	87	21.27	7	17.50	446	20.99	426	23.03	20	7.22	>0.999	0.473	0.061
*Entamoeba hartmanni*	147	32.67	138	33.74	9	22.50	575	27.06	539	29.14	37	13.36	0.017	0.074	0.148
*Endolimax nana*	208	46.22	203	49.63	5	12.50	861	40.52	833	45.03	29	10.47	0.027	0.090	0.784
*Iodamoeba buetschlii*	117	26.00	114	27.87	3	7.50	395	18.59	386	20.86	10	3.61	0.0006	0.002	0.217
*Chilomastix mesnili*	29	6.44	27	6.60	2	5.00	140	6.59	137	7.41	3	1.08	>0.999	0.674	0.121
*Giardia intestinalis*	128	28.44	125	30.56	3	7.50	491	23.11	461	24.92	30	10.83	0.017	0.021	0.781
*Enteromonas hominis*	7	1.56	0	0.00	1	2.50	23	1.08	8	0.43	4	1.44	0.465	0.383	0.493
*Dientamoeba fragilis* ^b^	2	0.99	2	1.00	0	0.00	1	0.10	1	0.10	0	0.00	0.081	0.075	
*Cryptosporidium* sp. ^b^	26	12.94	26	12.94	0	0.00	124	5.83	124	12.50	0	0.00	0.387	0.908	
*Balantidium coli*	3	0.67	3	0.73	0	0.00	21	0.98	20	1.08	1	0.36	0.786	0.785	0.260
**Helminths**															
*Schistosoma mansoni* ^c^	18	20.45	14	29.17	4	10.26	64	10.82	39	12.07	25	9.33	0.043	0.004	0.774
*Hymenolepis nana*	51	11.33	50	12.22	1	2.50	199	9.36	196	10.59	3	1.08	0.219	0.335	0.419
*Taenia* ssp	11	2.44	11	2.69	0	0.00	49	2.31	49	2.65	0	0.00	0.863	>0.999	
*Trichuris trichiura*	43	9.56	42	10.27	1	2.50	163	7.67	163	8.81	0	0.00	0.181	0.343	0.126
*Ascaris lumbricoides*	47	10.44	45	11.00	1	2.50	188	8.85	186	10.05	2	0.72	0.280	0.588	0.334
Ancylostomatidae spp. ^d^	2	0.54	2	0.49	0	0.00	3	0.16	3	0.16	0	0.00	0.212	0.169	
*Strongyloides stercolaris* ^e^	0	0.00	0	0.00	0	0.00	1	0.39	1	0.39	0	1.08	>0.999	0.552	
*Enterobius vermicularis*	27	6.00	26	6.36	1	2.50	102	4.80	99	5.35	3	0.00	0.285	0.445	0.419
STH ^f^	350	77.78	93	22.74	6	15.00	375	17.65	348	18.81	27	9.75	<0.0001	0.073	0.280

* Including *Blastocystis* sp. ^a^ *Entamoeba histolytica* complex. ^b^ Only present in Bolivia. ^c^ Only present in Egypt. ^d^ Ancylostoma *duodenale* and/or *Necator americanus.* ^e^ Only present in Puno (Peru). ^f^ STH soil-transmitted nematode species (including *Ascaris lumbricoides*, *Trichuris trichiura* and *Ancylostoma duodenale* and/or *Necator americanus*)*. n* = number of cases. *p*-value based on Fisher’s exact test/Yates continuity corrected chi-square test (comparison between entire population ^g^, population 18 years old or younger ^h^ and population above 18 years ^i^).

**Table 6:** Prevalence of parasites in monoinfection and coinfection in individuals with fascioliasis and non-fascioliasis in the four areas analyzed together (Northern Bolivian Altiplano, Peruvian Altiplano, Cajamarca valley and the Nile Delta) (2011–2023) from the entire population, the population 18 years old or younger and the population above 18 years.

Parasite Species	*Fasciola*-Infected Subjects						Subjects Not Infected by *Fasciola*						All Cases	≤18 Years	>18 Years
	All Cases		≤18 Years		>18 Years		All Cases		≤18 Years		>18 Years		*p*- Value ^a^	*p*- Value ^b^	*p*- Value ^c^
	*n*	%	*n*	%	*n*	%	*n*	%	*n*	%	*n*	%			
Monoinfection by protozoan * parasites	39	8.67	28	6.85	11	26.83	216	10.2	167	9.03	50	18.05	0.385	0.173	0.203
Monoinfection by helminth parasites	6	1.33	3	0.73	3	7.32	50	2.4	35	1.89	15	5.42	0.214	0.134	0.714
Coinfection by protozoan *–helminth parasites	159	35.33	154	37.65	5	12.20	580	27.3	562	30.38	18	6.50	0.001 *	0.005 *	0.196
Coinfection by protozoan *–protozoan parasites	223	49.56	207	50.61	16	39.02	1056	49.7	964	52.11	93	33.57	0.959	0.585	0.496
Coinfection by helminth–helminth parasites	1	0.22	1	0.24	0	0.00	3	0.1	3	0.16	0	0.00	0.536	0.556	-
Negative	22	4.89	16	3.91	6	14.63	220	10.4	119	6.43	101	36.46	0.0001 *	0.051 *	0.005 *

*n* = number of cases = absolute frequency. % relative frequency. * including *Blastocystis* sp. *p*-value based on Fisher’s exact test/Yates continuity corrected chi-square test (comparison between entire population ^a^, population 18 years old or younger ^b^ and the population above 18 years **^c^**).

**Table 7:** Results of multivariate logistic regression analysis, using four models including presence/absence of fascioliasis as dependent variable. For the characteristics of Models 1 to 4 see Methods. Results were considered statistically significant when *p* < 0.05.

	Model 1			Model 2			Model 3			Model 4		
	OR	(95% CI)	*p*-Value	OR	(95% CI)	*p*-Value	OR	(95% CI)	*p*-Value	OR	(95% CI)	*p*-Value
Geographical location				1.02	(0.87, 1.16)	0.98						
Sex				0.92	(0.71, 1.18)	0.51						
Age				1.81	(1.17, 2.80)	0.007	0.69	(0.48, 0.97)	0.03	0.91	(0.63, 1.32)	0.63
Protozoans *	1.03	(0.96, 1.11)	0.37	1.07	(0.99, 1.16)	0.10						
Helminths	7.51	(6.25, 9.03)	<0.001	7.78	(6.50, 9.46)	<0.001						
*E. coli*										1.37	(1.04, 1.80)	0.02
*E. hartmanni*										1.09	(0.86, 1.39)	0.43
*E. nana*										1.08	(0.86, 1.34)	0.48
*I. buetschlii*										1.32	(1.02, 1.72)	0.03
*G. intestinalis*										1.26	(1.00, 1.59)	0.04
STH							1.31	(1.01, 1.67)	0.03	1.28	(1.00, 1.65)	0.04

* including *Blastocystis* sp. OR = odds ratio. CI = confidence interval.

## Data Availability

The original contributions presented in this study are included in the article. Further inquiries can be directed to the corresponding author.

## Supplementary Materials

The following supporting information can be downloaded at: https://www.mdpi.com/article/10.3390/tropicalmed10080224/s1, Supplement S1: Human fascioliasis hyperendemic areas surveyed; Supplement S2: Figure showing prevalence of parasites in coinfection in individuals with fascioliasis (F) and non-fascioliasis (non-F) in the four areas analyzed independently; Supplement S3: Figure showing adjusted OR for fascioliasis with the reference individuals without fascioliasis.

## Abbreviations

The following abbreviations are used in this manuscript:

WHO	World Health Organization
NTD	Neglected tropical diseases
epg	Eggs/gram of feces
SD	Standard deviation
CI	Confidence interval
*Bh*	*Blastocystis hominis*
*Ec*	*Entamoeba coli*
*Ehi*	*Entamoeba histolytica* complex
*Eha*	*Entamoeba hartmanni*
*Ena*	*Endolimax nana*
*Ib*	*Iodamoeba buetschlii*
*Cm*	*Chilomastix mesnili*
*Gi*	*Giardia intestinalis*
*Eho*	*Enteromonas hominis*
*Df*	*Dientamoeba fragilis*
Cr	*Cryptosporidium* sp.
Bc	*Balantidium coli*
STH	Soil-transmitted helminths
Sm	*Schistosoma mansoni*
Hn	*Hymenolepis nana*
Tae	*Taenia* sp.
Tt	*Trichuris trichiura*
Al	*Ascaris lumbricoides*
An	*Ancylostoma duodenale* and/or *Necator americanus*
Ss	*Strongyloides stercolaris*
Ev	*Enterobius vermicularis*
OR	Odds ratio
DALY	Disability-adjusted life year

## References

[1] Mas-Coma S., Valero M.A., Bargues M.D. (2022). Human and animal fascioliasis: Origins and worldwide evolving scenario. Clin. Microbiol. Rev..

[2] Bargues M.D., Halajian A., Artigas P., Luus-Powell W.J., Valero M.A., Mas-Coma S. (2022). Paleobiogeographical origins of *Fasciola hepatica* and *F. gigantica* in light of new DNA sequence characteristics of *F. nyanzae* from hippopotamus. Front. Vet. Sci..

[3] Mas-Coma S., Buchon P., Funatsu I.R., Angles R., Artigas P., Valero M.A., Bargues M.D. (2020). Sheep and cattle reservoirs in the highest human fascioliasis hyperendemic area: Experimental transmission capacity, field epidemiology, and control within a One Health initiative in Bolivia. Front. Vet. Sci..

[4] Mas-Coma S., Buchon P., Funatsu I.R., Angles R., Mas-Bargues C., Artigas P., Valero M.A., Bargues M.D. (2020). Donkey fascioliasis within a One Health control action: Transmission capacity, field epidemiology, and reservoir role in a human hyperendemic area. Front. Vet. Sci..

[5] Mas-Coma S., Funatsu I.R., Angles R., Buchon P., Mas-Bargues C., Artigas P., Valero M.A., Bargues M.D. (2021). Domestic pig prioritized in one health action against fascioliasis in human endemic areas: Experimental assessment of transmission capacity and epidemiological evaluation of reservoir role. One Health.

[6] Mas-Coma S., Cafrune M.M., Funatsu I.R., Mangold A.J., Angles R., Buchon P., Fantozzi M.C., Artigas P., Valero M.A., Bargues M.D. (2021). Fascioliasis in llama, *Lama glama*, in Andean endemic areas: Experimental transmission capacity by the high altitude snail vector *Galba truncatula* and epidemiological analysis of Its reservoir role. Animals.

[7] Hayward A.D., Skuce P.J., McNeill T.N. (2021). The influence of liver fluke infection on production in sheep and cattle: A meta-analysis. Int. J. Parasitol..

[8] Bargues M.D., Valero M.A., Trueba G.A., Fornasini M., Villavicencio A.F., Guaman R., De Elias-Escribano A., Perez-Crespo I., Artigas P., Mas-Coma S. (2021). DNA multi-marker genotyping and CIAS morphometric phenotyping of *Fasciola gigantica*-sized flukes from Ecuador, with an analysis of the *Radix* absence in the New World and the evolutionary lymnaeid snail vector filter. Animals.

[9] Mas-Coma S. (2005). Epidemiology of fascioliasis in human endemic areas. J. Helminthol..

[10] Mas-Coma S., Valero M.A., Bargues M.D. (2024). Fascioliasis. Adv. Exp. Med. Biol..

[11] Afshan K., Fortes-Lima C.A., Artigas P., Valero M.A., Qayyum M., Mas-Coma S. (2014). Impact of climate change and man-made irrigation systems on the transmission risk, long-term trend and seasonality of human and animal fascioliasis in Pakistan. Geospat. Health.

[12] Qureshi A.W., Tanveer A., Mas-Coma S. (2016). Epidemiological analysis of human fascioliasis in northeastern Punjab, Pakistan. Acta Trop..

[13] Qureshi A.W., Zeb A., Mansoor A., Hayat A., Mas-Coma S. (2019). *Fasciola hepatica* infection in children actively detected in a survey in rural areas of Mardan district, Khyber Pakhtunkhawa province, northern Pakistan. Parasitol. Int..

[14] Sunita K., Mas-Coma S., Bargues M.D., Sadaf Khan M.A., Habib M., Mustafa S., Husain S.A. (2021). Buffalo infection by *Fasciola gigantica* transmitted by *Radix acuminata* in Uttar Pradesh, India: A molecular tool to improve snail vector epidemiology assessments and control surveillance. Acta Parasitol..

[15] Bargues M.D., Artigas P., Varghese G.M., John T.J., Ajjampur S.S.R., Ahasan S.A., Chowdhury E.H., Gabrielli A.F., Mas-Coma S. (2024). Human fascioliasis emergence in southern Asia: Complete nuclear rDNA spacer and mtDNA gene sequences prove Indian patient infection related to fluke hybridization in northeastern India and Bangladesh. One Health.

[16] De N.V., Minh P.N., Le T.H., Dung D.T., Duon T.T., Tuan B.V., Dong L.T., Chau N.V.V., Cuervo P.F., Bargues M.D. (2024). A multidisciplinary analysis of over 53,000 fascioliasis patients along the 1995–2019 countrywide spread in Vietnam defines a new epidemiological baseline for One Health approaches. One Health.

[17] Mas-Coma S. (2020). Human fascioliasis emergence risks in developed countries: From individual patients and small epidemics to climate and global change impacts. Enf. Infec. Microbiol.Clin..

[18] Mas-Coma S., Valero M.A., Bargues M.D. (2009). *Fasciola*, lymnaeids and human fascioliasis, with a global overview on disease transmission, epidemiology, evolutionary genetics, molecular epidemiology and control. Adv. Parasitol..

[19] Mas-Coma S., Bargues M.D., Valero M.A. (2005). Fascioliasis and other plant-borne trematode zoonoses. Int. J. Parasitol..

[20] WHO (2013). Sustaining the Drive to Overcome the Global Impact of Neglected Tropical Diseases, Department of Control of Neglected Tropical Diseases.

[21] WHO (2020). Ending the Neglect to Attain the Sustainable Development Goals. A Road Map for Neglected Tropical Diseases 2021–2030.

[22] Valero M.A., Perez-Crespo I., Chillón-Marinas C., Khoubbane M., Quesada C., Reguera-Gomez M., Mas-Coma S., Fresno M., Gironès N. (2017). *Fasciola hepatica* reinfection potentiates a mixed Th1/Th2/Th17/Treg response and correlates with the clinical phenotypes of anemia. PLoS ONE.

[23] Valero M.A., Gironès N., Reguera-Gomez M., Pérez-Crespo I., López-García M.P., Quesada C., Bargues M.D., Fresno M., Mas-Coma S. (2020). Impact of fascioliasis reinfection on *Fasciola hepatica* egg shedding: Relationship with the immune-regulatory response. Acta Trop..

[24] Mas-Coma S., Valero M.A., Bargues M.D. (2023). One Health for fascioliasis control in human endemic areas. Trends Parasitol..

[25] Valero M.A., Bargues M.D., Khoubbane M., Artigas P., Quesada C., Berinde L., Ubeira F.M., Mezo M., Hernandez J.L., Agramunt V.H. (2016). Higher physiopathogenicity by *Fasciola gigantica* than by the genetically close *F. hepatica*: Experimental long-term follow-up of biochemical markers. Trans. R. Soc. Trop. Med. Hyg..

[26] Rondelaud D., Dreyfuss G., Vignoles P. (2006). Clinical and biological abnormalities in patients after fasciolosis treatment. Med. Mal. Infect..

[27] Chen M.G., Mott K.E. (1990). Progress in assessment of morbidity due to *Fasciola hepatica* infection: A review of recent literature. Trop. Dis. Bull..

[28] Mas-Coma S., Bargues M.D., Marty A.M., Neafie R.C., Meyers W.M., Neafie R.C., Marty A.M., Wear D.J. (2000). Hepatic Trematodiases. Pathology of Infectious Diseases, Helminthiases.

[29] Mas-Coma S., Agramunt V.H., Valero M.A. (2014). Neurological and ocular fascioliasis in humans. Adv. Parasitol..

[30] González-Miguel J., Valero M.A., Reguera-Gómez M., Mas-Bargues C., Bargues M.D., Simón F., Mas-Coma S. (2019). Numerous *Fasciola* plasminogen-binding proteins may underlie blood-brain barrier leakage and explain neurological disorder complexity and heterogeneity in the acute and chronic phases of human fascioliasis. Parasitology.

[31] Serrat J., Becerro-Recio D., Torres-Valle M., Simón F., Valero M.A., Bargues M.D., Mas-Coma S., Siles-Lucas M., González-Miguel J. (2023). *Fasciola hepatica* juveniles interact with the host fibrinolytic system as a potential early-stage invasion mechanism. PLoS Negl. Trop. Dis..

[32] Steinmann P., Du Z.W., Utzinger J., Zhou X.N. (2010). Multiparasitism a neglected reality on global, regional and local scale. Adv. Parasitol..

[33] Garza-Cuartero L., Garcia-Campos A., Zintl A., Chryssafidis A., O’Sullivan J., Sekiya M., Mulcahy G. (2014). The worm turns: Trematodes steering the course of co-infections. Vet. Pathol..

[34] Vaumourin E., Vourch G., Gasqui P., Vayssier-Taussat M. (2015). The importance of multiparasitism: Examining the consequences of co-infections for human and animal health. Parasites Vectors.

[35] Payne R.J.H., Turner L., Morgan E.R. (2009). Inappropriate measures of population health for parasitic disease?. Trends Parasitol..

[36] Rousseau D., Le Fichoux Y., Stien X., Suffia I., Ferrua B., Kubar J. (1997). Progression of visceral leishmaniasis due to Leishmania infantum. BALB/c mice is markedly slowed by prior infection with *Trichinella spiralis*. Infect. Immun..

[37] Yan Y., Inuo G., Akao N., Tsukidate S., Fujita K. (1997). Down-regulation of murine susceptibility to cerebral malaria by inoculation with third-stage larvae of the filarial nematode *Brugia pahangi*. Parasitology.

[38] Thomas F.P.J.F., Guegan Y., Michalakis Y., Renaud F. (2000). Are there pros as well as cons to being parasitized. Parasitol. Today.

[39] Elelu N., Ambali A., Coles G.C., Eisler M.C. (2016). Cross-sectional study of *Fasciola gigantica* and other trematode infections of cattle in Edu Local Government Area, Kwara State, north-central Nigeria. Parasites Vectors.

[40] Che-Kamaruddin N., Isa N.M.M. (2023). Assessment of *Fasciola* and paramphistomes co-infection in large ruminants through faecal egg counts around Taiping, Malaysia. Trop. Biomed..

[41] Vogel D.W. (1971). Experimental studies on mixed infection of cattle with hydatid cyst and *Fasciola*. Abst. Vet. J..

[42] Hidalgo C., Stoore C., Hernández M., Paredes R. (2020). *Fasciola hepatica* coinfection modifies the morphological and immunological features of *Echinococcus granulosus* cysts in cattle. Vet. Res..

[43] Stoore C., Andrade C., Hidalgo C., Corrêa F., Jiménez M., Hernandez M., Paredes R. (2018). *Echinococcus granulosus* hydatid cyst location is modified by *Fasciola hepatica* infection in cattle. Parasites Vectors.

[44] Petraglia A.A. (1954). Parasitosis humana por *Fasciola hepatica*: Primer caso que se describe en el Noreste Argentino. Act. Trab. Asoc. Arg. Est. Enf. Transm..

[45] Froyd G. (1960). The incidence of liver flukes *(Fasciola gigantica)* and hydatid cysts *(Echinococcus granulosus)* in Kenya cattle. J. Parasitol..

[46] Strada L. (1961). Fascioliasis hepática humana. Prensa Méd. Arg..

[47] Dawes B. (1963). Hyperplasia of the bile duct in fascioliasis and its relation to the problem of mixed infection of *Fasciola* and hydatid cysts. Parasitology.

[48] Peiretti J.A. (1969). Distomatosis hepática, contribución al diagnóstico precoz. Día. Médico..

[49] Debray C., Paolaggi J.A., Cerf M., Benhamou G., Morin T., Gosset F. (1975). Association of hepatic hidatidosis (3 cysts) and choledochal distomiasis. Sem. Hôp. Paris.

[50] Duron J.J., Benhamou G., Nardi C. (1975). Association of a hydatid cyst and distomatosis of the liver. Nouv. Presse Méd..

[51] Miguel C.M., Mallea Gil M.S., Basile M.A., Mauro E.L. (1985). Distomatosis por *Fasciola hepatica*. Prensa Méd. Arg..

[52] Mera y Sierra R., Agramunt V.H., Cuervo P., Mas-Coma S. (2011). Human fascioliasis in Argentina: Retrospective overview, critical analysis and baseline for future research. Parasites Vectors.

[53] Şakru N., Korkmaz M., Demirci M., Kuman A., Ok U.Z. (2011). *Fasciola hepatica* infection in echinococcosis suspected cases. Turk. J. Parasitol..

[54] Kaya M., Bestas R., Girgin S., Cicek M., Kaplan M.A. (2012). Increased anti-*Echinococcus granulosus* antibody positivity in *Fasciola hepatica* infection. Turk. J. Gastroenterol..

[55] Kim T.Y., Lee Y.S., Yun J.H., Kim J.J., Choi W.H., Oh I.H., Song H.O., Chu J.P. (2010). A case of probable mixed-infection with *Clonorchis sinensis* and *Fasciola* sp.: CT and parasitological findings. Kor. J. Parasitol..

[56] Wong R.K., Peura D.A., Mutter M.L., Heit H.A., Birns M.T., Johnson L.F. (1985). Hemobilia and liver flukes in a patient from Thailand. Gastroenterology.

[57] Doğan N., Koçman N.U. (2013). Uzun süreli karın ağrısı sikayeti olan hastada poliparazitizm olgusu [Case of polyparasitism with long-term abdominal pain in a patient]. Turk. J. Parasitol..

[58] Wang M., Liu W., Xiong Z., Li Z., Li J., Xu X., Zhang M., Xing M., Ning Q., Wu D. (2022). Case report: “Area of Focus” atypical trichinellosis and fascioliasis coinfection. Front. Med..

[59] Torrus-Tendero D., Ramos-Rincón J.M., Salvador F., Oliveira I., Llenas-García J., Arsuaga M., Crespillo-Andújar C., Pérez-Molina J.A. (2022). Imported fascioliasis in Spain: Report of 12 cases from the +REDIVI collaborative network (2009–2019). Travel Med. Infect. Dis..

[60] Kim S.W., Jang B.K. (2023). *Toxocara canis* and *Fasciola hepatica* co-infection leading to hepatic abscess: A case report. J. Kor. Med. Sci..

[61] Oujamaa L., Sibon I., Vital A., Menegon P. (2023). Vasculite cérébrale secondaire à une co-infestation par *Toxocara canis* et *Fasciola hepatica*. Rev. Neurol..

[62] Zumaquero-Ríos J.L., Sarracent-Pérez J., Rojas-García R., Rojas-Rivero L., Martínez-Tovilla Y., Valero M.A., Mas-Coma S. (2013). Fascioliasis and intestinal parasitoses affecting schoolchildren in Atlixco, Puebla State, Mexico: Epidemiology and treatment with nitazoxanide. PLoS Negl. Trop. Dis..

[63] Graham C.S., Brodie S.B., Weller P.F. (2001). Imported *Fasciola hepatica* infection in the United States and treatment with triclabendazole. Clin. Infect. Dis..

[64] Miller C.M., Smith N.C., Ikin R.J., Boulter N.R., Dalton J.P., Donnelly S. (2009). Immunological interactions between 2 common pathogens, Th1-inducing protozoan *Toxoplasma gondii* and the Th2-inducing helminth *Fasciola hepatica*. PLoS ONE.

[65] Deveci Ö., Aslan E., Tekin A., Toka Özer T., Tekin R., Bozkurt F., Çetinçakmak M.G. (2014). Ayni hastada fascioliazis ve bruselloz [fascioliasis and brucellosis in same patient]. Turk. J. Parasitol..

[66] Önal U., Yamazhan T., Pullukçu H., Tasbakan M., Tamsel S., Erdogan D.D., Korkmaz M., Sipahi O.R. (2017). Two rare causes of hepatitis: Fascioliasis and brucellosis. J. Viral Hepat..

[67] Garza-Cuartero L., O’Sullivan J., Blanco A., McNair J., Welsh M., Flynn R.J., Williams D., Diggle P., Cassidy J., Mulcahy G. (2016). *Fasciola hepatica* infection reduces *Mycobacterium bovis* burden and mycobacterial uptake and suppresses the pro-inflammatory response. Parasite Immunol..

[68] Demirci M., Isler M., Cicioglu Aridogan B., Senoi A., Korkma M. (2004). Coinfection of chronic hepatitis B and fasciolosis. Infection.

[69] Mas-Coma S., Bargues M.D., Esteban J.G., Dalton J.P. (1999). Human fasciolosis. Fasciolosis.

[70] Bjorland J., Bryan R.T., Strauss W., Hillyer G.V., McAuley J.B. (1995). An outbreak of acute fascioliasis among Aymara Indians in the Bolivian Altiplano. Clin. Infect. Dis..

[71] Mas-Coma S., Angles R., Esteban J.G., Bargues M.D., Buchon P., Franken M., Strauss W. (1999). The human fascioliasis high endemic region of the Northern Bolivian Altiplano. Trop. Med. Int. Health.

[72] Esteban J.G., Flores A., Aguirre C., Strauss W., Angles R., Mas-Coma S. (1997). Presence of very high prevalence and intensity of infection with *Fasciola hepatica* among Aymara children from the Northern Bolivian Altiplano. Acta Trop..

[73] Esteban J.G., Flores A., Angles R., Strauss W., Aguirre C., Mas-Coma S. (1997). A population-based coprological study of human fascioliasis in a hyperendemic area of the Bolivian Altiplano. Trop. Med. Int. Health.

[74] Esteban J.G., Flores A., Angles R., Mas-Coma S. (1999). High endemicity of human fascioliasis between Lake Titicaca and La Paz valley, Bolivia. Trans. R. Soc. Trop. Med. Hyg..

[75] Angles R., Strauss W., Ramirez S., Esteban J.G., Mas-Coma S. (1997). Human fascioliasis in Bolivia: Coprological surveys in different provinces of the Department of La Paz. Res. Rev. Parasitol..

[76] Hillyer G.V., Soler de Galanes M., Rodriguez-Perez J., Bjorland J., Silva de Lagrava M., Ramirez Guzman S., Bryan R.T. (1992). Use of the Falcon Assay Screening Test—Enzyme-Linked Immunosorbent Assay (FAST-ELISA) and the Enzyme-Linked Immunoelectrotransfer Blot (EITB) to determine the prevalence of human fascioliasis in the Bolivian Altiplano. Am. J. Trop. Med. Hyg..

[77] O’Neill S.M., Parkinson M., Strauss W., Anglés R., Dalton J.P. (1998). Immunodiagnosis of *Fasciola hepatica* (Fascioliasis) in a human population in the Bolivian Altiplano using purified cathepsin L cysteine proteinase. Am. J. Trop. Med. Hyg..

[78] Valero M.A., Periago M.V., Perez-Crespo I., Angles R., Villegas F., Aguirre C., Strauss W., Espinoza J.R., Herrera P., Terashima A. (2012). Field evaluation of a coproantigen detection test for fascioliasis diagnosis and surveillance in human hyperendemic areas of Andean countries. PLoS Negl. Trop. Dis..

[79] Mas-Coma S., Funatsu I.R., Bargues M.D. (2001). *Fasciola hepatica* and lymnaeid snails occurring at very high altitude in South America. Parasitology.

[80] Bargues M.D., Artigas P., Angles R., Osca D., Duran P., Buchon P., Gonzales-Pomar R.K., Pinto-Mendieta J., Mas-Coma S. (2020). Genetic uniformity, geographical spread and anthropogenic habitat modifications of lymnaeid vectors found in a One Health initiative in the highest human fascioliasis hyperendemic of the Bolivian Altiplano. Parasit. Vectors.

[81] Bargues M.D., Angles R., Coello J., Artigas P., Funatsu I.R., Cuervo P.F., Buchon P., Mas-Coma S. (2021). One Health initiative in the Bolivian Altiplano human fascioliasis hyperendemic area: Lymnaeid biology, population dynamics, microecology and climatic factor influences. Braz. J. Vet. Parasitol..

[82] Villegas F., Angles R., Barrientos R., Barrios G., Valero M.A., Hamed K., Grueningr H., Ault S.K., Montresor A., Engels D. (2012). Administration of triclabendazole is safe and effective in controlling fascioliasis in an endemic community of the Bolivian Altiplano. PLoS Negl. Trop. Dis..

[83] Esteban J.G., González C., Bargues M.D., Angles R., Sánchez C., Náquira C., Mas-Coma S. (2002). High fascioliasis infection in children linked to a man-made irrigation zone in Peru. Trop. Med. Int. Health.

[84] Mas-Coma S., Bargues M.D., Valero M.A. (2018). Human fascioliasis infection sources, their diversity, incidence factors, analytical methods and prevention measures. Parasitology.

[85] Angles R., Buchon P., Valero M.A., Bargues M.D., Mas-Coma S. (2022). One Health action against human fascioliasis in the Bolivian Altiplano: Food, water, housing, behavioural traditions, social aspects, and livestock management linked to disease transmission and infection sources. Int. J. Environ. Res. Public Health.

[86] Marcos L.A., Maco V., Terashima M.A., Samalvides F., Gotuzzo E. (2002). Características clínicas de la infección crónica por *Fasciola hepatica* en niños. Rev. Gastroenterol Peru.

[87] Marcos L.A., Maco V., Terashima A., Samalvides F., Espinoza J.R., Gotuzzo E. (2005). Fascioliasis in relatives of patients with *Fasciola hepatica* infection in Peru. Rev. Inst. Med. Trop. Sâo Paulo.

[88] Marcos L., Maco V., Samalvides F., Terashima A., Espinoza J.R., Gotuzzo E. (2006). Risk factors for *Fasciola hepatica* infection in children: A case-control study. Trans. R. Soc. Trop. Med. Hyg..

[89] Gonzalez L.C., Esteban J.G., Bargues M.D., Valero M.A., Ortiz P., Naquira C., Mas-Coma S. (2011). Hyperendemic human fascioliasis in Andean valleys: An altitudinal transect analysis in children of Cajamarca province, Peru. Acta Trop..

[90] Espinoza J.R., Maco V., Marcos L., Saez S., Neyra V., Terashima A., Samalvides F., Gotuzzo E., Chavarry E., Huaman M.C. (2007). Evaluation of Fas2-ELISA for the serological detection of *Fasciola hepatica* infection in humans. Am. J. Trop. Med. Hyg..

[91] Cosme-Contreras J., Burga-Hernandez A., Geldres-Moreno L., Bazan-Vasquez C. (1971). Estudio clínico y epidemiológico de la distomatosis hepática en escolares de la zona rural de Cajamarca. Rev. Peru Pediatría.

[92] Storck M.G., Venables G.S., Jennings S.M.F., Beesley J.R., Bendezu P., Capron A. (1973). An investigation of endemic fascioliasis in Peruvian village children. J. Trop. Med. Hyg..

[93] Knobloch J. (1985). Human fascioliasis in Cajamarca/Peru. II. Humoral antibody response and antigenaemia. Trop. Med. Parasitol..

[94] Knobloch J., Delgado E., Alvarez A.G., Reymann U., Bialek R. (1985). Human fascioliasis in Cajamarca/Peru. I. Diagnostic methods and treatment with praziquantel. Trop. Med. Parasitol..

[95] Ortiz P., Cabrera M., Jave J., Claxton J., Williams D. (2000). Human fascioliasis: Prevalence and treatment in a rural area of Peru. Inf. Dis. Rev..

[96] Hillyer G.V., Soler de Galanes M., Delgado Azañero E. (2001). Immunodiagnosis of human fasciolosis in children from Cajamarca, Peru. Parasitol. Día..

[97] Alban Olaya M., Ortiz J.J., Quispe Lazo T. (2002). Fasciolasis en Cajamarca. Rev. Gastroenterol Perú..

[98] Favennec L., Jave Ortiz J., Gargala G., Lopez Chegne N., Ayoub A., Rossignol J.F. (2003). Double blind, randomized, placebo-controlled study of nitazoxanide in the treatment of fascioliasis in adults and children from northern Peru. Alim. Pharmacol. Ther..

[99] Bargues M.D., Artigas P., Khoubbane M., Flores R., Glöer P., Rojas-Garcia R., Ashrafi K., Falkner G., Mas-Coma S. (2011). *Lymnaea schirazensis*, an overlooked snail distorting fascioliasis data: Genotype, phenotype, ecology, worldwide spread, susceptibility, applicability. PLoS ONE.

[100] Bargues M.D., Artigas P., Khoubbane M., Ortiz P., Naquira C., Mas-Coma S. (2012). Molecular characterisation of *Galba truncatula*, *Lymnaea neotropica* and *L. schirazensis* from Cajamarca, Peru and their potential role in transmission of human and animal fascioliasis. Parasites Vectors.

[101] Bardales-Valdivia J.N., Bargues M.D., Hoban-Vergara C., Bardales-Bardales C., Goicoechea-Portal C., Bazan-Zurita H., Del Valle-Mendoza J., Ortiz P., Mas-Coma S. (2021). Spread of the fascioliasis endemic area assessed by seasonal follow-up of rRNA ITS-2 sequenced lymnaeid populations in Cajamarca, Peru. One Health.

[102] Bargues M.D., Gayo V., Sanchis J., Artigas P., Khoubbane M., Birriel S., Mas-Coma S. (2017). DNA multigene characterization of *Fasciola hepatica* and *Lymnaea neotropica* and its fascioliasis transmission capacity in Uruguay, with historical correlation, human report review and infection risk analysis. PLoS Negl. Trop. Dis..

[103] Bargues M.D., Malandrinni J.B., Artigas P., Soria C.C., Velasquez J.N., Carnevale S., Mateo L., Khoubbane M., Mas-Coma S. (2016). Human fascioliasis endemic areas in Argentina: Multigene characterisation of the lymnaeid vectors and climatic-environmental assessment of the transmission pattern. Parasites Vectors.

[104] Claxton J.R., Zambrano H., Ortiz P., Amoros C., Delgado E., Escurra E., Clarkson M.J. (1997). The epidemiology of fasciolosis in the inter-Andean valley of Cajamarca, Peru. Parasitol. Int..

[105] Claxton J.R., Zambrano H., Ortiz P., Delgado E., Escurra E., Clarkson M.J. (1998). Strategic control of fasciolosis in the inter-Andean valley of Cajamarca, Peru. Vet. Rec..

[106] Claxton J.R., Sutherst J., Ortiz P., Clarkson M.J. (1999). The effect of cyclic temperatures on the growth of *Fasciola hepatica* and *Lymnaea viatrix*. Vet. J..

[107] Rivera-Jacinto M., Rodriguez-Ulloa C., Rojas-Huaman Y., Valdivia-Melendez Y., Saucedo-Duran T. (2010). Conocimientos, actitudes y prácticas sobre fascioliasis en madres de una zona rural andina del Norte peruano. Rev. Peru Med. Exp. Salud Públ..

[108] Soliman M.S., Angelico M., Rocchi G. (1998). Control of veterinary fascioliasis. Infectious Diseases and Public Health.

[109] Curtale F., Nabil M., El Wakeel A., Shamy M.Y. (1998). Behera Survey Team. Anaemia and intestinal parasitic infections among school age children in Behera Governorate, Egypt. J. Trop. Pediatr..

[110] Curtale F., Hammoud E.S., El Wakeel A., Mas-Coma S., Savioli L. (2000). Human fascioliasis, an emerging public health problem in the Nile Delta, Egypt. Res. Rev. Parasitol..

[111] Esteban J.G., González C., Curtale F., Muñoz-Antoli C., Valero M.A., Bargues M.D., El Sayed M., El Wakeel A., Abdel-Wahab Y., Montresor A. (2003). Hyperendemic fascioliasis associated with schistosomiasis in villages in the Nile Delta of Egypt. Am. J. Trop. Med. Hyg..

[112] Curtale F., Moursy B.E.M., El Deen M.S.S., Nabil M., El Wakeel A. (1997). Operational Research on Health, Nutrition and Environmental Needs in Behera Governorate. Final Report. Strengthening Rural Health Services in Behera, Dakalhia and Qena Governorates.

[113] Periago M.V., Valero M.A., Artigas P., Agramunt V.H., Bargues M.D., Curtale F., Mas-Coma S. (2021). Very high fascioliasis intensities in schoolchildren of Nile Delta governorates: The Old World highest burdens found in lowlands. Pathogens.

[114] Farag H.F., Salem A.I., Khalil S.S., Farahat A. (1993). Studies on human fascioliasis in Egypt. 1-Seasonality of transmission. J. Egypt. Soc. Parasitol..

[115] Curtale F., Mas-Coma S., Hassanein Y.A.E.W., Barduagni P., Pezzotti P., Savioli L. (2003). Clinical signs and household characteristics associated with human fascioliasis among rural population in Egypt: A case-control study. Parassitologia.

[116] Curtale F., Hassanein Y.A.W., Savioli L. (2005). Control of human fascioliasis by selective chemotherapy: Design, cost and effect of the first public health, school-based intervention implemented in endemic areas of the Nile Delta, Egypt. Trans. R. Soc. Trop. Med. Hyg..

[117] Ash L.R., Orihel T.C. (1987). Parasites: A Guide to Laboratory Procedures and Identification.

[118] Sapero J.J., Lawless D. (1953). The “MIF“ stain-preservation technic for the identification of intestinal protozoa. Am. J. Trop. Med. Hyg..

[119] Blagg W., Schloegel E.L., Mansour N.S., Khalaf G.I. (1955). A new concentration technic for the demonstration of protozoa and helminth eggs in feces. Am. J. Trop. Med. Hyg..

[120] Knight W.B., Hiatt R.A., Cline B.L., Ritchie L.S. (1976). A modification of the formol-ether concentration technique for increased sensitivity in detecting *Schistosoma mansoni*. Am. J. Trop. Med. Hyg..

[121] Henriksen S.A., Pohlenz J.F.L. (1981). Staining of cryptosporidia by a modified Ziehl-Neelsen technique. Acta Vet. Scand..

[122] Esteban J.G., Aguirre C., Flores A., Strauss W., Angles R., Mas-Coma S. (1998). High *Cryptosporidium* prevalences in healthy Aymara children from the Northern Bolivian Altiplano. Am. J. Trop. Med. Hyg..

[123] Kato K., Miura M. (1954). Comparative examinations. Jap. J. Parasitol..

[124] Katz N., Chaves A., Pellegrino J. (1972). A simple device for quantitative stool thick-smear technique in schistosomiasis mansoni. Rev. Inst. Med. Trop Sao Paulo.

[125] Mas-Coma S., Bargues M.D., Valero M.A. (2014). Diagnosis of human fascioliasis by stool and blood techniques: Update for the present global scenario. Parasitology.

[126] Bargues M.D., Funatsu I.R., Oviedo J.A., Mas-Coma S. (1996). Natural water, an additional source for human infection by *Fasciola hepatica* in the Northern Bolivian Altiplano. Parassitologia.

[127] Roberts C.W., Walker W., Alexander J. (2001). Sex-associated hormones and immunity to protozoan parasites. Clin. Microbiol. Rev..

[128] Curtale F., Hassanein Y.A.W., Barduagni P., Yousef M.M., El Wakeel A., Hallaj Z., Mas-Coma S. (2007). Human fascioliasis infection: Gender difference within school-age children from endemic areas of the Nile Delta, Egypt. Trans. R. Soc. Trop. Med. Hyg..

[129] De N.V., Le T.H., Agramunt V.H., Mas-Coma S. (2020). Early postnatal and preschool age infection by *Fasciola* spp.: Report of five cases from Vietnam and worldwide review. Am. J. Trop. Med. Hyg..

[130] WHO Soil-Transmitted Helminthiases. World Health Organization. https://www.who.int/health-topics/soil-transmitted-helminthiases#tab=tab_1.

[131] Flores A., Esteban J.G., Angles R., Mas-Coma S. (2001). Soil-transmitted helminth infections at very high altitude in Bolivia. Trans. R. Soc. Trop. Med. Hyg..

[132] De N.V., Minh P.N., Duyet L.V., Mas-Coma S. (2019). Strongyloidiasis in northern Vietnam: Epidemiology, clinical characteristics and molecular diagnosis of the causal agent. Parasit. Vectors.

[133] Lebu S., Kibone W., Muoghalu C.C., Ochaya S., Salzberg A., Bongomin F., Manga M. (2023). Soil-transmitted helminths: A critical review of the impact of co-infections and implications for control and elimination. PLoS Negl. Trop. Dis..

[134] Rudan I., Lawn J., Cousens S., Rowe A.K., Boschi-Pint C., Tomaskovic L., Mendoza W., Lanata C.F., Roca-Feltrer A., Carneiro I. (2005). Gaps in policy-relevant information on burden of disease in children: A systematic review. Lancet.

[135] Hillyer G.V., Apt W. (1997). Food-borne trematode infections in the Americas. Parasitol. Today.

[136] Brady M.T., O’Neill S.M., Dalton J.P., Mills K.H. (1999). *Fasciola hepatica* suppresses a protective Th1 response against *Bordetella pertussis*. Infect. Immun..

[137] Dowling D.J., Hamilton C.M., Donnelly S., La Course J., Brophy P.M., Dalton J., O’Neill S.M. (2010). Major secretory antigens of the helminth *Fasciola hepatica* activate a suppressive dendritic cell phenotype that attenuates Th17 cells but fails to activate Th2 immune responses. Infect. Immun..

[138] Vukman K.V., Adams P.N., Met M., Maurer M., O’Neill S.M. (2013). *Fasciola hepatica* tegumental coat impairs mast cells’ ability to drive Th1 immune responses. J. Immunol..

[139] Donnelly S., O’Neill S.M., Sekiya M., Mulcahy G., Dalton J.P. (2005). Thioredoxin peroxidase secreted by *Fasciola hepatica* induces the alternative activation of macrophages. Infect. Immun..

[140] Flynn R.J., Irwin J.A., Olivier M., Sekiya M., Dalton J.P., Mulcahy G. (2007). Alternative activation of ruminant macrophages by *Fasciola hepatica*. Vet. Immunol. Immunopathol..

[141] Flynn R., Mannion C., Golden O., Hacariz O., Mulcahy G. (2007). Experimental *Fasciola hepatica* infection alters responses to tests used for diagnosis of bovine tuberculosis. Infect. Immun..

[142] O’Neill S.M., Brady M.T., Callanan J.J., Mulcahy G., Joyce P., Mills K.H., Dalton J.P. (2000). *Fasciola hepatica* infection downregulates Th1 responses in mice. Parasite Immunol..

[143] Walsh K.P., Brady M.T., Finlay C.M., Boon L., Mills K.H.G. (2009). Infection with a helminth parasite attenuates autoimmunity through TGF-beta-mediated suppression of Th17 and Th1 responses. J. Immunol..

[144] Alvarez Rojas C.A., Scheerlinck J.P., Ansell B.R., Hall R.S., Gasser R.B., Jex A.R. (2016). Time-Course study of the transcriptome of peripheral blood mononuclear cells (PBMCs) from sheep Infected with *Fasciola hepatica*. PLoS ONE.

[145] Fu Y., Chryssafidis A.L., Browne J.A., O’Sullivan J., McGettigan P.A., Mulcahy G. (2016). Transcriptomic Study on Ovine Immune Responses to *Fasciola hepatica* infection. PloS Negl. Trop. Dis..

[146] Flynn R.J., Mulcahy G. (2008). The roles of IL-10 and TGF-β in controlling IL-4 and IFN-γ production during experimental *Fasciola hepatica* infection. Int. J. Parasitol..

[147] Dalton J.P., Robinson M.W., Mulcahy G., O’Neill S.M., Donnelly S. (2013). Immunomodulatory molecules of *Fasciola hepatica*: Candidates for both vaccine and immunotherapeutic development. Vet. Parasitol..

[148] Sachdev D., Gough K.C., Flynn R.J. (2017). The chronic stages of bovine *Fasciola hepatica* are dominated by CD4 T-cell exhaustion. Front. Immunol..

[149] Cwiklinski K., Donnelly S., Drysdale O., Jewhurst H., Smith D., De Marco Verissimo C., Pritsch I.C., O’Neill S., Dalton J.P., Robinson M.W. (2019). The cathepsin-like cysteine peptidases of trematodes of the genus *Fasciola*. Adv. Parasitol..

[150] Ryan S., Shiels J., Taggart C.C., Dalton J.P., Weldon S. (2020). *Fasciola hepatica*-derived molecules as regulators of the host immune response. Front. Immunol..

[151] Aron-Said C., Montes M., White A.C., Cabada M.M. (2021). Plasma cytokines during acute human fascioliasis. Parasitol. Res..

[152] Jankovic D., Liu Z., Gause W.C. (2001). Th1- and Th2-cell commitment during infectious disease: Asymmetry in divergent pathways. Trends Immunol..

[153] Cortes A., Peachey L., Scotti R., Jenkins T.P., Cantacessi C. (2019). Helminth-microbiota cross-talk—A journey through the vertebrate digestive system. Mol. Biochem. Parasitol..

[154] Homan E.J., Bremel R.D. (2018). A role for epitope networking in immunomodulation by helminths. Front. Immunol..

[155] Maizels R.M., McSorley H.J. (2016). Regulation of the host immune system by helminth parasites. J. Allergy Clin. Immunol..

[156] Yazdanbakhsh M., van den Biggelaar A., Maizels R.M. (2001). Th2 responses without atopy: Immunoregulation in chronic helminth infections and reduced allergic disease. Trends Immunol..

[157] Wohlfert E., Belkaid Y. (2008). Role of endogenous and induced regulatory cells during infections. J. Clin. Immunol..

[158] McKee A.S., Pearce E.J. (2004). CD25+CD4+ cells contribute to Th2 polarization during helminth infection by suppressing Th1 response development. J. Immunol..

[159] Cools N., Ponsaerts P., Van Tendeloo V.F., Berneman Z.N. (2007). Regulatory T cells and human disease. Clin. Dev. Immunol..

[160] Taylor M.D., LeGoff L., Harris A., Malone E., Allen J.E., Maizels R.M. (2005). Removal of regulatory T cell activity reverses hyporesponsiveness and leads to filarial parasite clearance in vivo. J. Immunol..

[161] Rodriguez-Osorio M., Gomez Garcia V., Rojas Gonzalez J., Ramajo Martin V., Manga González M.Y., Gonzalez Lanza C. (1993). Resistance to *Schistosoma bovis* in sheep induced by an experimental *Fasciola hepatica* infection. J. Parasitol..

[162] Abruzzi A., Fried B. (2011). Coinfection of *Schistosoma* (Trematoda) with bacteria, protozoa and helminths. Adv. Parasitol..

[163] Ricafrente A., Nguyen H., Tran N., Donnelly S. (2021). An evaluation of the *Fasciola hepatica* miRnome predicts a targeted regulation of mammalian innate immune responses. Front. Immunol..

[164] Girones N., Valero M.A., García-Bodelón M.A., Chico-Calero I., Punzón C., Fresno M., Mas-Coma S. (2007). Immune suppression in advanced chronic fascioliasis: An experimental study in a rat model. J. Infect. Dis..

[165] Rodriguez-Sosa M., Satoskar A.R., Calderon R., Gomez-Garcia L., Saavedra R., Bojalil R., Terrazas L.I. (2002). Chronic helminth infection induces alternatively activated macrophages expressing high levels of CCR5 with low interleukin-12 production and Th2 biasing ability. Infect. Immunol..

[166] Valero M.A., Navarro M., Garcia-Bodelon M.A., Marcilla A., Morales M., Hernandez J.L., Mengual P., Mas-Coma S. (2006). High risk of bacterobilia in advanced experimental chronic fasciolosis. Acta Trop..

[167] Deng L., Wojciech L., Gascoigne N.R.J., Peng G., Tan K.S.W. (2021). New insights into the interactions between *Blastocystis*, the gut microbiota, and host immunity. PLoS Pathog..

[168] Adam R.D. (2021). *Giardia duodenalis*: Biology and pathogenesis. Clin. Microbiol. Rev..

[169] Wojciech L., Png C.W., Koh E.Y., Kioh D.Y.Q., Deng L., Wang Z., Wu L.Z., Hamidinia M., Tung D.W., Zhang W. (2023). A tryptophan metabolite made by a gut microbiome eukaryote induces pro-inflammatory T cells. EMBO J..

[170] Veas F., Rey J.P. (1991). Infection à VIH et parasitoses en zone tropicale. Cahiers Santé.

[171] Mengist H.M., Taye B., Tsegaye A. (2015). Intestinal parasitosis in relation to CD4+ T cells levels and anemia among HAART initiated and HAART naive pediatric HIV patients in a model ART center in Addis Ababa, Ethiopia. PLoS ONE..

[172] Mülayim S., Dalkılıç S., Akbulut H.H., Aksoy A., Kaplan M. (2022). Investigation of the relationship between lymphocyte subsets and intestinal parasites. Acta Trop..

[173] Caner A., Zorbozan O., Tunalı V., Kantar M., Aydoğdu S., Aksoylar S., Gürüz Y., Turgay N. (2020). Intestinal protozoan parasitic infections in immunocompromised child patients with diarrhea. Jpn. J. Infect. Dis..

[174] Laksemi D.A., Suwanti L.T., Mufasirin M., Suastika K., Sudarmaja M. (2019). Opportunistic parasitic infections in patients with human immunodeficiency virus/acquired immunodeficiency syndrome: A review. Vet. World..

[175] Lopez-Romero G., Quintero J., Astiazarán-García H., Velazquez C. (2015). Host defenses against *Giardia lamblia*. Parasite Immunol..

[176] O’Neill S.M., Mills K.H.G., Dalton J.P. (2001). *Fasciola hepatica* cathepsin L cysteine proteinase suppresses *Bordetella pertussis*-specific interferongamma production in vivo. Parasite Immunol..

[177] Flynn R.J., Mulcahy G., Elsheikha H.M. (2010). Coordinating innate and adaptive immunity in *Fasciola hepatica* infection: Implications for control. Vet. Parasitol..

[178] Flynn R.J., Mulcahy G., Welsh M., Cassidy J.P., Corbett D., Milligan C., Andersen P., Strain S., McNair J. (2009). Co-infection of cattle with *Fasciola hepatica* and *Mycobacterium bovis*—Immunological consequences. Transb. Emerg. Dis..

[179] Howell A.K., McCann C.M., Wickstead F., Williams D.J.L. (2019). Co-infection of cattle with *Fasciola hepatica* or *F. gigantica* and *Mycobacterium bovis*: A systematic review. PLoS ONE.

[180] Aksoy D.Y., Kerimoğlu U., Oto A., Ergüven S., Arslan S., Unal S., Batman F., Bayraktar Y. (2006). *Fasciola hepatica infection*: Clinical and computerized tomographic findings of ten patients. Turk. J. Gastroenterol..

[181] Howell A.K., Tongue S.C., Currie C., Evans J., Williams D.J.L., McNeilly T.N. (2018). Co-infection with *Fasciola hepatica* may increase the risk of *Escherichia coli* O157 shedding in British cattle destined for the food chain. Prev. Vet. Med..

[182] Kelly R.F., Callaby R., Egbe N.F., Williams D.J.L., Victor N.N., Tanya V.N., Sander M., Ndip L., Ngandolo R., Morgan K.L. (2018). Association of *Fasciola gigantica* co-infection with bovine tuberculosis infection and diagnosis in a naturally infected cattle population in Africa. Front. Vet. Sci..

[183] Harris N.L., Loke P. (2017). Recent advances in type-2-cell-mediated Immunity: Insights from helminth infection. Immunity.

[184] Mishra P.K., Palma M., Bleich D., Loke P., Gause W.C. (2014). Systemic impact of intestinal helminth infections. Mucosal Immunol..

[185] Segura M., Su Z., Piccirillo C., Stevenson M.M. (2007). Impairment of dendritic cell function by excretory-secretory products: A potential mechanism for nematode-induced immunosuppression. Eur. J. Immunol..

[186] Leroux L.P., Nasr M., Valanparambil R., Tam M., Rosa B.A., Siciliani E., Hill D.E., Zarlenga D.S., Jaramillo M., Weinstock J.V. (2018). Analysis of the *Trichuris suis* excretory/secretory proteins as a function of life cycle stage and their immunomodulatory properties. Sci. Rep..

[187] Figueiredo C.A., Barreto M.L., Rodrigues L.C., Cooper P.J., Silva N.B., Amorim L.D., Alcantara-Neves N.M. (2010). Chronic intestinal helminth infections are associated with immune hyporesponsiveness and induction of a regulatory network. Infect. Immun..

[188] Wammes L.J., Hamid F., Wiria A.E., de Gier B., Sartono E., Maizels R.M., Luty A.J.F., Fillie Y., Brice G.T., Supali T. (2010). Regulatory T cell in human geohelminth infection suppress immune responses to BCG and *Plasmodium falciparum*. Eur. J. Immunol..

[189] Elias D., Britton S., Aseffa A., Engers H., Akuffo H. (2008). Poor immunogenicity of BCG in helminth infected population is associated with increased in vitro TGF-b production. Vaccine.

[190] Toulza F., Tsang L., Ottenhoff T.H., Brown M., Dockrell H.M. (2016). *Mycobacterium tuberculosis*-specific CD41 T-cell response is increased, and Treg cells decreased, in anthelmintic-treated patients with latent TB. Eur. J. Immunol..

[191] Needham C., Kim H.T., Hoa N.V., Cong L.D., Michael E., Drake L., Hall A., Bundy D.A. (1998). Epidemiology of soil-transmitted nematode infections in Ha Nam Province, Vietnam. Trop. Med. Int. Health..

[192] Brooker S., Miguel E., Moulin S., Luoba A., Bundy D., Kremer M. (2000). Epidemiology of single and multiple species of helminth infections among school children in Busia District, Kenya. East Afr. Med. J..

[193] Keiser J., N’Goran E.K., Singer B.H., Lengeler C., Tanner M., Utzinger J. (2002). Association between *Schistosoma mansoni* and hookworm infections among schoolchildren in Cote d’Ivoire. Acta Trop..

[194] Keiser J., N’Goran E.K., Traore M., Lohourignon K.L., Singer B.H., Lengeler C., Tanner M., Utzinger J. (2002). Polyparasitism with *Schistosoma mansoni*, geohelminths, and intestinal protozoa in rural Cote d’Ivoire. J. Parasitol..

[195] Tchuem Tchuente L.A., Behnke J.M., Gilbert F.S., Southgate V.R., Vercruysse J. (2003). Polyparasitism with *Schistosoma haematobium* and soil-transmitted helminth infections among school children in Loum, Cameroon. Trop. Med. Int. Health.

[196] Fleming F.M., Brooker S., Geiger S.M., Caldas I.R., Correa-Oliveira R., Hotez P.J., Bethony J.M. (2006). Synergistic associations between hookworm and other helminth species in a rural community in Brazil. Trop. Med. Int. Health.

[197] Albonico M., Allen H., Chitsulo L., Engels D., Gabrielli A.F., Savioli L. (2008). Controlling Soil-Transmitted Helminthiasis in Pre-School-Age Children through Preventive Chemotherapy. PLoS Negl. Trop. Dis..

[198] Bhengu K.N., Singh R., Naidoo P., Mpaka-Mbatha M.N., Nembe-Mafa N., Mkhize-Kwitshana Z.L. (2023). Cytokine Responses during *Mycobacterium tuberculosis* H37Rv and *Ascaris lumbricoides* costimulation using human THP-1 and Jurkat Cells, and a pilot human tuberculosis and helminth coinfection study. Microorganisms.

[199] Abou Holw S.A., El-Taweel H., El-Abd E., Osman M.M. (2007). The serum gastrin level patients with schistosomiasis and fascioliasis. J. Egypt. Soc. Parasitol..

[200] Abou-Basha L.M., Salem A., Osman M., el-Hefni S., Zaki A. (2000). Hepatic fibrosis due to fascioliasis and/or schistosomiasis in Abis 1 village, Egypt. East Mediterr. Health J..

[201] Shousha S.A., Khalil S.S., Rashwan E.A. (1999). Oxygen free radical and nitric oxide production in single or combined human schistosorniasis and fascioliasis. J. Egypt. Soc. Parasitol..

[202] Salem A.l., Basha L.M.A., Farag H.F. (1987). Immunoglobulin levels and intensity of infection in patients with fascioliasis single or combined with schistosomiasis. J. Egypt. Soc. Parasitol..

[203] Yabe J., Phiri I.K., Phiri A.M., Chembensofu M., Dorny P., Vercruysse J. (2008). Concurrent infections of *Fasciola, Schistosoma* and *Amphistomum* spp. in cattle from Kafue and Zambezi river basins of Zambia. J. Helminthol..

[204] Bundy D.A., Medley G.F. (1992). Immunoepidemiology of human geohelminthiasis: Ecological and immunological determinants of worm burden. Parasitology.

[205] Pullan R., Brooker S. (2008). The health impact of polyparasitism in humans: Are we under-estimating the burden of parasitic diseases?. Parasitology.

[206] Haswell-Elkins M.R., Elkins D.B., Anderson R.M. (1987). Evidence for predisposition in humans to infection with *Ascaris*, hookworm, *Enterobius* and *Trichuris* in a South Indian fishing community. Parasitology.

[207] Holland C.V., Asaolu S.O., Crompton D.W., Stoddart R.C., MacDonald R., Torimiro S.E. (1989). The epidemiology of *Ascaris lumbricoides* and other soil-transmitted helminths in primary school children from Ile-Ife, Nigeria. Parasitology.

[208] Chamone M., Marques C.A., Atuncar G.S., Pereira A.L., Pereira L.H. (1990). Are there interactions between schistosomes and intestinal nematodes?. Trans. Roy. Soc. Trop. Med. Hyg..

[209] Ferreira C.S., Ferreira M.U., Nogueira M.R. (1994). The prevalence of infection by intestinal parasites in an urban slum in Sao Paulo, Brazil. J. Trop. Med. Hyg..

[210] Kightlinger L.K., Seed J.R., Kightlinger M.B. (1995). The epidemiology of *Ascaris lumbricoides*, *Trichuris trichiura*, and hookworm in children in the Ranomafana rainforest, Madagascar. J. Parasitol..

[211] Booth M., Bundy D.A., Albonico M., Chwaya H.M., Alawi K.S., Savioli L. (1998). Associations among multiple geohelminth species infections in schoolchildren from Pemba Island. Parasitology.

[212] Faulkner H., Turner J., Behnke J., Kamgno J., Rowlinson M.C., Bradley J.E., Boussinesq M. (2005). Associations between filarial and gastrointestinal nematodes. Trans. Roy. Soc. Trop. Med. Hyg..

[213] Nacher M. (2004). Interactions between worm infections and malaria. Clin. Rev. Allergy Immunol..

[214] Druilhe P., Tall A., Sokhna C. (2005). Worms can worsen malaria: Towards a new means to roll back malaria?. Trends Parasitol..

[215] Mwangi T.W., Bethony J.M., Brooker S. (2006). Malaria and helminth interactions in humans: An epidemiological viewpoint. Ann. Trop. Med. Parasitol..

[216] Valero M.A., Santana M., Morales M., Hernandez J.L., Mas-Coma S. (2003). Risk of gallstone disease in advanced chronic phase of fascioliasis: An experimental study in a rat model. J. Infect. Dis..

[217] Valero M.A., Gironès N., Garcia-Bodelon M.A., Periago M.V., Chico-Calero I., Khoubbane M., Fresno M., Mas-Coma S. (2008). Anaemia in advanced chronic fasciolosis. Acta Trop..

[218] Powrie F., Leach M.W., Mauze S., Menon S., Caddle L.B., Coffman R.L. (1994). Inhibition of Th1 responses prevents inflammatory bowel disease in scid mice reconstituted with CD45RBhi CD4+ T cells. Immunity.

[219] McKinley L., Logar A.J., McAllister F., Zheng M., Steele C., Kolls J.K. (2006). Regulatory T cells dampen pulmonary inflammation and lung injury in an animal model of *Pneumocystis* pneumonia. J. Immunol..

[220] Hesse M., Piccirillo C.A., Belkaid Y., Prufer J., Mentink-Kane M., Leusink M., Cheever A.W., Shevach E.M., Wynn T.A. (2004). The pathogenesis of schistosomiasis is controlled by cooperating IL-10-producing innate effector and regulatory T cells. J. Immunol..

[221] Suvas S., Azkur A.K., Kim B.S., Kumaraguru U., Rouse B.T. (2004). CD4+CD25+ regulatory T cells control the severity of viral immunoinflammatory lesions. J. Immunol..

[222] Ezeamama A.E., Friedman J.F., Olveda R.M., Acosta L.P., Kurtis J.D., Mor V., McGarvey S.T. (2005). Functional significance of low-intensity polyparasite helminth infections in anemia. J. Infect. Dis..

[223] Muñoz-Antoli C., Pérez P., Pavón A., Toledo R., Esteba J.G. (2018). Soil-transmitted helminth infections and anemia in schoolchildren from Corn Island Archipelago (RAAS, Nicaragua). Am. J. Trop. Med. Hyg..

[224] Sardinha-Silva A., Alves-Ferreira E.V.C., Grigg M.E. (2022). Intestinal immune responses to commensal and pathogenic protozoa. Front. Immunol..

