# Supplementary material for: Helminth/Protozoan Coinfections in Chronic Fascioliasis Cases in Human Hyperendemic Areas: High Risk of Multiparasitism Linked to Transmission Aspects and Immunological, Environmental and Social Factors
Source: Trop Med Infect Dis. 2025 Aug 11;10(8):224. doi: 10.3390/tropicalmed10080224 (PMC12390233; doi:10.3390/tropicalmed10080224)
Supplement: Supplementary file 1 [file tropicalmed-10-00224-s001.zip › tropicalmed-3761282-supplementary/Supplement S1.pdf]

Tropical Medicine and Infectious Disease

Article

M. Adela Valero, M. Manuela Morales-Suarez-Varela, Davis J. Marquez-Guzman, Rene Angles, Jose R. Espinoza, Pedro Ortiz, Filippo Curtale, M. Dolores Bargues and Santiago Mas-Coma. Helminth/protozoan coinfections in chronic fascioliasis cases in human hyperendemic areas: high risk of multiparasitism linked to transmission, immunological, environmental and social factors. *Tropical Medicine and Infectious Disease* 2025, 10.

SUPPLEMENT 1

The characteristics of the study populations in the four human fascioliasis hyperendemic areas surveyed, including the knowledge on the transmission and epidemiology of the disease, are summarized in the following sections:

A) Northern Bolivian Altiplano (Bolivia)

The Northern Bolivian Altiplano is located between 3,820 and 4,100 m above sea level (asl) and comprises flat plains running between hill mountainous chains between Lake Titicaca and the valley of the capital city, La Paz [3-5]. In the present study, 1,195 Aymara individuals from the Bolivian Altiplano hyperendemic area were analyzed, including the rural villages of Aygachi, Belen Yayes, Caleria, Causaya, Cohana, Corapata, Huacullani, Iquiaca, Kajchiri, Quiripujo, Tuni and Yanarico (Figure 1A).

This area has been multidisciplinary studied for more than three decades [24]. The highest prevalences and intensities in humans have been reported in this area, with local prevalences up to 72% by coprology [70-75] and 100% by serology [76,77], and local intensities with more than 8,000 epg [78]. The transmission of *F. hepatica* is enhanced due to influences of the very high-altitude factor (the term "very high altitude" is used to refer to altitudes higher than 3,200 m asl) [79], with the lymnaeid *Galba truncatula* as the only snail vector species [80].

This area has the extreme climate characteristics typical of the very high-altitude in a subequatorial zone, with great temperature differences throughout a daily period but temperature stability throughout the year (0-16 ºC), a rainy season from October-November to March-April, and a high evapotranspiration rates [81].

The disease transmission is year-long permanent [81], which defines the "altiplanic pattern" of fascioliasis transmission [19]. Sheep and cattle are the main carriers of *F. hepatica* [3], with free-running pigs as the third reservoir species [5]. Donkeys are fourth, playing a role in spreading the disease [4], whereas South American camelids (llama, alpaca) do not contribute to the transmission [6].

An extensive in-depth study focused on, and quantified aspects linked to the human infection risk in this area. A preventive chemotherapy initiative including annual triclabendazole mass treatments was launched [82] and parallelly complemented with One Health measures [24].

B) Peruvian Altiplano (Peru)

A total of 339 Quechua schoolchildren were coprologically surveyed in the irrigation area of Asillo, province of Azangaro, at 3,910 m asl, in the three communities of Jila, Accopata, and Naupapampa. This area is about 140 km northwest of Puno, Peru, located on the northern shore of Lake Titicaca. The irrigation infrastructure covers around 5,000 hectares and includes primary and secondary irrigation canals, as well as drainage canals [83] (Figure 1B).

Climatic characteristics, flat plains, and rural way of life of the inhabitants, are like those of the neighboring Northern Bolivian Altiplano at the other side of Lake Titicaca. Similarly, *F. hepatica* is transmitted by the same species *G. truncatula* [83], and livestock reservoir species are also the same. All in all, this hyperendemic area also fits the "altiplanic pattern" of fascioliasis transmission [9,19]. In this man-made irrigation area, inhabitants collect water for drinking from the irrigation canals but traditionally do not consume freshwater plants [84], as those included in the Aymara diet on the Bolivian side [85]. This indicates that human infection in the Peruvian Altiplano mainly occurs by the ingestion of metacercariae floating in the dinking freshwater.

The coprological surveys resulted in a 24.3% overall mean prevalence, local prevalences ranging between 18.8 and 31.3%, and intensities of up to 2,496 epg, with 196–350 epg (mean: 279 epg) and 96–152 epg (123 epg) as arithmetic and geometric means, respectively [83]. Similar results were obtained in subsequent coprological surveys in the same endemic area [86-88].

C) Cajamarca valley (Peru)

The Department of Cajamarca includes many inter-Andean valleys irrigated by more or less wide and fast-flowing rivers. The 362 subjects were analyzed in six communities at different altitudes of the province of Cajamarca, namely Huayrapongo Grande (altitude: 2627 m asl), Yanamango (2647 m asl), La Colpa (2685 m asl), Llimbe (2764 m asl), Shaullo Grande (2840 m asl) and Santa Rosa de Chaquil (3061 m asl) [89] (Figure 1 C).

Fascioliasis prevalences range 6.7-47.7% (mean 24.4%), showing a positive correlation with increasing altitude [89]. Intensities ranged 24-864 epg (arithmetic mean: 113; geometric mean: 68). *Fasciola hepatica* proved to be the most common helminth, within a parasite spectrum of 11–12 protozoan and 9–11 helminth species [78,89,90]. Similar results were obtained in other surveys, although with lower prevalences [91-98].

In this area, *G. truncatula* is the main vector responsible for the human infection by *F. hepatica*, and *Lymnaea neotropica* and *Pseudosuccinea columella* are above all involved in livestock infection, whereas *Lymnaea schirazensis* is a non-transmitting lymnaeid but of importance due to its similar morphology leading to confusion with the aforementioned local vector species [99-101]. *Galba truncatula* and *L. neotropica* are the most efficient vector species known [102] and are well known in human endemic areas [103].

The disease follows a seasonal transmission related to climate [104-106], which fits the "valley subpattern" of the high-altitude transmission pattern of human fascioliasis [9]. In these valleys, climate factors are typical of the high altitude: temperatures varying daily (July: −5.0 to 23.8 ºC; January: 0.2–24.2 ºC) and monthly (July: 3 ºC; November: 22 ºC); relative humidity of 64%; total yearly precipitation of 769 mm (monthly minimum of 6 mm in July; maximum of 107 mm in October); rainy season from October to April and dry season from May to October [89].

Ethnographic characteristics and sanitary facilities of the inhabitants of the Cajamarca valley proved to be like those in the Northern Bolivian Altiplano [84,107].

D) Behera Governorate, Nile Delta (Egypt)

In the Nile Delta, Lower Egypt, the Behera Governorate has an estimated population of nearly five million inhabitants and includes the localities whose inhabitants were coprologically analyzed (Figure 1 D). Fascioliasis prevalences reported are 52.8% in cattle, 47.5% in buffaloes, and 29.0% in sheep [108]. Children affected by fascioliasis and anemia have been diagnosed in almost every Behera district [109,110].

The 679 subjects analyzed were recruited in four villages of a highly populated area (Figure 1 D): (i) El Aaly and (ii) Bolin (Kafr El Dawar district) are two villages of approximately 10,000 inhabitants surrounded by rice and cotton fields; drainage canals are common, ranging from a large main channel to small irrigation canals; (iii) El Kaza (Hosh Esa district) is a smaller village of approximately 7,000 inhabitants that contains several urban

population clusters and is surrounded by rice fields and irrigation canals of various sizes; (iv) Tiba (Delengate district) is a rural village of approximately 13,000 inhabitants that is also surrounded by rice fields, but is a greater distance from large human populations and large canals [111]. Dwellings are supplied with public water (only 25% of the inhabitants use hand pumps), electricity, and toilets or latrines. Nevertheless, a system for garbage and sewage collection is present in only some areas, and the population in rural houses usually disposes garbage and sewage in canals, rivers, and open fields [112].

Both *F. hepatica* or *F. gigantica* coexist in "local overlap" areas, with *Galba* and *Radix* species in nearby or the same water bodies. Lymnaeid vector species include *G. truncatula* trasmitting *F. hepatica*, *Radix natalensis caillaudi* trasmitting *F. gigantica*, *Pseudosuccinea columella* able to transmit both fasciolid species, and the non-transmitting *Lymnaea schirazensis* [99].

Human fascioliasis prevalences are the highest reported in Africa, ranging 5.2-19.0%, with a mean of 12.8% [111]. Individual intensities which surpass 400 epg are the highest hitherto recorded in the whole Old World [113], ranging 408-2304 epg, with arithmetic and geometric means of 699.5 and 629.07 epg, respectively [113]. Burdens are higher in winter than in summer, which fits a fascioliasis seasonality according to the climatic conditions and a higher infection risk in the second part of the year [114].

Behera flatlands have a typical south Mediterranean arid climate, with highest temperatures in July (23-30 ºC) and lowest in April (14-24 ºC) and very low annual precipitation (around 38 rainy days only, from November to February). Therefore, the extensive fields of crops are irrigated by a wide canal system carrying water from the Nile.

The presence of cows, buffaloes and/or goats in the household was strongly associated with human *Fasciola* sp. infection. This risk increases when these animals are regularly taken to the canal for drinking or cooling down, thereby increasing direct exposure to infection by natural freshwater ingestion [115]. Contrary to the situation in Bolivia and Peru, the absence of pigs in the endemic areas of Behera, due to religious beliefs, should be highlighted.

Freshwater vegetables, terrestrial vegetables needing frequent irrigation, and drinking water from irrigation canals [84], and an association between piped water in the household are involved in human infection by *Fasciola* [115]. Selective treatment is the control strategy used to reduce the risk of multiple intestinal helminths in fascioliasis hyperendemic areas [116].
