# Supplementary material for: Helminth/Protozoan Coinfections in Chronic Fascioliasis Cases in Human Hyperendemic Areas: High Risk of Multiparasitism Linked to Transmission Aspects and Immunological, Environmental and Social Factors
Source: Trop Med Infect Dis. 2025 Aug 11;10(8):224. doi: 10.3390/tropicalmed10080224 (PMC12390233; doi:10.3390/tropicalmed10080224)
Supplement: Supplementary file 1 [file tropicalmed-10-00224-s001.zip › tropicalmed-3761282-supplementary/Supplement S2.pdf]

Tropical Medicine and Infectious Disease

Article

M. Adela Valero, M. Manuela Morales-Suarez-Varela, Davis J. Marquez-Guzman, Rene Angles, Jose R. Espinoza, Pedro Ortiz, Filippo Curtale, M. Dolores Bargues and Santiago Mas-Coma. Helminth/protozoan coinfections in chronic fascioliasis cases in human hyperendemic areas: high risk of multiparasitism linked to transmission, immunological, environmental and social factors. ***Tropical Medicine and Infectious Disease*** **2025, 10.**

SUPPLEMENT 2

Figure. Prevalence of parasites in coinfection in individuals with fascioliasis (F) and non-fascioliasis (non-F) in the four areas analyzed independently: (**A**) Bolivian Altiplano; (**B**) Peruvian Altiplano, and (**C**) Peruvian Cajamarca valley with only *F. hepatica*; (**D**) Nile Delta, Egypt with both *F. hepatica* and *F. gigantica*) (2011-2023). Heatmap of the coinfection rate of intestinal parasites. The grid color represents the coinfection rate of intestinal parasites among individuals with fascioliasis, and the dot color represents the coinfection rate of intestinal parasites among non-fascioliasis individuals. Bigger size and darker color of the circles indicate higher coinfection rates between the pair of parasite species. Bsp = *Blastocystis* sp. Ec = *Entamoeba coli*. Ehi = *Entamoeba histolytica* complex. Eha = *Entamoeba hartmanni*. En = *Endolimax nana*. Ib = *Iodamoeba buetschlii*. Cm = *Chilomastix mesnili*. Gi = *Giardia intestinalis*. Eho = *Enteromonas hominis*. Df = *Dientamoeba fragilis*. Cr = *Cryptosporidium* sp. Eh = *Enteromonas hominis*. Bc = *Balantidium coli*. STH = soil-transmitted-helminths. Sm = *Schistosoma mansoni*. Hn = *Hymenolepis nana*. Tae = *Taenia* sp. Tt = *Trichuris trichiura*. Al = *Ascaris lumbricoides*. An = *Ancylostoma duodenale* and/or *Necator americanus*. Ss = *Strongyloides stercolaris*. Ev = *Enterobius vermicularis*.

Figure A

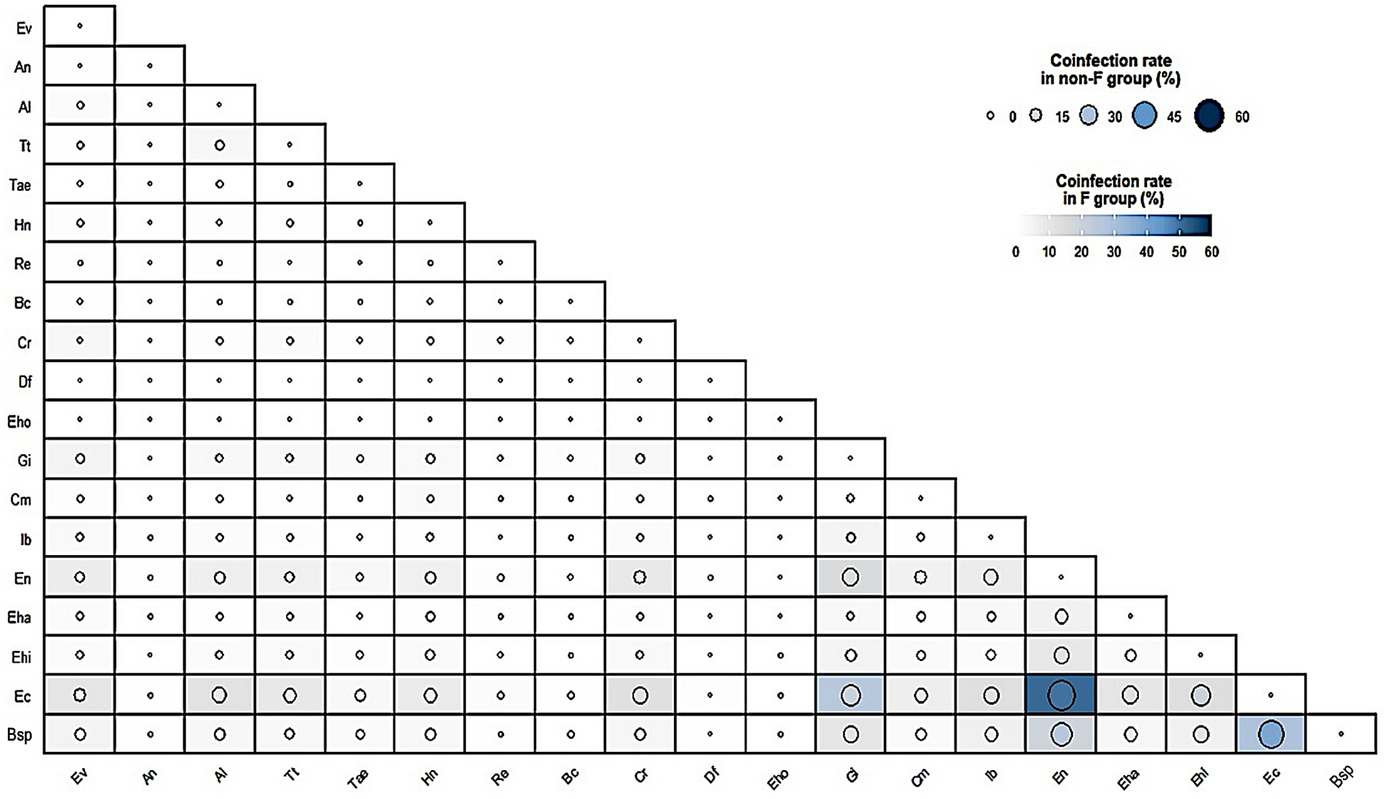

Figure B

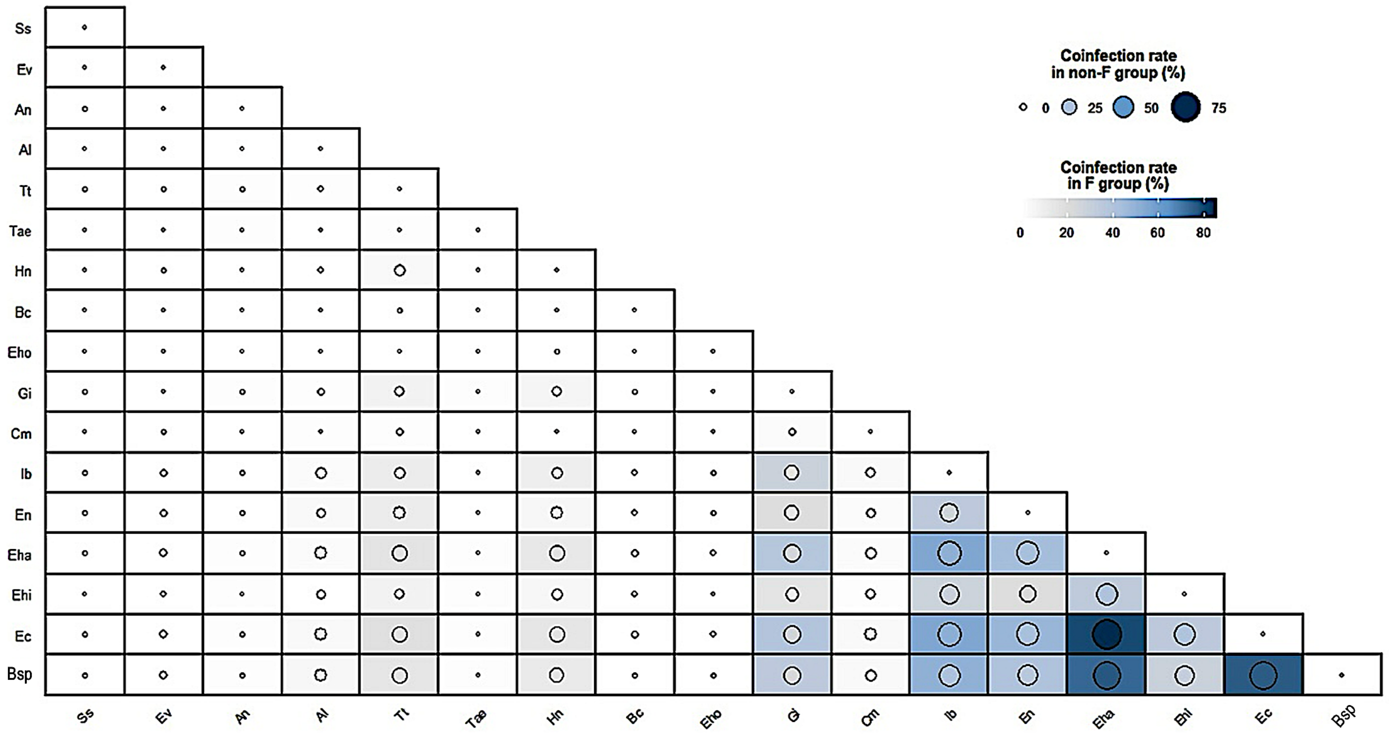

Figure C

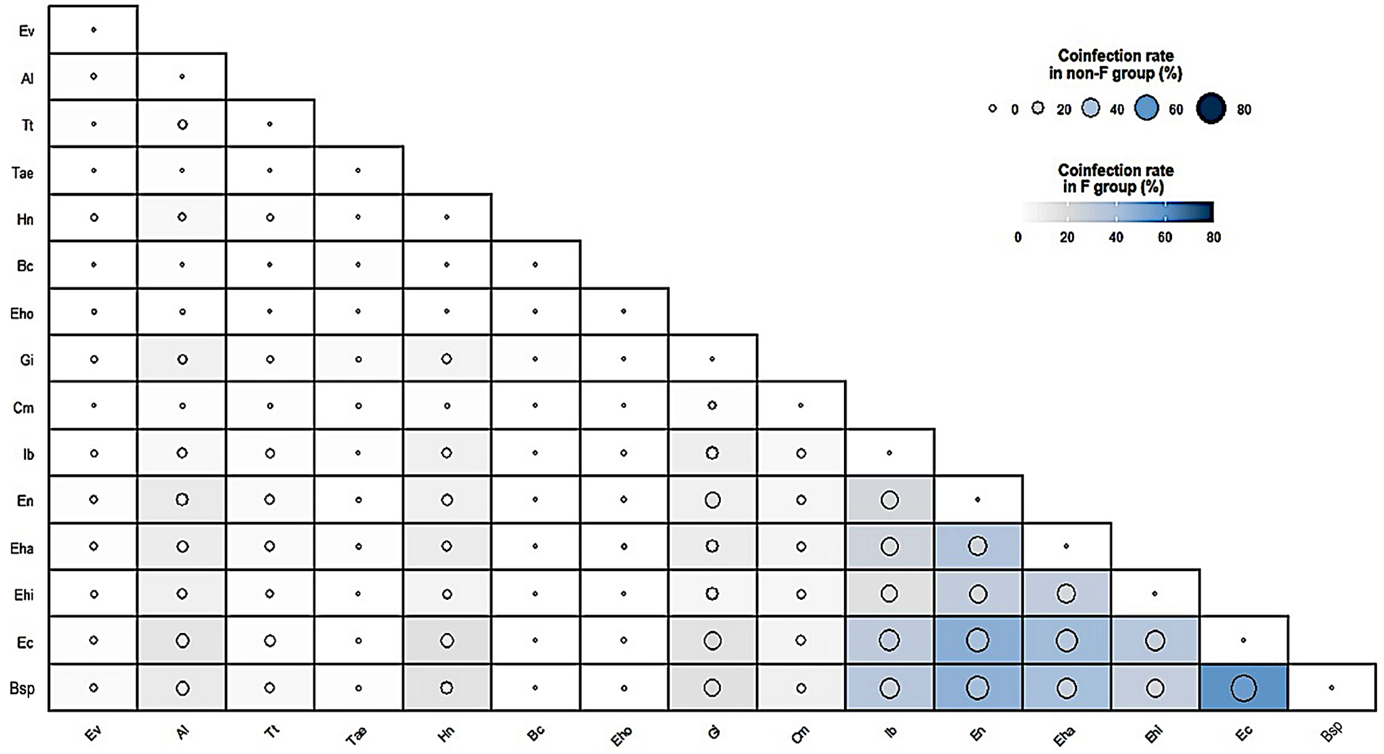

Figure D

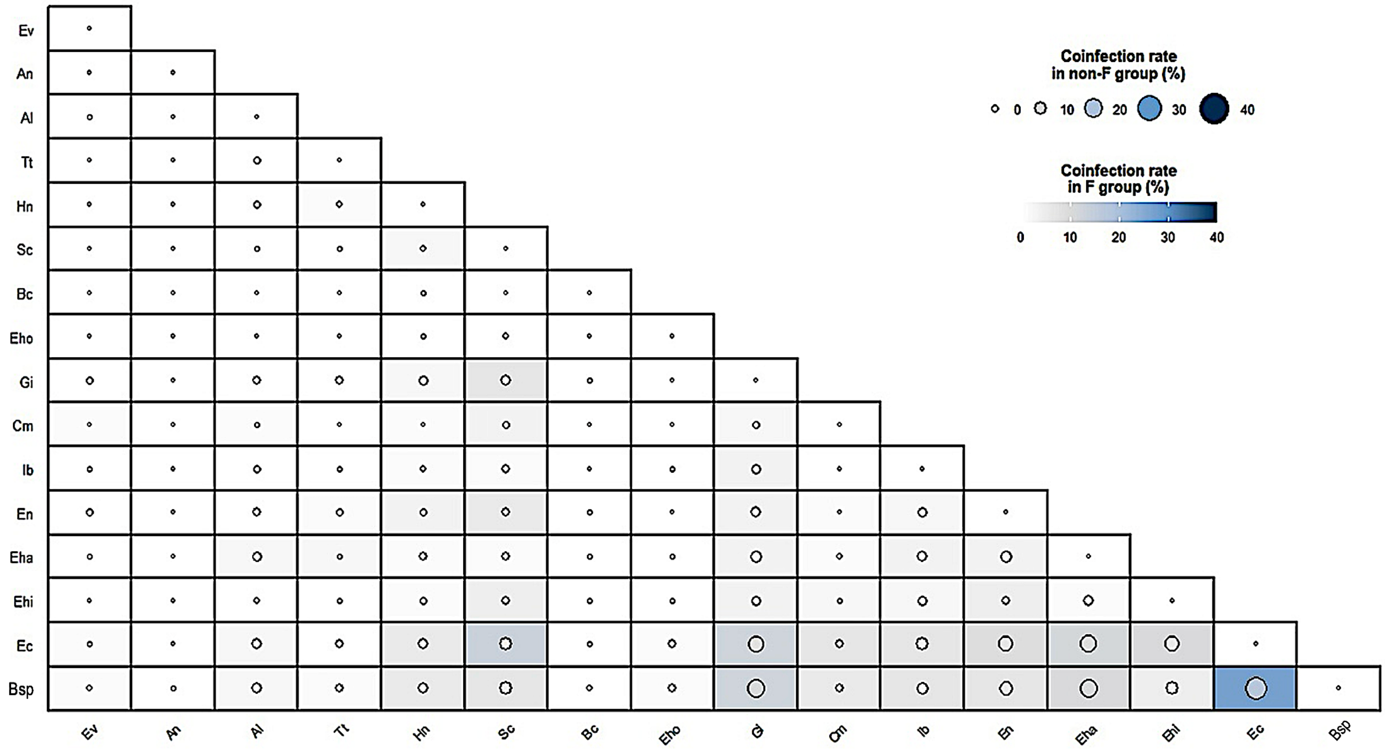
Coinfection rate
in non-F group (%)
0 10 20 30 40
Coinfection rate
in F group (%)
0 10 20 30 40
Ev
An
Al
Tt
Hn
Sc
Bc
Eho
Gi
Cm
Ib
En
Eha
Ehi
Ec
Bsp
