# Supplementary material for: Helminth/Protozoan Coinfections in Chronic Fascioliasis Cases in Human Hyperendemic Areas: High Risk of Multiparasitism Linked to Transmission Aspects and Immunological, Environmental and Social Factors
Source: Trop Med Infect Dis. 2025 Aug 11;10(8):224. doi: 10.3390/tropicalmed10080224 (PMC12390233; doi:10.3390/tropicalmed10080224)
Supplement: Supplementary file 1 [file tropicalmed-10-00224-s001.zip › tropicalmed-3761282-supplementary/Supplement S3.pdf]

Tropical Medicine and Infectious Disease

MDPI

*Article*

**M. Adela Valero, M. Manuela Morales-Suarez-Varela, Davis J. Marquez-Guzman, Rene Angles, Jose R. Espinoza, Pedro Ortiz, Filippo Curtale, M. Dolores Bargues and Santiago Mas-Coma. Helminth/protozoan coinfections in chronic fascioliasis cases in human hyperendemic areas: high risk of multiparasitism linked to transmission, immunological, environmental and social factors. *Tropical Medicine and Infectious Disease* 2025, 10.**

# SUPPLEMENT 3

**Figure**. Adjusted OR for fascioliasis with the reference individuals without fascioliasis. The numbers next to the bars represent the number of individuals with fascioliasis in each group. The lengths of the bars indicate the prevalence rate of each parasite, with the orange bar representing the population characteristics, blue bar representing protozoan species (including *Blastocystis* sp.) and red bar representing helminth species. The black points are the adjusted ORs for fascioliasis and the line indicates the 95% CI. (**A**) Model 1; (**B**) Model 2; (**C**) Model 3; and (**D**) Model 4. Ec = *Entamoeba coli*. Eha = *Entamoeba hartmanni*. En = *Endolimax nana*. Ib = *Iodamoeba buetschlii*. Gi = *Giardia intestinalis*. Sm = *Schistosoma mansoni*. Hn = *Hymenolepis nana*. Tae = *Taenia* sp. STH = Soil-transmitted-helminths. Ss = *Strongyloides stercoralis.*

## Figure A

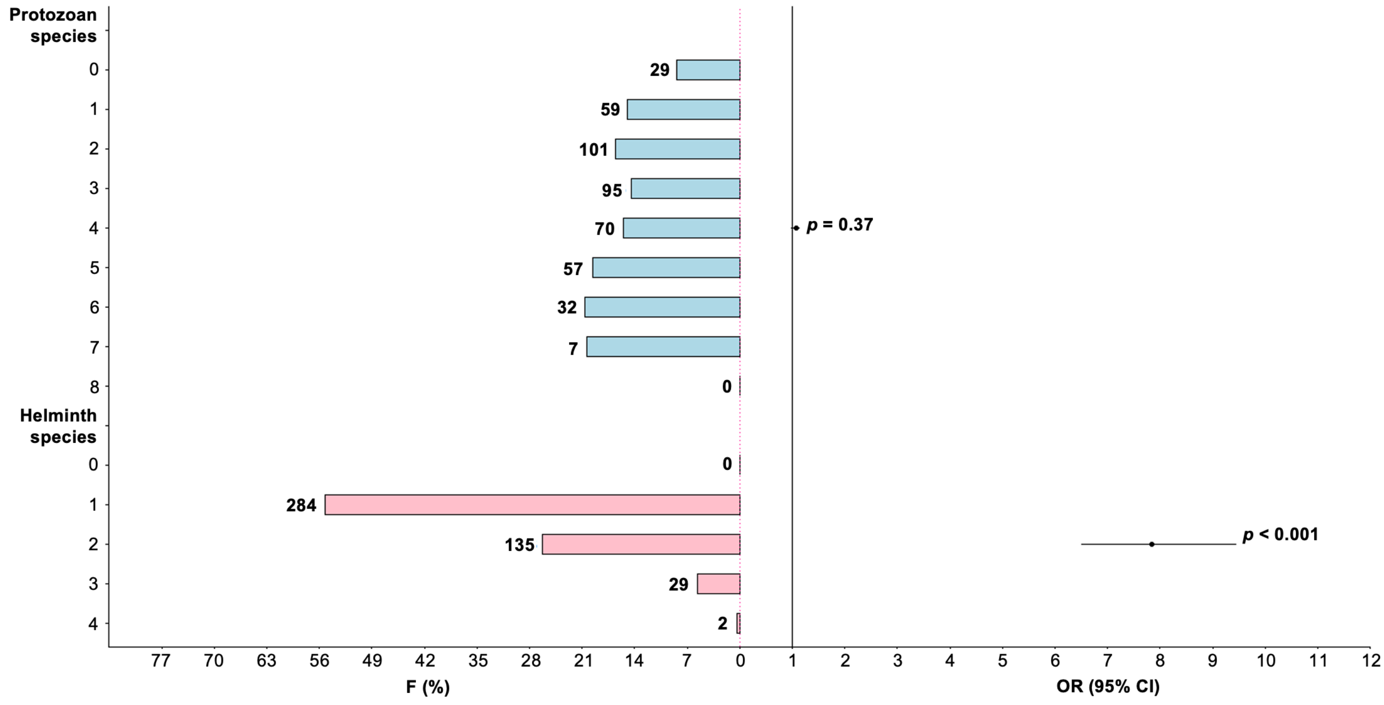

# Figure B

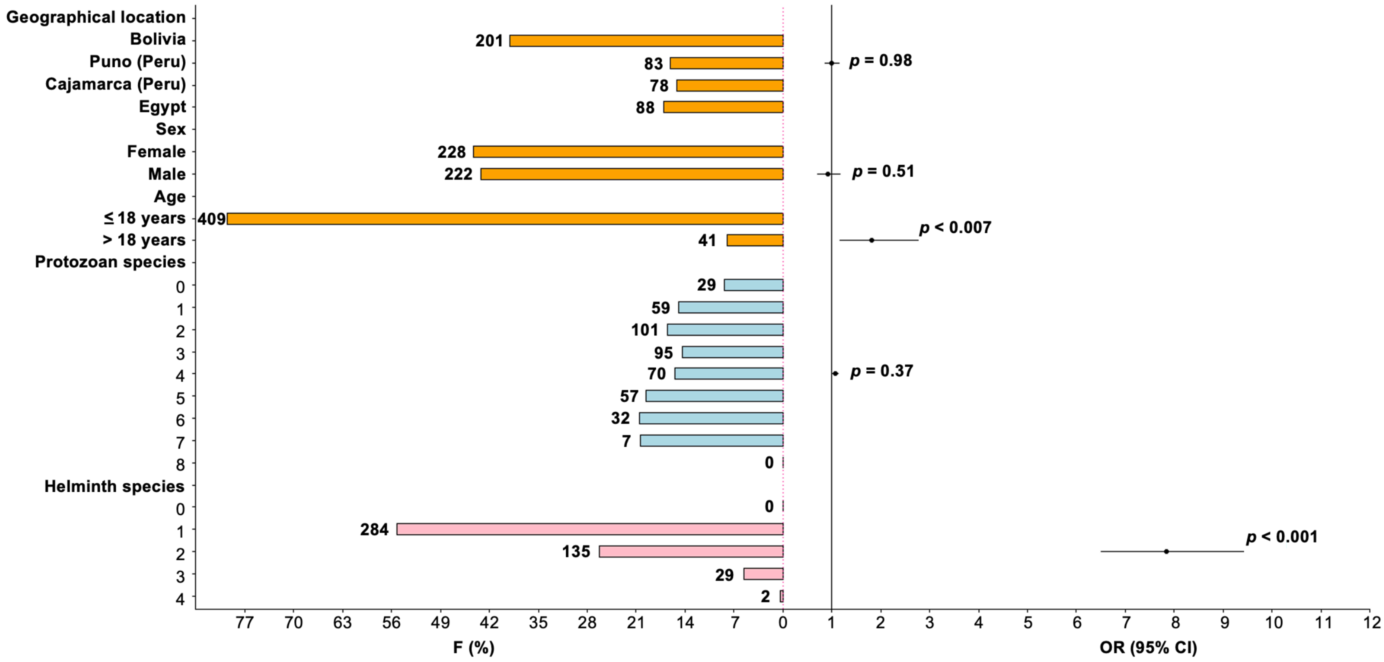


# Figure C

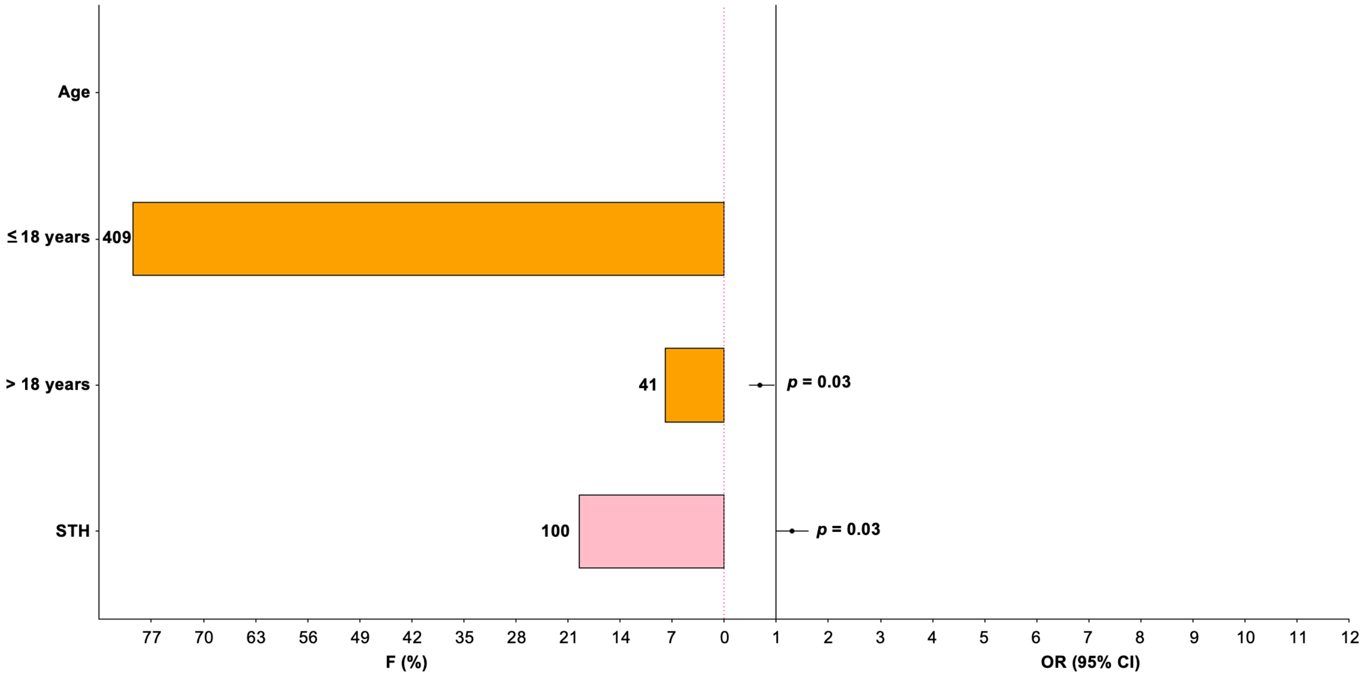

# Figure D

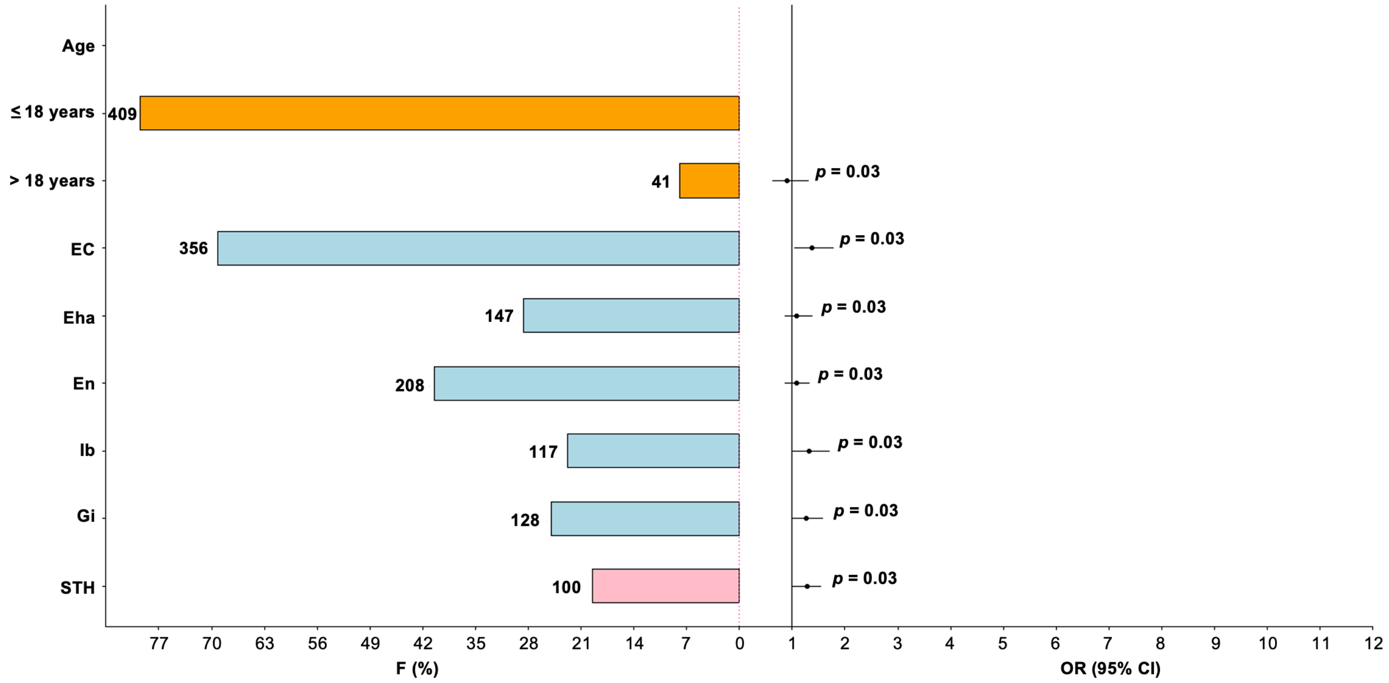

Age
≤ 18 years
> 18 years
EC
Eha
En
Ib
Gi
STH
409
41
356
147
208
117
128
100
p = 0.03
p = 0.03
p = 0.03
p = 0.03
p = 0.03
p = 0.03
p = 0.03
77 70 63 56 49 42 35 28 21 14 7 0
F (%)
1 2 3 4 5 6 7 8 9 10 11 12
OR (95% CI)
